# Design and synthesis of phthalazine-based compounds as potent anticancer agents with potential antiangiogenic activity via VEGFR-2 inhibition

**DOI:** 10.1080/14756366.2019.1642883

**Published:** 2019-07-19

**Authors:** Salwa Elmeligie, Asmaa M. Aboul-Magd, Deena S. Lasheen, Tamer M. Ibrahim, Tamer M. Abdelghany, Sohair M. Khojah, Khaled A. M. Abouzid

**Affiliations:** a Pharmaceutical Organic Chemistry Department, Faculty of Pharmacy, Cairo University, Cairo, Egypt;; b Pharmaceutical Chemistry Department, Faculty of Pharmacy, Nahda University, Beni Suef, Egypt;; c Pharmaceutical Chemistry Department, Faculty of Pharmacy, Ain Shams University, Cairo, Egypt;; d Pharmaceutical Chemistry Department, Faculty of Pharmacy, Kafrelsheikh University, Kafr El-Sheikh, Egypt;; e Pharmacology and Toxicology Department, Faculty of Pharmacy, Al-Azhar University, Cairo, Egypt;; f Biochemistry Department, Faculty of science, King Abdulaziz University, Jedda, Kingdom of Saudi Arabia;; g Department of Organic and Medicinal Chemistry, Faculty of Pharmacy, University of Sadat City, Sadat City, Egypt

**Keywords:** Substituted phthalazines, VEGFR-2 kinase inhibitors, anti-proliferative, apoptosis

## Abstract

In the designed compounds, either a biarylamide or biarylurea moiety or an N-substituted piperazine motif was linked to position 1 of the phthalazine core. The anti-proliferative activity of the synthesised compounds revealed that eight compounds (**6b, 6e, 7b, 13a, 13c, 16a, 16d and 17a**) exhibited excellent broad spectrum cytotoxic activity in NCI 5-log dose assays against the full 60 cell panel with GI_50_ values ranging from 0.15 to 8.41 µM. Moreover, the enzymatic assessment of the synthesised compounds against VEGFR-2 tyrosine kinase showed the significant inhibitory activities of the biarylureas (**12b, 12c and 13c**) with IC_50_s of 4.4, 2.7 and 2.5 μM, respectively, and with 79.83, 72.58 and 71.6% inhibition of HUVEC at 10 μM, respectively. Additionally, compounds (**7b, 13c and 16a)** were found to induce cell cycle arrest at S phase boundary. Compound **7b** triggered a concurrent increase in cleaved caspase-3 expression level, indicating the apoptotic-induced cell death.

## 1. Introduction

Cancer is a leading cause of death worldwide^1^. Epidemiological studies revealed that cancer accounts for one of every five deaths. Moreover, it is estimated that the annual number of deaths due to cancers will increase from 7.6 million in 2008 to 13 million in 2030^2^.

Apoptosis, or programed cell death, plays a crucial role in maintaining the normal body health. However, malignant cells are generally less sensitive to these stresses and tend to evade apoptosis. Genetic regulation, initiation and effector mechanisms can be regarded as stages of apoptosis in its simplest model. Gamma and ultraviolet irradiation, anticancer drugs, deprivation of survival factors such as interleukin-1 (IL-1), or other cytokines that activate “death receptors” are considered as initiators of apoptosis^3^. Hence, the identification of apoptosis inducers has evolved as an attractive approach for the development of potential anticancer agents.

From another point of view, angiogenesis, the process of sprouting of new blood vessel formations from pre-existing ones, is also regarded as an important hallmark of cancer development. Vascular endothelial growth factor family (VEGFs) are known to be one of the key regulators of angiogenesis. Their biologic effects is exerted by binding to extracellular domain.

Multiple reports have detailed several small-molecule inhibitors of VEGFR-2 acting by binding to the ATP-binding site in the intracellular kinase domain resulting in diminished VEGF signal transduction, these inhibitors may be broadly categorised into two main types: type I; inhibitors represent ATP competitors that generally bind in or around the catalytic site of the kinase in its active conformation, in the region originally occupied by the adenine moiety of ATP^4^. On the other hand, type-II inhibitors stabilise the inactive conformation of the enzyme derived upon the movement of the DFG motif (i.e. Asp-Phe-Gly), hence, they exploit new interactions with the lipophilic pocket revealed in this new rearrangement^5^.

**Figure 1.**
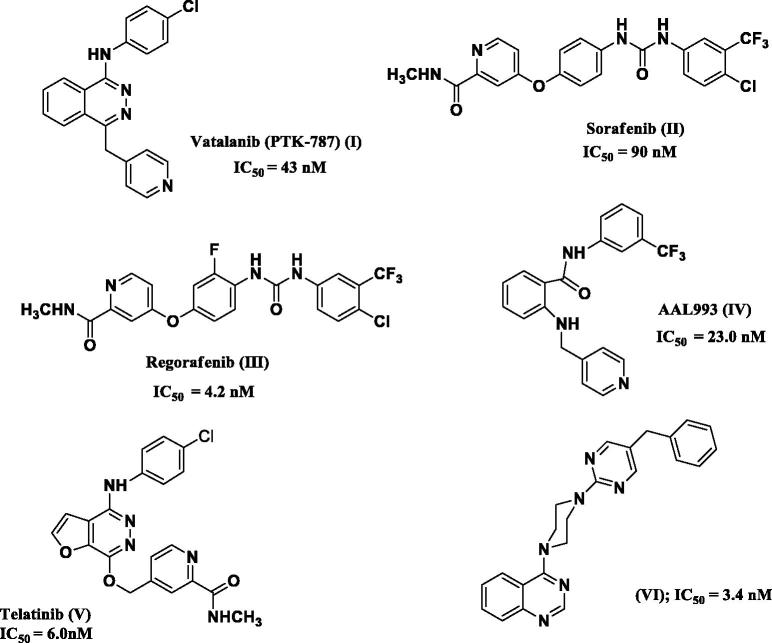
Structures of some known VEGFR-2 kinase inhibitors.

In the past few years, a number of VEGFR-2 inhibitors have proven success as targeted anticancer therapeutics. Among these is the 1,4-disubstituted phthalazine inhibitor, vatalanib. (PTK-787) **(I)** (IC_50_ VEGFR-2 = 43 nM) (Figure 1)^6^.

The biarylurea-based inhibitor, sorafenib **(II) (**IC_50_-VEGFR-2 = 90 nM) (Figure 1), has been approved for the treatment of advanced renal cell carcinoma^7^. Sorafenib was proved to suppress tumour proliferation, metastasis and angiogenesis; in addition, it was also found to be capable of inducing apoptosis, both *in vitro* and *in vivo,* in a host of cancer cells. However, the underlying mechanisms seemed to vary depending on cancer cell types^8–10^. Interest in developing novel biarylurea-based VEGFR-2 inhibitors has been increasingly highlighted with the clinical success of sorafenib and its analogs, such as regorafenib **(III)** ^11^.

Moreover, the biarylamide, (AAL993) **(IV),** a hybrid-design type-II inhibitor derived from the type-I inhibitor vatalanib, was reported as a potent inhibitor of VEGFR family with VEGFR-2 IC_50_ of 23 nM^12^.

Also, telatinib (**V**), the furopyridazine derivative, emerged as a potent and orally available inhibitor of VEGFR-2, VEGFR-3 with IC_50_s 6 and 4 nM, respectively. It is currently in clinical trials for gastric and colorectal cancer^13^.

From another point of view, the piperazinyl moiety, the small rigid heterocyclic motif, has been successfully incorporated in several potent kinase inhibitors^14–17^. The aryl piperazine-based derivative **(VI)** (Figure 1) was reported to exhibit potent single-digit nanomolar inhibitory activity on specific kinases^18^ ^,^ ^19^.

## 2. Rationale and design

**Figure 2.**
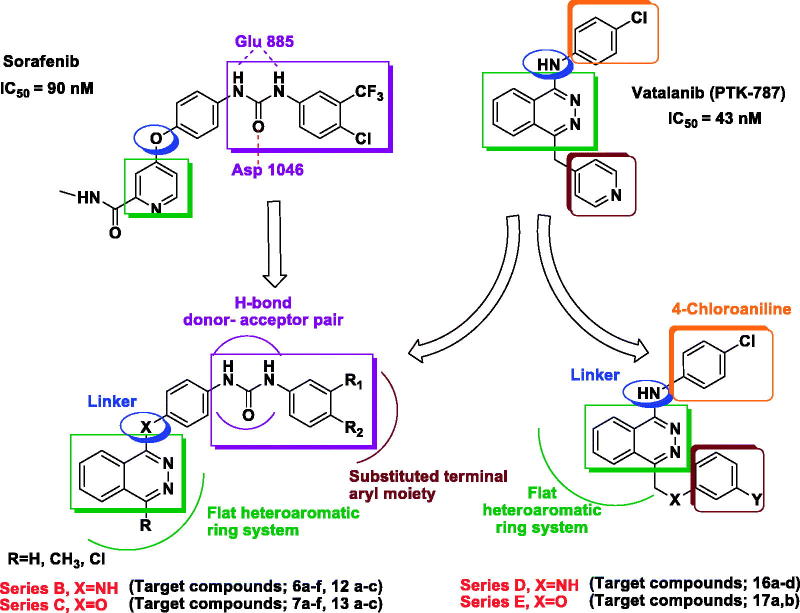
Strategy for the design of the target compounds.

Based on the above findings, three series of phthalazine-based derivatives linked to a biarylamide **(series A)** or biarylurea tail **(series B, C)** at position 1 of the phthalazine core via an amino or ether linkage were designed and synthesised as potent anti-proliferative derivatives as well as apoptosis inducers in tumour cells. Different substitution patterns were introduced to the terminal aromatic ring aiming to better occupy the hydrophobic pocket revealed by the kinase in its DFG-out conformation (Figure 2). The design of these series was based on attaining new hybrid structures from sorafenib and vatalanib while maintaining the structure activity relationships as well as common pharmacophoric features shared by several VEGFR inhibitors which involve the following three features; **(1)** a flat heteroaromatic ring system to better occupy the ATP-binding region of the kinase, **(2)** a hydrogen bond donor–acceptor pair represented by the urea or amide moieties that usually form hydrogen bonds with Glu 885 and Asp 1046 residues, **(3)** a substituted terminal aryl moiety to occupy the hydrophobic pocket revealed by the movement of the DFG motif of the kinase in its inactive conformation^20^.

Furthermore, two other series of vatalanib analogs **(series D, E)** were designed having a 4-chloroaniline moiety at position 1 of the phthalazine and an anilino or phenoxy motif linked to the position 4 via a methylene spacer.

In addition, a sixth series of substituted piperazine-based derivatives (**series F**) was prepared as well, via linking the two major fragments: the phthalazine core recognised as a kinase-privileged fragment, to the piperazine moiety aiming to offer more rigidity and to facilitate deriving spatial–activity relationships^15^.

## 3. Chemistry

**Scheme 1.**
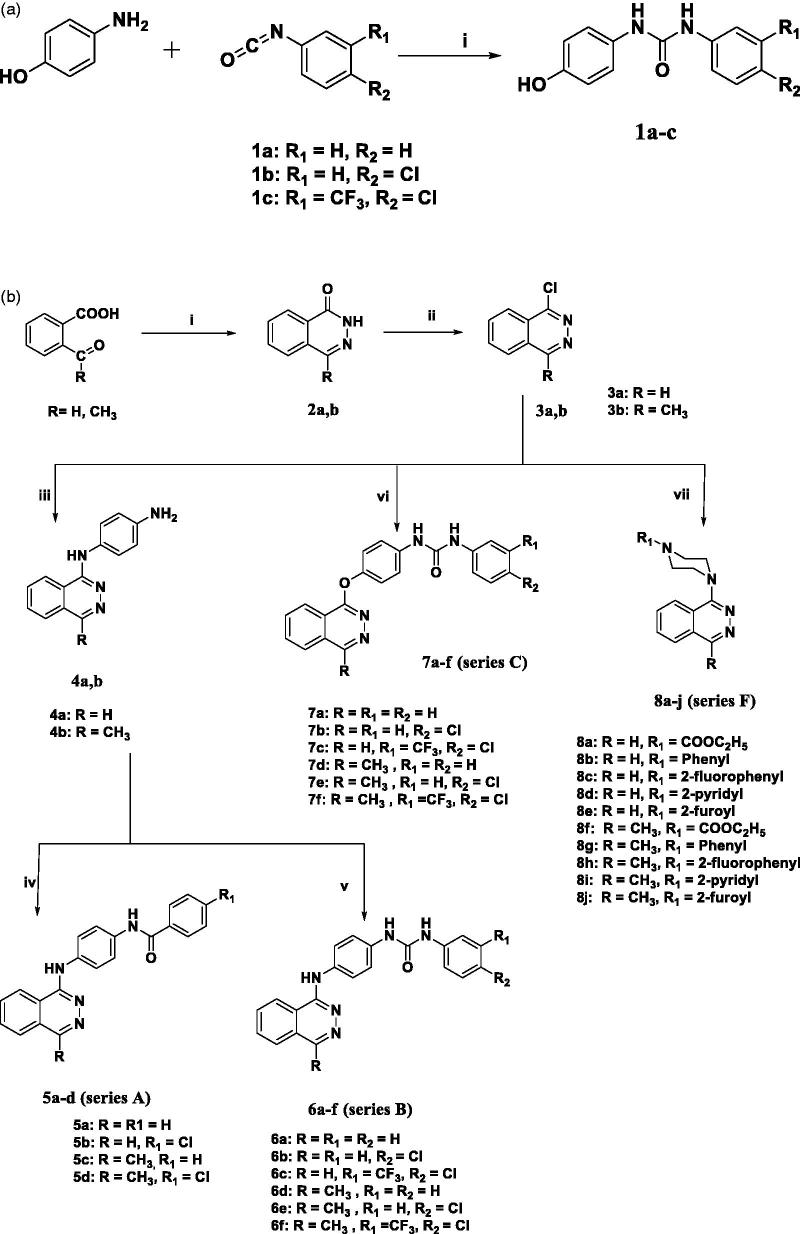
(a) Reagents and conditions: (i) dioxane, rt, 1 h. (b) Reagents and conditions: (i) NH_2_NH_2_ 99%, propanol, 2 h (ii) POCl_3_, reflux 70 °C, 2 h (iii) *p*-phenylenediamine, butanol, 110 °C, 1 h, (iv) benzoyl chlorides, acetonitile, triethylamine, 6 h (v) arylisocyanates, DMF, reflux, 8 h, (vi) **1a–c**, Cs_2_CO_3_, acetonitrile, reflux, 6 h, (vii) piperazines, K_2_CO_3_, KI, ethanol, reflux, 3 h.

**Scheme 2.**
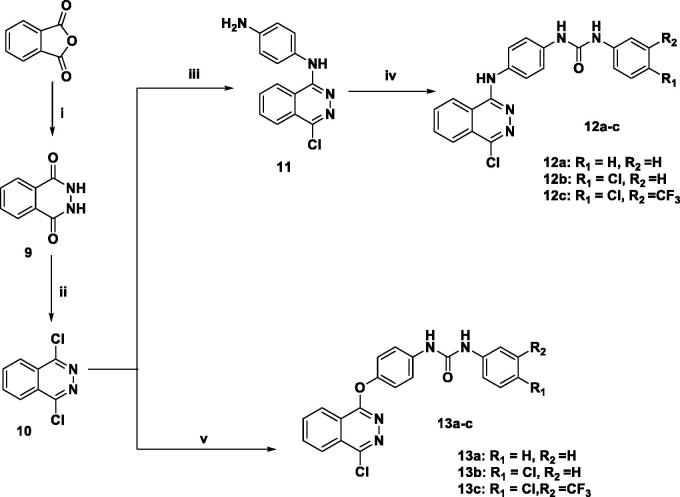
Reagents and conditions: (i) NH_2_NH_2_ acetic, reflux, 3 h (ii) POCl_3_, reflux 110 °C, 1 h (iii) *p*-phenylenediamine, butanol, 110 °C, 1 h, (iv) phenylisocyanates, DMF, reflux, 8–10 h, (v) **1a–c**, Cs_2_CO_3_, acetonitrile, reflux, 6–8 h.

**Scheme 3.**
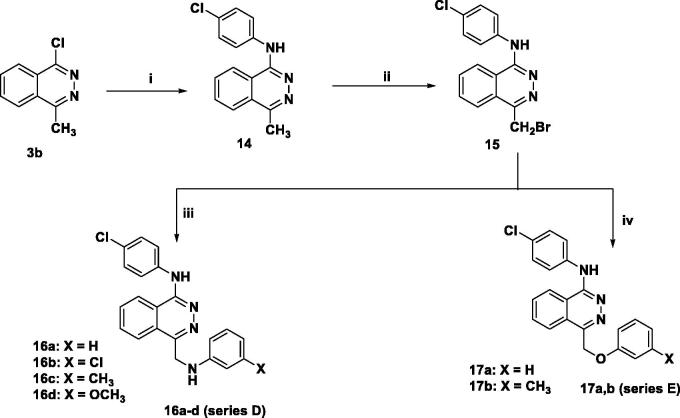
Reagents and conditions: (i) aniline derivatives, butanol, K_2_CO_3_, KI, reflux, 3 h (ii) NBS, dibenzoyl chloride, reflux, 24 h (iii) aniline derivatives, acetone, K_2_CO_3_, KI, reflux, 6 h (iv) phenol derivatives, NaH, acetone, reflux, 8 h.

The pathways adopted for the synthesis of the new substituted phthalazine derivatives are depicted in Schemes (1–3). The key intermediate 1-aryl-3–(4-hydroxyphenyl)urea **(1a–c)**, were prepared following the literature methods as illustrated in (Scheme 1(a))^21^. On the other hand, cyclisation of the respective *o*-substituted benzoic acid derivative with hydrazine hydrate afforded the corresponding phthalazinones **(2a,b)**. Chlorination of **2a,b** with phosphorous oxychloride afforded the corresponding 1-chlorophthalazines **(3a,b),** which were used to prepare the target series of 1-substituted phthalazines via reaction with various nucleophiles. Thus, the reaction of **3a,b** with *p*-phenylenediamine in butanol via the displacement of the chloro atom of phthalazine with the suitable nitrogen nucleophile such as *p*-phenylenediamine afforded the *N* ^1^-(phthalazin-1-yl)benzene-1,4-diamines **(4a,b)**. The latter compounds **(4a,b)** were further reacted with the respective benzoyl chloride in acetonitrile, in the presence of triethylamine to afford the target phthalazines bearing the biarylamide tail **(5a–d) (series A)**. Moreover, the reaction of **(4a,b)** with different arylisocyanates in DMF yielded the biarylureas **(6a–f) (series B)**. On the other hand, 1-aryl-3–(4-(4-substituted phthalazin-1-yloxy)phenyl)ureas **(7a–f) (series C)** were obtained via reacting the respective 1-chlorophthalazines **(3a,b)** with the appropriate intermediates **(1a–c)** in refluxing acetonitrile. This latter reaction proceeds via *ipso* addition by the nucleophile that gives an anion with a highly delocalised charge then followed by leaving of the chlorine atom of the respective 1-chlorophthalazines **(3a,b)** to afford the alkoxyphthalazines **(7a–f)**. Refluxing the 1-chlorophthalazines **(3a,b)** with the appropriate piperazine derivative in ethanol yielded the target phthalazines bearing the substituted piperazinyl tail **(8a–j) (series F)** in good yields (Scheme 1(b)).

As for the synthesis of the biarylureas (**12a–c and 13a–c**) having a chloro substituent at 4 position of the phthalazine (Scheme 2), it was started by refluxing phthalic anhydride with hydrazine hydrate in acetic acid, followed by reflux with POCl_3_ to give the 1,4-dichlorophthalazine intermediate **(10)**. Thereafter, the target compounds **(12a–c and 13a–c)** were obtained in a manner similar to that adopted in Scheme 1(b).

Compounds **(16a–d, and 17a,b) (series D, E)** were synthesised via initial bromination of the key intermediate **(14)** with NBS in presence of catalytic amount of dibenzoyl peroxide. The reaction of the bromo derivative **(15)** with the respective aniline or phenol in acetone then afforded the titled compounds (Scheme 3).

## 4. Results and discussion

### 4.1. Biological evaluation

#### 4.1.1. *In vitro* anticancer screening at full NCI 60 cell panel

Aiming to evaluate the general antitumour activity of the synthesised phthalazines, the structures of the final compounds were submitted to National Cancer Institute “NCI” (www.dtp.nci.nih.gov), Bethesda, Maryland, USA. *In vitro* NCI anticancer screening involves two-stage process, the first stage started with the evaluation of the selected candidates against the full NCI 60 cell lines panel representing leukaemia, non-small cell lung cancer, melanoma, colon, CNS, breast, ovarian, renal and prostate cancers at a single dose of 10 µM. NCI selection is based on degree of structure variation and application of computer modelling techniques to prioritise compounds based on their ability to add diversity to the NCI small molecule compound collection.

Eighteen of the submitted phthalazines (**5b, 5d, 6b, 6e, 7b, 7e, 8a, 8d, 8f, 8g, 8h, 8i, 13a, 13b, 13c, 16a, 16d, and 17a)** were selected by NCI under drug discovery programme for screening against the full NCI 60 cell panel at single dose 10 µM. Among the investigated 18 compounds, eight compounds (**6b, 6e, 7b, 13a, 13c, 16a, 16d and 17a)** were selected by NCI for further screening at 5-log dose molar range due to their prominent cell growth inhibition against various cell lines.

##### 4.1.1.1. Primary *in vitro* antineoplastic single-dose assay

Primary *in vitro* single-dose anticancer assay was evaluated against full NCI 60 cell panel. Results for each compound were presented as a mean graph of the growth per cent of the treated cells compared to the untreated control cells. Eight of the investigated phthalazine-based derivatives **(6b, 6e, 7b, 13a, 13c, 16a, 16d and 17a)** showed a distinctive pattern of sensitivity against various NCI cell lines (Tables S1 and S2 in Supplementary material **)**. Thus, the biarylurea-based derivatives having a terminal 4-chloro substituent (**6b, and 6e)** exhibited remarkable broad spectrum cell growth inhibition above 90% against various cell lines including leukaemia, non-small cell lung cancer, melanoma, prostate cancer and breast cancer cell lines. Compound **6e** even exhibited lethal effect (above 100% inhibition) against several leukaemia, melanoma, renal cancer cell lines. Interestingly, it showed highly potent inhibitory or lethal effects against most of the tested leukaemia and melanoma cell lines.

Furthermore, their analogue (**7b)** linked to the phthalazine nucleus via an ether linkage also showed excellent broad spectrum inhibitory or lethal activity against most of the tested NCI cell lines representing all the nine tumour subpanels specially those of leukaemia, colon, melanoma and breast cancer cell lines, with mean growth inhibition of 106.99%. However, its analogue **(7e)** having a methyl substituent at 4-position of phthalazine core only showed moderate cell growth inhibition against leukaemia and breast cell lines (Table S3 in Supplementary material).

Thus, the unsubstituted biarylurea derivative **(13a)** displayed broad spectrum cell growth inhibition above 90% against various cell lines including non-small cell lung cancer, melanoma, CNS cancer, melanoma and breast cancer cell lines. Interestingly, its disubstituted analogue (**13c**) exhibited an excellent broad spectrum lethal activity against most of the tested NCI cell lines representing all the nine tumour subpanels specially those of non-small lung cancer, colon, CNS and melanoma cell lines, with mean growth inhibition of 145.09%. Surprisingly, the 4-chloro substituted derivative (**13b**) did not show any significant cell line growth inhibition (Table S3 in Supplementary material).

For the vatalanib analogues having a 4-anilino **(16a, and 16d)** or 4-phenoxy substituent **(17a)** linked to the phthalazine via methylene spacer; It was found that all the three tested derivatives displayed remarkable broad spectrum cell growth inhibition above 90% against various cell lines including leukaemia, melanoma and breast cancer cell lines, with the meta methoxy analogue **(16d)** showing the most prominent inhibition against most of the tested cell lines with mean growth inhibition of 116.00%.

Neither of the biarylamides (**5b, and 5d**) nor the piperazines (**8a, 8d, 8f, 8g, 8h and 8i)** showed any significant cell line growth inhibition under the same test conditions (Table S3 in Supplementary material).

##### 4.1.1.2. *In vitro* 5 log dose full NCI 60 cell panel assay

Eight compounds **(6b, 6e, 7b, 13a, 13c, 16a, 16d and 17a)** were selected by NCI for further 5 log dose screening against full NCI 60 cell panel. All the 60 cell lines, representing nine tumour subpanels (leukaemia, non-small cell lung cancer, colon cancer, CNS cancer, prostate cancer, melanoma, ovarian cancer, renal cancer and breast cancer) which were incubated at five different concentrations (0.01, 0.1, 1, 10 and 100 µM) of each of the test compounds. The outcomes were used to create log concentration *vs* % growth inhibition curves, then three response parameters (GI_50_, TGI and LC_50_) were calculated for each cell line, (Tables S4 and S5 in Supplementary material).

All investigated biarylureas **(6b, 6e, 7b, 13a, and 13c)** and vatalanib analogues **(16a, 16d, and 17a)** revealed potent antiproliferative activity against most of the tested cell lines representing the nine different subpanels with GI_50_ values between 0.15–8.41 µM, except for **16a** which was insensitive to prostate and MCF-7 breast cancer cell lines.

With regard to the sensitivity against the tested cell lines, compound (**7b and 13c)** exhibited the highest broad spectrum submicromolar inhibitory activity against most of the tested cell lines specially those of leukaemia, colon, melanoma, breast cancer and renal (**13c only**) cell panels with GI_50_ values of 0.15–2.81 µM and 0.2–2.66 µM for **7b and 13c,** respectively. It is worth noting that **13c** showed submicromolar GI_50_s against all tested leukaemia and melanoma cell lines.

As for the selectivity of the test compounds towards some specific tumour subpanels, which is calculated based on the ratio obtained by dividing the full panel MID (the average sensitivity of all cell lines towards the test agent) by their individual subpanel MID (the average sensitivity of all cell lines of a particular subpanel towards the test agent)^24^ ^,^ ^25^. As per this criterion, the test compounds were regarded to be more selective against leukaemia, renal, melanoma, colon and breast cancer subpanels.

#### 4.1.2. *In vitro* cytotoxic activity against MCF-7, HCT-116 and HepG-2 cancer cell lines

The growth inhibition of the rest of synthesised compounds (not selected by NCI) **(5a, 5c, 6a, 6c, 6d, 6f, 7c, 7d, 7f, 8b, 8c, 8e and 8j)** was also evaluated against two specific cell lines, namely MCF-7 and HCT-116 using doxorubicin as a reference drug. In addition, compounds (**12a, 12b, 12c, 16b, 16c and 17b)** were evaluated against a third cell line (HepG-2) These first two cell lines were selected based on the sensitivity of the previously NCI-tested derivatives towards them amongst the 60 NCI panel. The growth inhibition is expressed as the median growth inhibitory concentration (IC_50_) which corresponds to the concentration required for 50% inhibition of cell viability and are provided in (Table S6 in supplementary material).

Investigation of the results of the growth inhibition of the tested molecules revealed that the biarylurea-based phthalazines **(series B and C)** generally exhibited moderate to significant cytotoxicity on both cell lines. This was relatively in accordance with the NCI findings for the same series of compounds.

Further analysis of the inhibitory results within the biarylurea-based derivatives linked to phthalazine core via an NH-linker **(series B)** revealed that, the 4-chloro-3-trifluoromethyl derivatives **(6c and 6f),** bearing an electron withdrawing substitution pattern similar to that of sorafenib, displayed enhanced growth inhibition on both cell lines compared to their unsubstituted analogues **(6a and 6d)** (IC_50_s = 6.2, 6.0 and 3.2, 3.1 µM compared to 67.6, 91.2 and 67.6 and 57.5 µM respectively).

As for the biarylureas bearing an ether linker **(series 7),** the 4-chloro-3-trifluoromethyl derivative (**7c)** similarly displayed significant inhibiton on both cell lines, (IC_50_s = 2.5 and 2.6 µM). Surprisingly, its methyl analogue **(7f)** demonstrated weaker activity (IC_50_s = 31 and 49 µM) compared to its unsubstituted analogue (**7d**, IC_50_s = 7.8 and 7.2 µM). However, the amide-based derivatives **(series A)** and the piperazine-based derivatives have not shown any interesting cell line inhibition which was also consistent our previous results.

Unfortunately, neither the 4-chlorophthalazines (**12a–c**) nor the vatalanib analogues (**series D and E**) showed any significant cell line inhibition (Table S6 in Supplementary Material).

#### 4.1.3. *In vitro* VEGFR-2 tyrosine kinase activity

##### 4.1.3.1 VEGFR-2 kinase activity at single-dose 10 μM concentration

The VEGFR-2 tyrosine kinase assay was performed in an attempt to investigate the mechanism of potent antiproliferative activity exerted by the synthesised compounds. The VEGFR tyrosine kinase assays were performed at BPS Bioscience (www.bpsbioscience.com). All the synthesised compounds representing the six series were evaluated for their ability to inhibit VEGFR-2 tyrosine kinase at single dose of 10 µM.

Investigation of the results of VEGFR-2 inhibitory activities among the synthesised phthalazines revealed that: Within the biaryl ureas **(series B, C):** derivatives having a 4-chloro-3-trifluoromethyl substitution pattern on the terminal phenyl ring similar to that of sorafenib **(6c, 6f, 7c, 12c and 13c)** tended to exhibit enhanced VEGFR-2 inhibitory activity as excepted from cell line results compared to their monosubstituted or unsubstituted analogues.

In accordance with cell lines results, among these biaryl ureas, the introduction of a 4-chloro group to the phthalazine core replacing the 4-CH_3_ or unsubstituted derivatives, resulted in favourable increase in VEGFR-2 inhibitory activity **(12a–c, 13a–c** compared to **6a–f, 7a–f),** which might be attributed to enhanced lipophilicity of the chloro group. Thus, compounds **6c, 12b, 12c and 13c** showed 70–100% inhibition at 10 µM concentration (Table 1).

**Table 1.** The VEGFR-2 inhibition per cent of the synthesised phthalazines (series A, B, C) at 10 μM concentration.
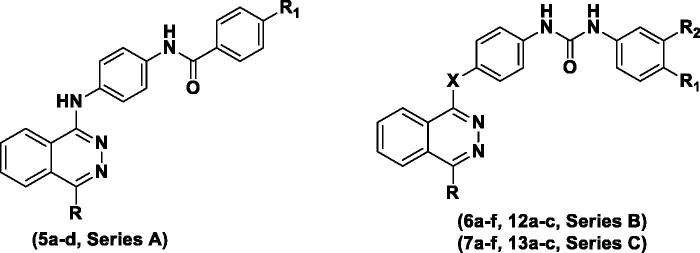

Cpd No	X	*R*	*R_1_*	*R_2_*	% inhibition
**5a**	–	H	H	–	4
**5b**	–	H	Cl	–	8
**5c**	–	CH_3_	H	–	12
**5d**	–	CH_3_	Cl	–	15
**6a**	NH	H	H	H	13
**6b**	NH	H	Cl	H	20
**6c**	NH	H	Cl	CF_3_	**70**
**6d**	NH	CH_3_	H	H	15
**6e**	NH	CH_3_	Cl	H	19
**6f**	NH	CH_3_	Cl	CF_3_	**32**
**12a**	NH	Cl	H	H	**26**
**12b**	NH	Cl	Cl	H	**70**
**12c**	NH	Cl	Cl	CF_3_	**78**
**7a**	O	H	H	H	5
**7b**	O	H	Cl	H	13
**7c**	O	H	Cl	CF_3_	**47**
**7d**	O	CH_3_	H	H	10
**7e**	O	CH_3_	Cl	H	14
**7f**	O	CH_3_	Cl	CF_3_	21
**13a**	O	Cl	H	H	**24**
**13b**	O	Cl	Cl	H	**38**
**13c**	O	Cl	Cl	CF_3_	**100**
**Staurosporin**					**100**

The bold values are the most active compounds.

However, the vatalanib analogues **(16a–d and 17a,b) (series D, E),** they generally exhibited weak to moderate activity, with the derivatives having unsubstituted anilino or phenoxy moiety **(16a, 17a)** showing relatively better activity than their meta substituted analogues (Table 2).

**Table 2.** The VEGFR-2 inhibition per cent of the synthesised phthalazines (series D, E, F) at 10 μM concentration. 
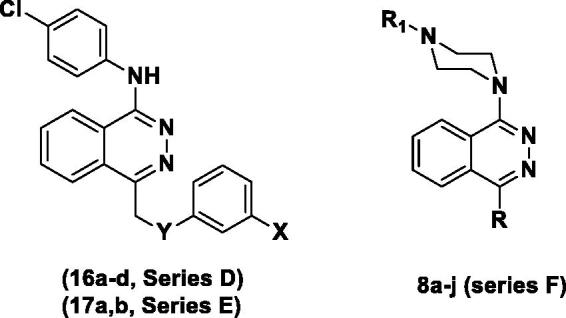

Cpd No	Y	X	*R*	*R*_1_	% inhibition
**16a**	NH	H	–	–	**47**
**16b**	NH	Cl	–	–	21
**16c**	NH	CH_3_	–	–	19
**16d**	NH	OCH_3_	–	–	24
**17a**	O	H	–	–	**31**
**17b**	O	CH_3_	–	–	15
**8a**	**–**	**–**	H	ethoxycarbonyl	13
**8b**	**–**	**–**	H	phenyl	8
**8c**	**–**	**–**	H	2-fluorophenyl	9
**8d**	**–**	**–**	H	pyridyl	5
**8e**	**–**	**–**	H	2-furoyl	9
**8f**	**–**	**–**	CH_3_	ethoxycarbonyl	14
**8g**	**–**	**–**	CH_3_	phenyl	13
**8h**	**–**	**–**	CH_3_	2-fluorophenyl	9
**8i**	**–**	**–**	CH_3_	pyridyl	6
**8j**	**–**	**–**	CH_3_	2-furoyl	10
**Staurosporin**	**–**	**–**	–	–	100

The bold values are the most active compounds.

As expected from the cell lines results, neither of the amide-based derivatives **(5a–d) (series A)** nor the piperazines **(8a–j)(series F)** showed any significant VEGFR-2 inhibition in accordance with cell lines results.

##### 4.1.3.2. Measurement of potential VEGFR-2 inhibitory activity (IC_50_)

**Figure 3.**
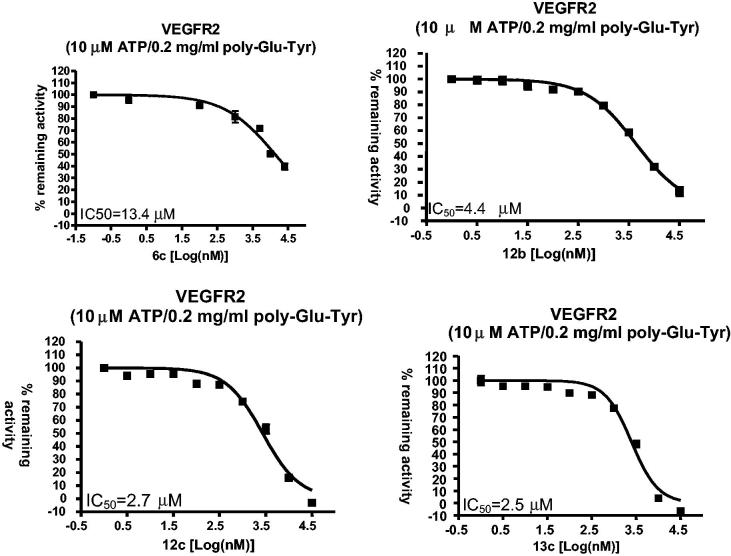
IC_50_ curves for compounds (**6c**, **12b, 12c** and **13c**) against the VEGFR-2 kinase.

Further investigation for the promising candidates, **(6c, 12b, 12c, 13c),** which exhibited VEGFR-2 inhibition percent above 70% at 10 µM concentration, were evaluated at five different concentrations (0.01, 0.1, 1, 10 and 100 µM) to calculate their IC_50_ values (Table 3 and Figure 3).

**Table 3.** The IC_50_ values for compounds (**6c**, **12 b**, **12c**, 1**3c**).

Compound ID	VEGFR-2 IC_50_
**6c**	13.4 μM
**12b**	4.4 μM
**12c**	2.7 μM
**13c**	2.5 μM

Results confirmed the significant inhibition of 4-chlorophthalazines bearing a biarylurea tail with a terminal 4-chloro substitution **(12b)** or a terminal 4-chloro-3-CF_3_ substitution **(12c, 13c)** with IC_50_s of 4.4, 2.7 and 2.5 µM respectively.

#### 4.1.4. *In vitro* HUVEC anti-proliferative assay

In order to further assess the antiangiogenic activity of the promising candidates, three compounds **(12b, 12c and 13c)** showing best VEGFR-2 IC_50_s were selected to be tested for their ability to *in vitro* inhibit VEGF-induced HUVEC cell line proliferation at single dose of 10 µM concentration.

The HUVEC anti-proliferative assay was also carried out in BPS Bioscience Corporation, San Diego, CA, USA (www.bpsbioscience.com).

Angiogenesis process comprises endothelial cell (EC) outgrowth from the parent vessel, followed by proliferation, migration, tube formation, alignment, and anastomosis to other vessels. Several *in vitro* models have attempted to recreate this complex progression of events^22^. The development of atherosclerotic plaques and angiogenesis, the role of the endothelium in the response of the blood vessel wall to shear forces and stretch in addition to creating a model system for the study of the regulation of endothelial cell function had emphasised the importance of using human umbilical vein endothelial cells (HUVECs). As most endothelial cell assays utilise human umbilical vein endothelial cells (HUVECs) or bovine aortic endothelial cells (BAECs) as being relatively easy to harvest from large blood vessels and also as representatives of vascular endothelial cells *in vivo* ^23^.

**Figure 4.**
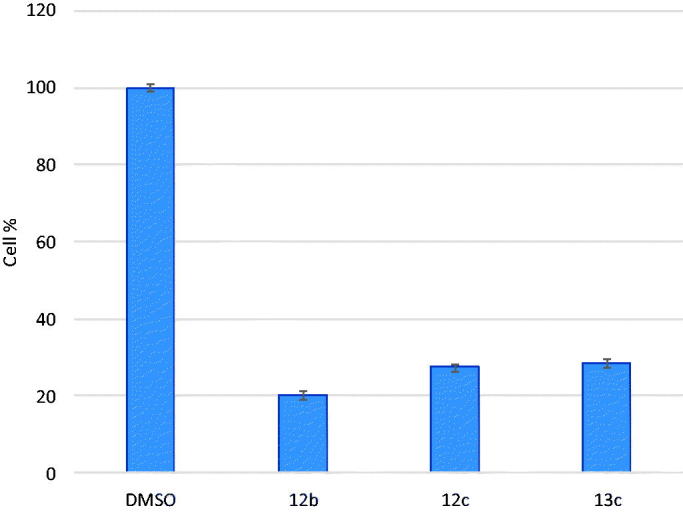
The bar graphs showing the HUVECs growth percentage after treatment with the target compounds.

The test compounds **(12b, 12c and 13c)** manifested significant inhibition of HUVEC cell lines of 79.83%, 72.58% and 71.60%, respectively, which may support their potential antiangiogenic activity (Table 4 and Figure 4).

**Table 4.** The effect of Compounds (**12 b**, **12c**, **13c**) on HUVEC proliferation.

Compound ID	% Cell growth	% Cell inhibition
**12b**	20.17	79.83
**12c**	27.42	72.58
**13c**	28.40	71.60

#### 4.1.5. Cell-cycle analysis

In an attempt to elucidate the potential mechanism of action of the synthesised phthalazines that exhibited potent anti-proliferative activity against the NCI cell panel but weak or moderate VEGFR-2 inhibitory activity, compounds **(7b and 16a)** were selected to investigate their effect on cell-cycle progression, and induction of apoptosis using breast cancer adenocarcinoma cell line (MCF-7) and colon cancer cell line (HCT-116), respectively.

From another point of view, since the inhibition of VEGF pathway has a greater effect on the induction of endothelial cell apoptosis and the decrease of vascular tubulogenesis, as the repair of endothelial lining defects caused by endothelial apoptosis is coordinated by growth factors, mainly by vascular endothelial growth factor (VEGF), which conveys survival signals to endothelial cells and acts as a potent antiapoptotic, proliferation stimulating factor^26^; thus, the biarylurea derivative (**13c**) (VEGFR-2 IC_50_ = 2.5 µM) was selected to test its effect on cell cycle analysis and induction of apoptosis using breast cancer adenocarcinoma cell line (MCF-7).

Cell cycle is a chain of events that takes place in a cell leading to its division and replication. G1 phase, S phase (synthesis), G2 phase (collectively known as interphase) and M phase (mitosis) are considered as the four main distinct phases of the cell cycle. The G1-phase, also called post-mitotic pre-synthesis phase, directly follows cell division. The S- or synthesis phase is characterised by the process of DNA replication. The G2-, premitotic or post-synthetic phase is the time when the cell prepares to split off in two cells, the actual division. Finally, in the M- or mitosis-phase division, the doubled DNA organised in chromosomes is separated^27^.

**Figure 5.**
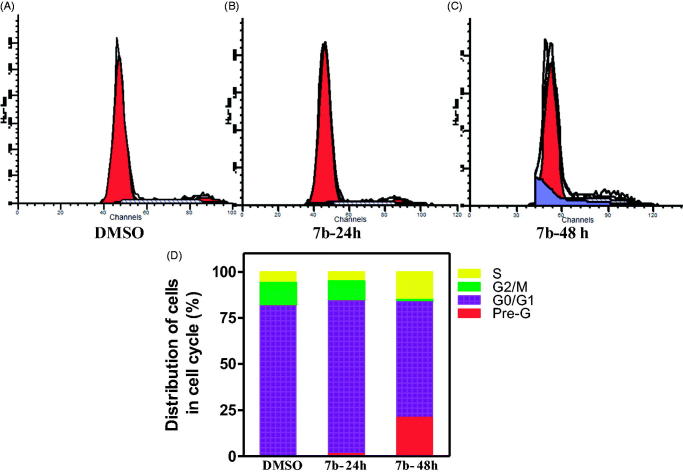
Effect of compound 7b on DNA-ploidy flow cytometric analysis of MCF7 cells. MCF-7cells were treated with DMSO 0.01% or compound **7b** at its **GI_50_** (0.32 µM), for 24 h and 48 h, and the harvested cells were subjected to Cell-cycle analysis using a FACS Calibur flow cytometer (A,B and C). Bar chart shows percentage of MCF7 cells at each phase of cell cycle in DMSO and compound 7b treated cells.

**Figure 6.**
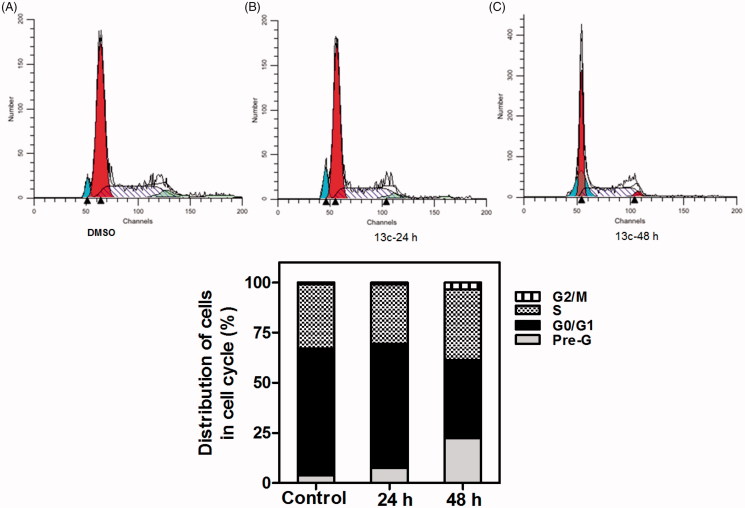
Effect of compound 13c on DNA-ploidy flow cytometric analysis of MCF7 cells. MCF-7cells were treated with DMSO 0.01% or compound **13c** at its **GI_50_** (0.57 µM), for 24 h and 48 h, and the harvested cells were subjected to cell-cycle analysis using a FACS Calibur flow cytometer (A, B and C). Bar chart shows percentage of MCF7 cells at each phase of cell cycle in DMSO and compound **13c** treated cells.

The effect of compound (**7b and 13c)** on the normal cell-cycle progression was characterised using flow cytometric analysis of the DNA ploidy in MCF-7 cells using BD FACSDIVA ^TM^ software. Exposure of MCF-7 cells to either **7b or 13c** at their **GI_50_** concentration (0.32 µM, 0.57 µM, respectively) for 24 h and 48 h was shown to induce a remarkable disruption in cell cycle profile and cell-cycle arrest at S-phase boundary with concurrent time-dependent increase in pre-G cell population (Figures 5 and 6)**. 13c** showed excellent disruption and even more prominent increase in sub-G1 cell population than **7b.** The observed increase in sub-G1 cell population may imply DNA fragmentation and apoptosis as a potential mechanism for **7b/13c**-induced cancer cell death.

**Figure 7.**
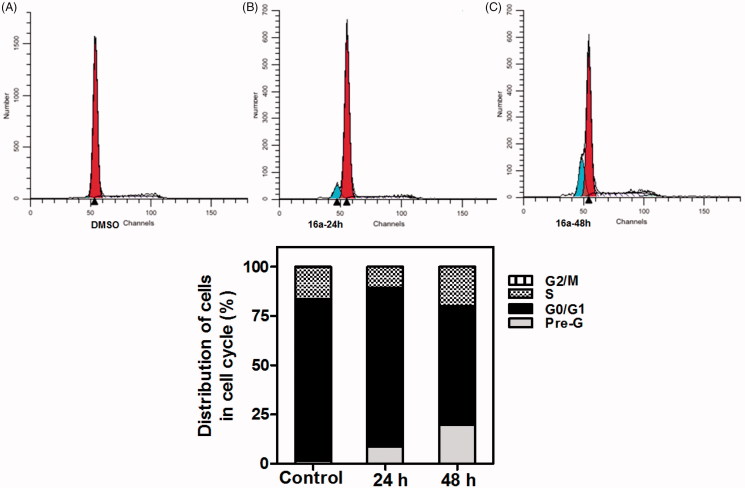
Effect of compound 16a on DNA-ploidy flow cytometric analysis of HCT-116 cells. HCT-116 cells were treated with DMSO 0.01% or compound **16a** at its **GI_50_** (1.20 µM), for 24 h and 48 h, and the harvested cells were subjected to Cell-cycle analysis using a FACS Calibur flow cytometer (A,B and C). Bar chart shows percentage of HCT-116 cells at each phase of cell cycle in DMSO and compound **16a** treated cells.

Furthermore, exposure of HCT-116 cells to **16a** at GI_50_ concentration (1.20 µM) for 24 h and 48 h also seemed to induce a significant action at both S phase and sub-G1 cell population (Figure 7).

#### 4.1.6. Apoptosis determination

**Figure 8.**
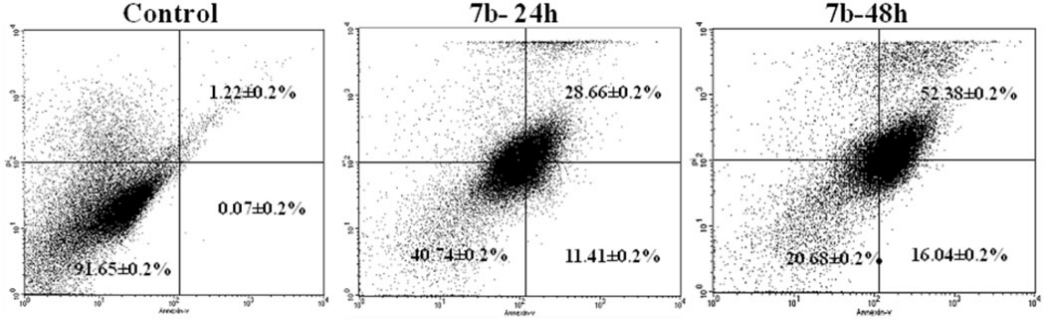
Effect of compound 7b treatment on induction of apoptosis. MCF-7 cells were treated with DMSO 0.01% or compound **7b** at its **GI_50_** (0.32 µM) for 24 or 48h, the harvested cells were stained with Annexin V-FITC apoptosis detection kit and Analyzed on a Flow cytometer.

**Figure 9.**
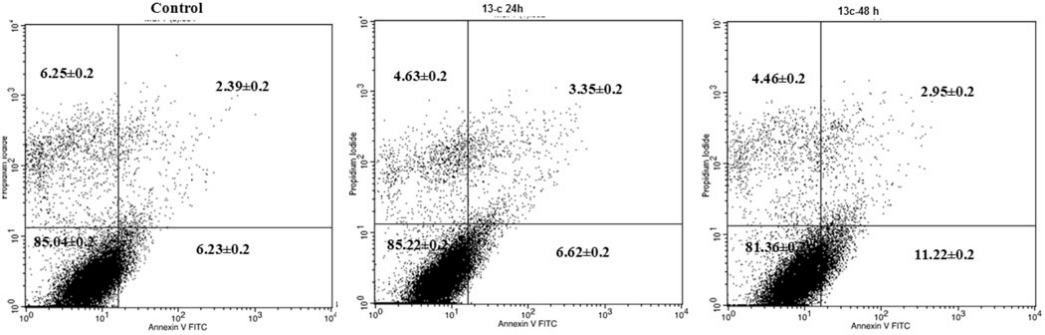
Effect of compound 13c treatment on induction of apoptosis. MCF-7 cells were treated with DMSO 0.01% or compound **13c** at its **GI_50_**(0.57 µM) for 24 or 48h, the harvested cells were stained with Annexin V-FITC apoptosis detection kit and Analyzed on a Flow cytometer.

**Figure 10.**
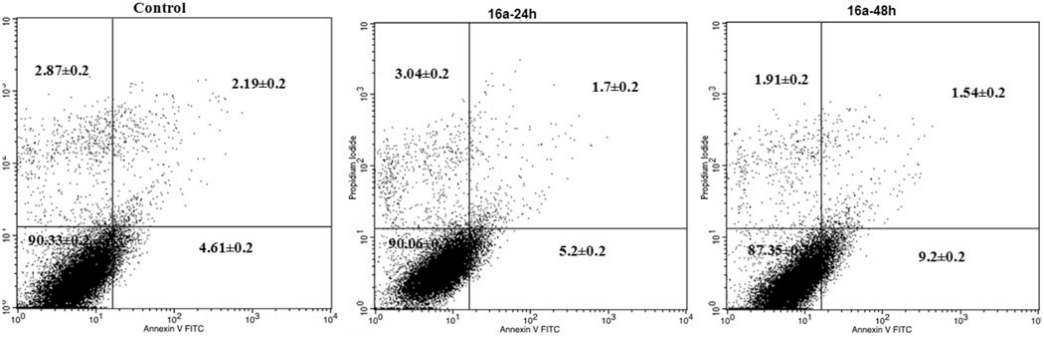
Effect of compound 16a treatment on induction of apoptosis. HCT-116 cells were treated with DMSO 0.01% or compound **16a** at its **GI_50_**(1.20 µM) for 24 or 48h, the harvested cells were stained with Annexin V-FITC apoptosis detection kit and Analyzed on a Flow cytometer.

To further investigate the ability of compounds **(7b, 13c and 16a)** to induce apoptosis, an Annexin V (conjugated to FITC) apoptosis detection kit was employed. This assay detects phosphatidylserine (PS) expressed on the surface of apoptotic cells and fluoresces green after interacting with the labelled Annexin V. During early apoptosis, membrane asymmetry is lost, and PS translocates from the cytoplasmic side of the membrane to the external leaflet. Propidium iodide (PI), the counter stain used in this assay, has the ability to cross only compromised membranes to intercalate into the DNA. Therefore, PI is used to detect the late stages of apoptosis by the presence of red fluorescence. Exposure of MCF-7 cells to **7b** at its GI_50_ (0.32 µM) for 24 h and 48 h increased the percentage of Annexin-V positive cells indicating an early (lower right quadrant) or late (upper right quadrant) apoptosis in a time-dependent manner compared to DMSO treated cells (Figure 8). However, **13c** and **16a** increased the percentage of Annexin-V positive cells in lower right quadrant indicating early apoptosis (Figures 9 and 10).

#### 4.1.7. Effects of 7b on the cellular and nuclear morphology

**Figure 11.**
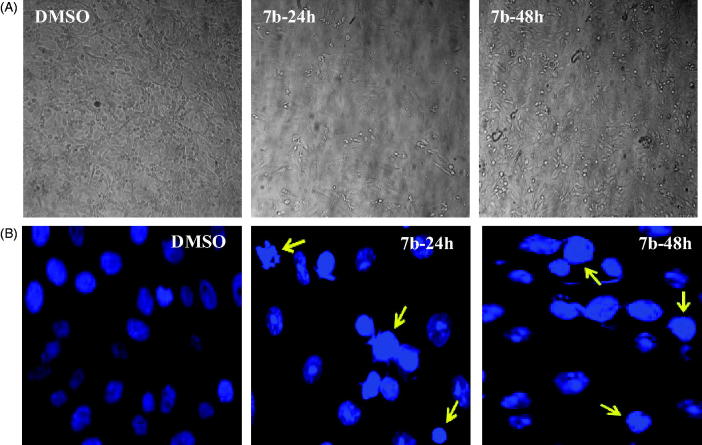
Effect of compound 7b on the cellular and nuclear morphology. MCF-7 cells were seeded on cover slips in a 6- well tissue culture plate, the cells then were treated with DMSO 0.01% or compound **7b** (0.32 µM) for 24 or 48h, the cells were imaged with the light microscope to examine the cellular morphology (A) or stained with the nuclear stain Dapi to study the nuclear morphology using fluorescence microscope (B).

To further examine the apoptosis inducing effect of **7b**, the cellular as well as nuclear morphological changes for MCF-7 cells treated with **7b** (0.32 µM) were studied following Dapi staining. The effect of **7b** on cell morphology was initially analysed with light microscopy. While control cells treated with DMSO exhibited normal morphological features, cells treated with **7b** for 24 and 48 h showed deteriorated morphological changes including cell rounding, detachment and cellular fragmentation (Figure 11(A)). Similarly, fluorescence staining further indicated that, while DMSO-treated control cells exhibited uniformly dispersed chromatin, **7b**-treated cells showed typical apoptotic characteristics; including condensation of chromatin (brightly stained cells), blubbing with the appearance of nuclear fragmentation (arrow heads indicate apoptotic nucleus) (Figure 11(B)). These results collectively confirms the ability of compound (**7b)** to induce cellular apoptosis in MCF-7 cells as a mechanism of cancer cell death.

#### 4.1.8. Effect of compound 7b treatment on the expression level of cleaved caspase-3

**Figure 12.**
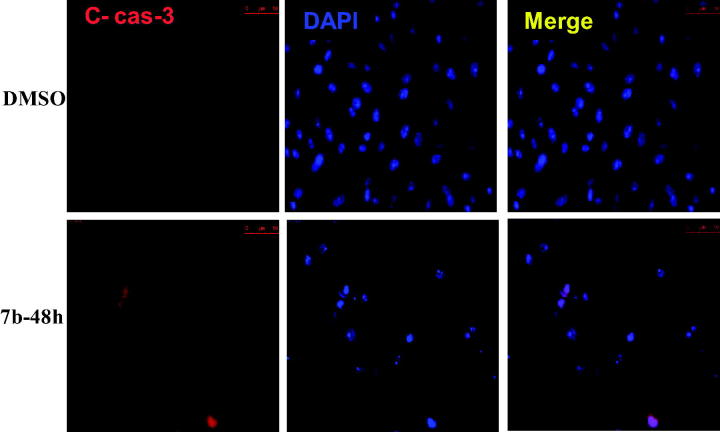
Effect of compound 7 b on cleaved caspase-3 protein expression. MCF-7 cells were seeded on cover slips in a 6-well tissue culture plate, the cells then were treated with DMSO 0.01% or compound **7b** at its **GI_50_**(0.32 µM) for 48h, the cells were then permiabilised, fixed, blocked and stained with the primary and secondary antibodies, the nuclear stain Dapi was used as a counter stain. The image were taken using a fluorescence microscope.

Caspase-3 is a member of the cysteine-aspartic acid protease (caspase) family. Sequential activation of caspases is considered as one of the important keys in cellular apoptosis process. Actually, caspases exists as inactive proenzymes that changed to the active form by undergoing proteolytic processing. Cleaved caspase-3 further activates a cascade of caspases that induce cell death. Thus, the ability of compound **7b** to increase the expression of cleaved caspase-3 was studied as a driving force in **7b**-induced apoptotic cell death. Exposure of MCF-7 cells to compound (**7b)** (0.32 µM) for 24 h and 48 h resulted in time-dependent increase in cleaved caspase-3 protein expression, indicating a pivotal involvement of caspases in the induced cancer cell death (Figure 12).

### 4.2. Molecular modelling studies

Molecular docking study was performed using AutoDock Vina^28^ through docking of the synthesised compounds in the VEGFR-2 kinase active site. Docking study aimed to interpret the VEGFR-2 inhibitory activity of the investigated molecules and gain further insight into their relative binding affinities and binding interactions with the kinase active site. The coordinates of the VEGFR-2 structure were obtained from the crystal structure of VEGFR complexed with sorafenib as its inhibitor (PDB code 4ASD), which revealed the hydrogen bond interactions between the NH and CO motifs of urea moiety with the backbone of Asp1046 and the carboxylic acid moiety of Glu885, respectively, as well as a H-bond with Cys919 residue in the hinge region of the kinase active site^20^.

Owing to the fact that the chloro group of the co-crystallised ligand (sorafenib) is in the far proximity to any halogen-bond^29–31^ acceptor in the binding site (Figure S7 in Supplementary material), we excluded investigating halogen-bond contacts in our docking-based assessment.

**Figure 13.**
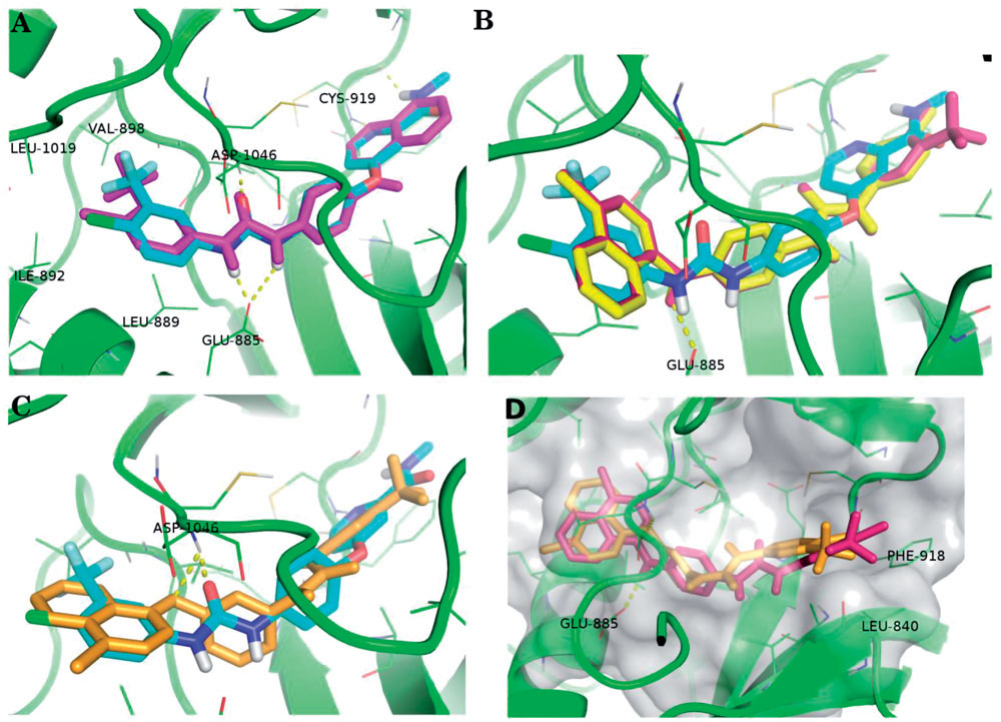
Overlay of the best-scored docking poses of some relevant compounds on the co-crystallised ligand Sorafenib (cyan sticks) in the binding site of VEGFR2 (PDB: 4ASD):. (A) The docking pose of **6c** as magenta sticks, (B) the docking poses of **12b** and **12c** as yellow and purple sticks, respectively, and (C) the docking pose of 13c as gold sticks. (D) The docking poses of **12c** and 13c at a different perspective of the binding site showing the extended hydrophobic contact of the chloro and trifluoromethyl phenyl moiety with the side chains of PHE-918 and LEU-840. The yellow dashed-lines represent the polar contacts (H-bonding interactions). Non-polar hydrogen atoms were omitted for clarity. All presented docking poses are the best-scored by AutoDock Vina.^28^

The best-scored pose of the biarylurea derivative (**6c**) reproduced the key interactions of the co-crystallised ligand (sorafenib) which includes H-bonding interactions of Glu885 and Asp1046 with the urea group of **6c**. In addition, other hydrophobic interactions can be observed for the hydrophobic side chains of Ile892, Leu1019 and Val898 with the halogen groups (chloro and trifluoromethyl) of the phenyl moiety of **6c**. Nevertheless, losing the H-bonding interaction of the Cys 919 with the terminal amide group of sorafenib can explain the lower activity of **6c** compared to sorafenib (Figure 13(A)).

The relative higher inhibitory activity of **6c** compared to similar analogues of **6a** and **6b** is most probably attributable to the fact that **6c** possessing additional halogen group (trifluoromethyl group) on the phenyl moiety which contributes to an additional hydrophobic interaction, this also agrees with the predicted score of the best poses by AutoDock Vina^28^ (Table 5). More information about rationalising the docking poses of the **6d, 6e and 6f** can be found in (Figure S8 in Supplementary material).

**Table 5.** The score of best-scored poses of some relevant compounds. The score is expressed as a binding affinity (kcal/mol) as an output of AutoDock Vina.^a^

Compound Number	Score (SD)	Average Number of Poses Per Run
**6a**	−11.4 (0)	4.6
**6b**	−11.5 (0)	4.6
**6c**	−12.4 (0.06)	5.3
**12a**	−11.0 (0)	4.3
**12b**	−11.1 (0.06)	5
**12c**	−11.5 (0)	8.3
**13a**	−10.7 (0)	9
**13b**	−10.8 (0)	9
**13c**	−11.0 (0)	9

^a^The shown score is the mean of three consecutive runs. The docking method was validated by successful pose-retrieval docking experiment of Sorafenib (score: −12.3(0)). SD is the standard deviation.

Unlike **6c**, the predicted best scored poses of both biarylurea derivatives **12b** and **12c** showed a flip of 180 degrees compared to sorafenib and biarylurea compound (**6c)** (Figure 13(B)). This implies that the chlorophthalazine moiety of both derivatives **12b** and **12c** is directed towards the 4-chloro-3-trifluoromethylphenyl moiety of sorafenib. Avoiding a possible clash of the chloro group with the backbone of Cys 919 can explain this flipping behaviour (Figure S9 in Supplementary material). The flipped pose showed interaction pattern in the binding site that maintained the H-bonding interaction of Glu885, however, in this case with the amino group of **12b** and **12c**. Also, chlorophthalazine ring was packed in the hydrophobic region of Ile892, Val898 and Leu1019. Similarly, the chlorophenyl moiety of **12b** and the 4-chloro-3-trifluoromethylphenyl moiety of **12c** showed hydrophobic contacts with the side chains of Phe918 and Leu840 (Figure 13(B,D)). Owing to the existence of additional hydrophobic contacts of **12c** (extra halogens) compared to **12a** and **12b**, the predicted scores showed relative improvement (Table 5). This also agrees with the marginal improvement of the observed inhibitory activity of **12c** compared to similar analogs. Additional Figures of the docked poses of **12a, 12b and 12c** can be found in the (Figure S10 in Supplementary material).

Like **12c**, the predicted pose of **13c** showed the same flip and relatively the same interaction pattern. Only exception is that the isosteric ether link in **13c** showed H-bonding interaction with Asp1046 instead of Glu885 with **12c** as shown in (Figure 13(C)). This agrees with the biological activity observed for both **12c** and **13c**.

## 5. Conclusion

Four series of phthalazine-based derivatives bearing biarylamides (**5a–d),** biarylurea (**6a–f, 7a–f, 12a–c and 13a–c)** or a substituted piperazine moiety at position 1 of the phathalazine nucleus (**8a–j),** in addition to two other series of vatalanib analogs **(16a–d, and 17a,b)** were designed, synthesised as anticancer agents exerting potent anti-proliferative activity as well as apoptosis inducers in tumour cells. Eight of the phthalazines derivatives; namely the biarylureas (**6b, 6e, 7b, 13a and 13c**) and the vatalanib analogues (**16a, 16d and 17a)** exhibited excellent broad spectrum anti-proliferative activity in NCI 5-log dose screening against 60 cell panel with GI_50_ values ranging from 0.15 to 8.41 µM, especially on leukaemia, renal, melanoma and breast cancer cell lines. Accordingly, the three compounds **(7b, 13c and 16a)** were selected to further investigate the potential underlying mechanisms that induced cancer cell death. The latter three compounds were found to induce cell-cycle arrest with concurrent increase in pre-G cell population and increase the apoptotic cell population in time-dependent manner. Moreover, compound (**7b**) was found to disrupt the normal cellular and nuclear morphology and increase the expression of cleaved caspase-3, the pro-apoptotic protein, in MCF-7 cell lines which collectively indicate the involvement of these compounds in apoptotic-induced cell death. Moreover, all the synthesised phthalazines were evaluated for their VEGFR-2 inhibitory activity, which revealed the significant inhibition of the 4-chlorophthalazines having a biarylurea tail with either a terminal 4-chloro **(12b)** or a terminal 4-chloro-3-trifhuoromethyl substituents **(12c and 13c)** with IC_50_ of 4.4, 2.7 and 2.5 µM, respectively. 4-chloropthalazines were found to exhibit better VEGFR-2 activity than their corresponding 4-methyl or 4-unsubstituted analogues. **12b, 12c and 13c** also displayed significant inhibition of VEGF-stimulated proliferation of human umbilical vein endothelial cells (HUVEC) with 79.83, 72.58 and 71.6% inhibition, respectively, at 10 µM concentration. Finally, molecular docking study of the active biarylurea compounds **(6c, 12b, 12c and 13c)** in VEGFR-2 kinase active site revealed their ability to form the essential H bond interactions with Glu 885 or Asp1046 key residues in the VEGFR-2 active site.

## 6. Experimental

### 6.1. Chemistry

Starting materials and reagents were purchased from Sigma – Aldrich or Acros Organics. Melting points were recorded on Gallen Kamp apparatus and were uncorrected. FT-IR spectra were recorded on a Shimadzu IR 435 spectrophotometer. ^1^HNMR spectra were recorded in δ scale given in ppm on a Varian 400 MHz spectrophotometer or a Varian 300 MHz spectrophotometer. Coupling patterns are described as follows: *s*, singlet; *d*, doublet, *dd*, doubled doublet; t, triplet; m, multiplet. *J* describes a coupling constant. The coupling constants were rounded off to one decimal place. MS spectra mass were recorded on Hewlett Packard 5988 spectrometer (70 eV). Elemental analyses were performed at the Microanalytical Center, Al-Azhar University. Compounds **1a–c** ^21^, **2a,b** ^32^ **and 3a,b** ^33^, **9** and **10** ^34^ were prepared following reported procedures.

#### 6.1.1. Synthesis of N^1^-(4-substituted-phthalazin-1-yl)benzene-1,4-diamine (4a,b)

General procedure:

*p*-Phenylenediamine (0.33 g, 3.12 mmol, 3equiv) and the respective chlorophthalazine (2.08 mmol, 2equiv) were treated with 2-BuOH (7.5 ml) in a tube and heated to 110 °C. The reaction quickly became a solid, yellow mass. After four hours, the reaction was cooled and diluted with water. The resultant slurry was then partitioned between equal volumes of DCM and 1 N NaOH (15 ml). The aqueous layer was extracted into DCM (2x15 ml). The combined organic layers were dried over anhydrous sodium sulfate, filtered, and concentrated *in vacuo*. The resulting orange solid was crystallised from (EtOAc/pet ether 1:1) to afford the title compounds **(4a,b)** as an orange crystals.

##### 6.1.1.1. N^1^-(Phthalazin-1-yl)benzene-1,4-diamine (4a)

Yield (0.21 g, 91%); mp 284–286 °C; **^1^HNMR (300 MHz**, **DMSO-d_6_**): δ 9.15 (1H, *s*, –NH D_2_O exchangeable), 9.04 (2H, *s*, –NH_2_ D_2_O exchangeable), δ 8.95–8.92 (2H, d, *J* = 9 Hz, phthalazine), δ 8.18–8.15 (2H,d, *J* = 9 Hz, phthalazine), δ 7.97 (1H, s, phthalazine), δ 7.65–7.62 (2H,d, *J* = 9 Hz, ArH), δ 6.97–6.83 (2H,d, *J* = 9 Hz, ArH); FT-IR (ύ max, cm^−1^): 3400 (NH_2_), 3329 (NH); **MS** (Mwt.: 236.11): *m/z* 236.00 (M+, 43.00%), 212.00 (100.00%), 118.00 (54.00%), 56.00 (70.00%); Anal. Calcd for C_14_H_12_N_4_: C, 71.17; H, 5.12; N, 23.71; Found: C, 71.34; H, 5.17; N, 23.96

##### 6.1.1.2. N^1^-(4-Methylphthalazin-1-yl)benzene-1,4-diamine (4b)

Yield (0.22 g, 88%); mp 240–242 °C; **^1^HNMR (300 MHz**, **DMSO-d_6_**): δ 8.88 (1H, *s*, –NH D_2_O exchangeable), 8.85 (2H, *s*, –NH_2_ D_2_O exchangeable), δ 8.13–8.08 (2H, d, *J* = 7 Hz, phthalazine), δ 7.95–7.92 (2H, d, *J* = 7 Hz, phthalazine), δ 7.46–7.43 (2H, d, *J* = 9 Hz, ArH), δ 6.83–6.76 (2H, d, *J* = 9 Hz, ArH), δ 2.78 (3H, s, CH_3_); **^13^CNMR (100 MHz, DMSO)** *:* δ 162.4, 150.5, 135.2, 134.5, 131.2, 130.7, 128.0, 127.3, 125.0, 122.8, 120.7, 120.2, 115.6, 115.3, 18.2; FT-IR (ύ max, cm^−1^): 3385 (NH_2_), 3321 (NH); **MS** (Mwt.: 250.12): *m*/*z* 250.00 (M+, 50.00%), 249.00 (100.00%), 108.00 (60.92%), 65.00 (77.59%); Anal. Calcd for C_15_H_14_N_4_: C, 71.98; H, 5.64; N, 22.38; Found: C, 72.09; H, 5.78; N, 22.52.

#### 6.1.2. Synthesis of N-(4–(4-Substitutedphthalazin-1-ylamino)phenyl)-4-substituted-benzamides (5a-d)

General procedure:

To a stirred solution of the respective N1-(4-arylphthalazin-1-yl)benzene-1,4-diamine **(4a,b)** (10.0 mmol, 1equiv) and triethylamine (1.1 ml, 10.0 mmol) in acetonitrile (20 ml), the respective benzoyl chloride (viz.; benzoyl chloride, 4-chlorobenzoyl chloride) (10.0 mmol, 1equiv) was added and the mixture was heated under reflux for 6 h, until the disappearance of starting material as judged by TLC (CHCl_3_/CH_3_OH 9.5:0.5). The reaction mixture was filtered and then the filtrate was concentrated *in vacuo*, to afford the crude product which was purified by flash column chromatography (EtOAc/pet ether 9:1) to afford **5a–d** as yellow crystals.

##### 6.1.2.1. N-(4-(Phthalazin-1-ylamino)phenyl)benzamide (5a)

Yield (0.17 g; 50%), mp 142–144 °C; **^1^HNMR (400 MHz, DMSO-d_6_)**: δ 9.66 (1H, *s*, –NH D_2_O exchangeable), δ 9.12 (1H, *s*, –NH D_2_O exchangeable), δ 8.62 (1H, s, *J* = 8 Hz, phthalazine), δ 8.11–8.08 (2H, d, *J* = 7.6 Hz, Ar-H), δ 7.98–7.96 (2H, d, *J* = 8 Hz, phthalazine), δ 7.93–7.88 (2H, *m*, phthalazine), δ 7.64–7.62 (1H, *m*, Ar-H), δ 7.62–7.60 (2H, d, *J* = 7.6 Hz, Ar-H), δ 7.59–7.57 (2H, d, *J* = 6.8 Hz, Ar-H), δ 7.50–7.48 (2H, *m*, Ar-H); FT-IR (ύ max, cm^−1^): 3550, 3510 (2NH), 1649 (C=O); **MS** (Mwt.: 340.38): *m*/*z* 340.15 [M**^+^**, 1.58%), 138.05 (2.70%), 86.10 (100%); Anal. Calcd for C_21_H_16_N_4_O: C, 74.10; H, 4.74; N, 16.46; Found: C, 74.34; H, 4.80; N, 16.58.

##### 6.1.2.2. 4-Chloro-N-(4-(phthalazin-1-ylamino)phenyl)benzamide (5b)

Yield (0.20 g; 54%), mp 156–158 °C; **^1^HNMR (400 MHz, DMSO-d_6_)**: δ 10.50 (1H, *s*, –NH D_2_O exchangeable), δ 9.15 (1H, *s*, –NH D_2_O exchangeable), δ 8.99 (1H, *s*, phthalazine), δ 8.11–8.08 (2H, d, *J* = 9 Hz, Ar-H), δ 7.94–7.92 (2H,d, *J* = 7.6 Hz, phthalazine), δ 7.91–7.90 (2H, d, *J* = 7.6 Hz phthalazine), δ 7.56–7.54 (2H,d, *J* = 9 Hz, Ar-H), δ 7.53–7.51 (2H,d, *J* = 7.5 Hz, Ar-H), δ 7.31–7.28 (2H, m, Ar-H); FT-IR (ύ max, cm^−1^): 3355, 3310 (2NH), 1640 (C=O); **MS** (Mwt.: 374.82): *m/z* 375.00 (M+, 1.57%), 245.00 (2.41%), 139.05 (46.44%), 111.10 (25.30), 64.00 (100.00%); Anal. Calcd for C_21_H_15_ClN_4_O: C, 67.29; H, 4.03; N, 14.95; Found: C, 67.52; H, 4.08; N, 15.22.

##### 6.1.2.3. N-(4–(4-Methylphthalazin-1-ylamino)phenyl)benzamide(5c)

Yield (0.24 g; 68%), mp 283–285 °C; **^1^HNMR (400 MHz, DMSO-d_6_)**: δ 10.50 (1H, *s*, –NH D_2_O exchangeable), δ 10.40 (1H, *s*, –NH D_2_O exchangeable), δ 8.27–8.24 (2H, d, *J* = 7.6 Hz, phthalazine), δ 8.02–8.00 (2H, m, Ar-H), δ 7.99–7.97 (2H,d, *J* = 7.6 Hz, phthalazine), δ 7.77–7.73 (1H, d, *J* = 9.2 Hz, Ar-H), δ 7.63–7.61 (2H,d, *J* = 7.2 Hz, Ar-H), δ 7.59–7.56 (2H,d, *J* = 7.6 Hz, Ar-H), δ 7.54–7.51 (2H, m, Ar-H), δ 2.91 (3H,s, CH_3_); FT-IR (ύ max, cm^−1^): 3331, 3257 (2NH), 1649 (C=O); **MS** (Mwt.: 354.15): *m/z* 354.95 (M+, 1.18%), 315.95 (15.90%), 105.00 (100.00%); Anal. Calcd for C_22_H_18_N_4_O: C, 74.56; H, 5.12; N, 15.81; Found: C, 74.78; H, 5.19; N, 16.04

##### 6.1.2.4. 4-Chloro-N-(4–(4-methylphthalazin-1-ylamino)phenyl)benzamide (5d)

Yield (0.20 g; 51%), mp 147–149 °C; **^1^HNMR (400 MHz, DMSO-d_6_)**: δ 10.65 (1H, *s*, –NH D_2_O exchangeable), δ 10.45 (1H, *s*, –NH D_2_O exchangeable), δ 8.05–8.03 (2H, d, *J* = 8 Hz, phthalazine), δ 7.97–7.95 (2H,d, *J* = 7.6 Hz, Ar-H), δ 7.88–7.86 (2H, d, *J* = 8 Hz, phthalazine), δ 7.63–7.61 (2H, d, *J* = 7.6 Hz, Ar-H), δ 7.59–7.57 (2H,d, *J* = 7.6 Hz, Ar-H), δ 7.31–7.28 (2H, *m*, Ar-H), δ 2.91 (3H, s,CH_3_); **^13^CNMR (100 MHz, DMSO)** *:* δ 166.9, 165.0, 151.5, 138.45, 138.2, 137.5, 137.0, 136.9, 134.6, 133.9, 131.6, 130.9, 130.2, 130.2, 130.1, 129.2, 128.9, 128.1, 125.3, 121.5, 121.2, 18.9; FT-IR (ύ max, cm-^1^): 3300, 3192 (2NH), 1678 (C=O); **MS** (Mwt.: 388.85): *m*/*z* 388.90 (M+, 7.10%), 279.90 (7.84%), 248.95 (25.65%), 139.00 (100%); Anal. Calcd for C_22_H_17_ClN_4_O: C, 67.95; H, 4.41; N, 14.41; Found: C, 68.24; H, 4.48; N, 14.49.

#### 6.1.3. Synthesis of 1-Aryl-3–(4-(4-substitutedphthalazin-1-ylamino)phenyl)ureas (6a-f)

General procedure:

To a stirred solution of the respective N1-(4-arylphthalazin-1-yl)benzene-1,4-diamine **(4a,b)** (10.0 mmol, 1equiv) in DMF (20 ml), the respective phenylisocyanate (viz.; phenyl- isocyanate, 4-chlorophenylisocyanate, 3-trifluoromethyl-4-chlorophenylisocyanate) (10.0 mmol, 1 equiv) was added and the mixture was heated under reflux for 6 h, after which TLC (CHCl_3_/CH_3_OH 9:1) showed no starting material. The reaction mixture was poured over ice water, the formed precipitate was allowed to settle, then filtered off and dried to afford the crude products**(6a-f)** which was further crystallised from EtOAc.

##### 6.1.3.1. 1-Phenyl-3–(4-(phthalazin-1-ylamino)phenyl)urea (6a)

Yield (0.10 g; 30%), mp 145– 147 °C; **^1^HNMR (400 MHz, DMSO-d_6_)**: δ 9.22 (1H, s,–NH D_2_O exchangeable), δ 9.16 (1H,s, –NH D_2_O exchangeable), δ 9.09 (1H, *s*, –NH D_2_O exchangeable), δ 8.74 (1H, *s*, phthalazine), δ 8.68–8.66 (2H, d, *J* = 8 Hz, phthalazine), δ 8.02–8.00 (2H, d, *J* = 8 Hz, phthalazine), δ 7.58–7.56 (2H, *m*, Ar-H), δ 7.48–7.46 (2H, d, *J* = 7.6 Hz, Ar-H), δ 7.31–7.29 (2H,d, *J* = 7.2 Hz, Ar-H), δ 7.19–7.17 (1H, *m*, Ar-H), δ 6.98–6.96 (2H, d, *J* = 7.2 Hz, Ar-H) FT-IR (ύ max, cm^−1^): 3300, 3047 (3NH), 1700 (C=O); **MS** (Mwt.: 355.14): *m/z* 355.10 (M+, 8.88%), 289.10 (9.51%), 123.10 (15.43%), 69.00(100.00%); Anal. Calcd for C_21_H_17_N_5_O: C, 70.97; H, 4.82; N, 19.71; Found: C, 71.21; H, 4.89; N, 19.88

##### 6.1.3.2. 1–(4-Chlorophenyl)-3–(4-(phthalazin-1-ylamino)phenyl)urea (6b)

Yield (0.20 g; 53%), mp 168–171 °C; **^1^HNMR (400 MHz, DMSO-d_6_)**: δ 10.33 (1H, *s*, –NH D_2_O exchangeable), δ 10.07 (1H, *s*, –NH D_2_O exchangeable), δ 9.16 (1H, *s*, –NH D_2_O exchangeable), δ 8.96 (1H, *s*, phthalazine), δ 8.09–8.07 (2H, d, *J* = 9.2 Hz, phthalazine), δ 7.63–7.61 (2H, d, *J* = 9.2 Hz, phthalazine), δ 7.49–7.47 (2H, d, *J* = 8.8 Hz, Ar-H), δ 7.44–7.42 (2H, d, *J* = 8 Hz, Ar-H), δ 7.33–7.31 (d, *J* = 8.8 Hz, 2H, Ar-H), δ 6.56–6.54 (2H,d, *J* = 8 Hz, Ar-H); **^13^CNMR (100 MHz, DMSO)** *:* δ 160.2, 153.0, 145.4, 139.4, 137.6, 135.8, 134.8, 133.2, 132.6, 129.7, 129.2, 128.9, 127.7, 122.6, 122.4, 121.8, 121.5, 120.3, 119.9, 118.6, 115.6; FT-IR (ύ max, cm−^1^): 3396, 3341 (3NH), 1672 (C=O); **MS** (Mwt.: 389.10): *m/z* 389.00 (M+, 1.51%), 281.95 (19.41%), 253.95(100.00%), 152.95 (13.00%); Anal. Calcd for C_21_H_16_ClN_5_O: C, 64.70; H, 4.14; N, 17.96; Found: C, 64.89; H, 4.21; N, 18.17.

##### 6.1.3.3. 1–(4-Chloro-3-(trifluoromethyl)phenyl)-3–(4-(phthalazin-1-ylamino)phenyl)urea (6c)

Yield (0.22 g; 49%), mp 288–291 °C; **^1^HNMR (400 MHz, DMSO-d_6_)**: δ 10.08 (1H, *s*, –NH D_2_O exchangeable), δ 9.36 (1H, *s*, –NH D_2_O exchangeable), δ 9.11 (1H, *s*, –NH D_2_O exchangeable), δ 8.22 (1H, *s*, Ar-H), 8.09–8.07 (2H, d, *J* = 8.8 Hz, phthalazine), δ 7.97–7.95 (2H, d, *J* = 8.8 Hz, phthalazine), δ 7.79 (1H, s, Ar-H), δ 7.69–7.67 (2H, d, *J* = 9.6 Hz, Ar-H), δ 7.52–7.50 (2H, d, *J* = 8.4 Hz, Ar-H), δ 7.39–7.37 (2H, d, *J* = 8.4 Hz, Ar-H); FT-IR (ύ max, cm^−1^): 3321, 3263, 3126 (3NH), 1649 (C=O); **MS** (Mwt.: 457.09): m/z 457.90 (M+, 1.06%), 415.90 (7.68%), 194.95(100.00%); Anal. Calcd for C_22_H_15_ClF_3_N_5_O: C, 57.71; H, 3.30; N, 15.30; Found: C, 57.93; H, 3.28; N, 15.48

##### 6.1.3.4. 1–(4-(4-Metyhlphthalazin-1-ylamino)phenyl)3-phenylurea(6d)

Yield (0.06 g; 20%), mp 284–286 °C; **^1^HNMR (300 MHz, DMSO-d_6_)**: δ 9.20(1H, *s*, –NH D_2_O exchangeable), δ 9.14 (1H, *s*,–NH D_2_O exchangeable), δ 9.06 (1H, *s*,–NH D_2_O exchangeable), δ 8.93–8.91 (1H, d, *J* = 7 Hz, phthalazine), δ 8.77–8.75 (1H, d, *J* = 7 Hz, phthalazine), δ 8.22–8.20 (2H, d, *J* = 7 Hz, phthalazine), δ 7.85–7.83 (2H, d, *J* = 8.4 Hz, Ar-H), δ 7.52–7.49 (2H, d, *J* = 8.4 Hz, Ar-H), δ 7.43–7.40 (2H, d, *J* = 7.2 Hz, Ar-H), δ 7.25–7.23 (1H, m, Ar-H), δ 6.95–6.93 (2H, d, *J* = 7.2 Hz, Ar-H), δ 2.81 (3H, s, CH_3_);) ; FT-IR (ύ max, cm^−1^): 3292, 3194, 3132 (3NH), 1651 (C=O); **MS** (Mwt.: 369.16): *m*/*z* 369.00 (M+, 2.99%), 212.00 (6.90%), 93.00 (100.00%);Anal. Calcd for C_22_H_19_N_5_O: C, 71.53; H, 5.18; N, 18.96; Found: C, 71.69; H, 5.27; N, 19.21

##### 6.1.3.5. 1–(4-Chlorophenyl)-3–(4-(4-methylphthalazin-1-ylamino)phenyl)urea (6e)

Yield (0.20 g; 50%), mp 178–180 °C; **^1^HNMR (400 MHz, DMSO-d_6_)**: δ 9.20(1H, *s*, –NH D_2_O exchangeable), δ 9.17 (1H, *s*, –NH D_2_O exchangeable), δ 9.03 (1H, *s*, –NH D_2_O exchangeable), δ 8.18–8.16 (2H, d, *J* = 7.6 Hz, phthalazine), δ 8.09–8.05 (2H, d, *J* = 7.6 Hz, phthalazine), δ 7.86–7.84 (2H, d, *J* = 8.8 Hz, Ar-H), δ 7.73–7.71 (2H, d, *J* = 8.8 Hz, Ar-H), δ 7.49–7.47 (2H, d, *J* = 8.8 Hz, Ar-H), δ 7.33–7.30 (2H, d, *J* = 8.8 Hz, Ar-H), δ 2.80 (3H, s,CH_3_); FT-IR (ύ max, cm^−1^): 3294, 3188, 3168 (3NH), 1681 (C=O); **MS** (Mwt.: 403.86): *m/z* 403.00 (M+, 1.51%), 276.95 (17.01%), 127.00 (100.00%); Anal. Calcd for C_22_H_18_ClN_5_O: C, 65.43; H, 4.49; N, 17.34; Found: C, 65.62; H, 4.52; N, 17.52.

##### 6.1.3.6. 1–(4-Chloro-3-(trifluoromethyl)phenyl)-3–(4-(4-methylphthalazin-1-ylamino)phenyl)-urea (6f)

Yield (0.30 g; 63%), mp 258–260 °C; **^1^HNMR (400 MHz, DMSO-d_6_)**: δ 9.43(1H, *s*, –NH D_2_O exchangeable), δ 9.14 (1H, *s*, –NH D_2_O exchangeable), δ 9.02 (1H, *s*, –NH D_2_O exchangeable), δ 8.88–8.86 (2H, d, *J* = 8 Hz, phthalazine), δ 8.24–8.22 (2H, d, *J* = 8 Hz, phthalazine), δ 8.13 (1H, s,Ar-H), δ 7.97–7.95 (1H, d, *J* = 9.2 Hz, Ar-H), δ 7.88–7.86 (1H, d, *J* = 9.2 Hz, Ar-H), δ 7.63–7.61 (2H, d, *J* = 10 Hz, Ar-H), δ 6.78–6.76 (2H, d, *J* = 10 Hz, Ar-H), δ 2.80 (3H, s, CH_3_); **^13^CNMR (100 MHz, DMSO)** *:* δ 160.8, 153.0, 140.7, 139.4, 134.0, 132.7, 131.9, 127.7, 127.3, 127.1, 125.6, 124.8, 123.9, 122.1, 121.8, 121.4, 119.8, 119.5, 118.8, 118.6, 117.6, 115.4, 19.6; FT-IR (ύ max, cm^−1^): 3363, 3255, 3153 (3NH), 1681(C=O); **MS** (Mwt.: 471.86): *m/z* 472.10(M+, 0.30%), 223.00 (43.56%), 195.00 (70.63%), 52.05 (100%); Anal. Calcd for C_23_H_17_ClF_3_N_5_O: C, 58.54; H, 3.63; N, 14.84; Found: C, 58.61; H, 3.61; N, 15.03

#### 6.1.4. 1-Aryl-3–(4-(4-substituted-phthalazin-1-yloxy)phenyl)ureas (7a-f)

General procedure:

To a stirred solution of the respective chlorophthalazine derivative **(3a,b)** (10.0 mmol, 1equiv), and cesium carbonate (6.5 g, 20.0 mmol, 2equiv) in acetonitrile (20 ml), the appropriate 1-aryl-3–(4-hydroxyphenyl)urea **(1a-c)** (10.0 mmol, 1equiv) was added and the mixture was heated under reflux for 6 h, after which TLC (CHCl_3_/CH_3_OH 9:1) showed no starting material. The filtrate was evaporated *in-vacuo* to afford the crude product **(7a-f)** which was further purified by column chromatography (using gradient elution starting from EtOAc then 1% MeOH/EtOAc).

##### 6.1.4.1. 1-Phenyl-3–(4-(phthalazin-1-yloxy)phenyl)urea (7a)

Yield (0.22 g; 64%), mp 180–182 °C; **^1^HNMR(300 MHz, DMSO-d_6_)**: δ 9.57 (1H, *s,* –NH D_2_O exchangeable), δ 9.37 (1H, *s*, –NH D_2_O exchangeable), δ 8.36 (1H, *s*, phthalazine), δ 8.10–8.00 (2H, *m*, phthalazine), δ 7.93–7.91 (2H, d, *J* = 7 Hz, phthalazine), δ 7.91–7.90 (2H, *m*, Ar-H), δ 7.49–7.47 (2H, d, *J* = 7.8 Hz, Ar-H), δ 7.19–7.25 (*m*, 3H, Ar-H), δ 6.89–6.86 (1H, d, *J* = 7.5 Hz, Ar-H), δ 6.67–6.65 (1H, d, *J* = 7.8 Hz, Ar-H); FT-IR (ύ max, cm^−1^): 3304, 3235 (2NH), 1641 (C=O); **MS** (Mwt.: 356.38): m/z 355.00 (M-1^+^, 0.11%), 146.05 (23.71%), 118.05 (8.47%), 79.95 (100.00%); Anal. Calcd for C_21_H_16_N_4_O_2_: C, 70.77; H, 4.53; N, 15.72; Found: C,71.02; H, 4.61; N, 15.89

##### 6.1.4.2. 1–(4-Chlorophenyl)-3–(4-(phthalazin-1-yloxy)phenyl)urea (7b)

Yield (0.19 g; 50%), mp 166–168 °C; **^1^HNMR (400 MHz, DMSO-d_6_)**: δ 9.72 (1H, *s*, –NH D_2_O exchangeable), δ 9.44 (1H, *s*, –NH D_2_O exchangeable), δ 8.37 (1H, *s*, phthalazine), δ 8.28–8.26 (2H, d, *J* = 8 Hz, phthalazine), δ 8.18–8.16 (2H, d, *J* = 8 Hz, phthalazine), δ 7.58–7.56 (2H, d, *J* = 8.8 Hz, Ar-H), δ 7.56–7.53 (2H, d, *J* = 8.8 Hz, Ar-H), δ 7.36–7.34 (2H, d, *J* = 7.2 Hz, Ar-H), δ 7.14–7.12 (2H, d, *J* = 7.2 Hz, Ar-H); FT-IR (ύ max, cm^−1^): 3278, 3161 (2NH), 1701 (C=O); **MS** (Mwt.: 390.82): *m*/*z* 390.10 (4.47%), 365.10 (24.02%), 75.00 (100.00%);Anal. Calcd for C_21_H_15_ClN_4_O_2_: C, 64.54; H, 3.87; N, 14.34; Found: C, 64.69; H, 4.01; N, 14.62

##### 6.1.4.3. 1–(4-Chloro-3-(trifluoromethyl)phenyl)-3–(4-(phthalazin-1-yloxy)phenyl)urea (7c)

Yield (0.22 g; 49%), mp 158–160 °C; **^1^HNMR (400 MHz, DMSO-d_6_)**: δ 9.91 (1H, *s*, –NH D_2_O exchangeable), δ 9.08 (*s*, 1H, –NH D_2_O exchangeable), δ 8.39 (*s*, 1H, phthalazine), δ 8.24–8.21 (d, *J* = 8 Hz, 2H, phthalazine), δ 8.17–8.15 (d, *J* = 8 Hz, 2H, phthalazine), δ 7.99 (*s*, 1H, Ar-H), δ 7.85–7.83 (d, *J* = 7.6 Hz, 1H, Ar-H), δ 7.76–7.74 (d, *J* = 7.6 Hz, 1H, Ar-H), δ 7.28–7.26 (d, *J* = 8.4 Hz, 2H, Ar-H), δ 6.76–6.74 (d, *J* = 8.4 Hz, 1H, Ar-H); **^13^CNMR (100 MHz, DMSO)** *:* δ171.0, 153.7, 153.2, 141.1, 138.7, 133.8, 132.7, 132.0, 131.7, 129.5, 129.3, 129.1, 128.5, 127.0, 125.8, 124.7, 121.4, 121.2, 121.0, 119.6, 118.7, 115.6; FT-IR (ύ max, cm^−1^): 3300, 3153 (2NH), 1679 (C=O); **MS** (Mwt.: 458.82): *m/z* 458.10 (2.63%), 271.05 (7.73%), 195.00 (100.00%); Anal. Calcd for C_22_H_14_ClF_3_N_4_O_2_: C, 57.59; H, 3.08; N, 12.21; Found: C, 57.82; H, 3.06; N, 12.57

##### 6.1.4.4. 1–(4-(4-Methylphthalazin-1-yloxy)-3-phenylurea (7d)

Yield (0.20 g; 54%), mp 174–176 °C; **^1^HNMR (400 MHz, DMSO-d_6_)**: δ 8.76 (1H, *s*, –NH D_2_O exchangeable), δ 8.69 (1H, *s*, –NH D_2_O exchangeable), δ 8.39–8.37 (1H, d, *J* = 8 Hz, phthalazine), δ 8.23–8.21 (1H, d, *J* = 8 Hz, phthalazine), δ 8.11–8.09 (1H, d, *J* = 8 Hz, phthalazine), δ 8.08–8.06 (1H, d, *J* = 8 Hz, phthalazine), δ 7.55–7.52 (2H, d, *J* = 8.8 Hz, Ar-H), δ 7.46–7.43 (2H, d, *J* = 8.8 Hz, Ar-H), δ 7.33–7.30 (2H, d, *J* = 7.6 Hz, Ar-H), δ 7.22–7.20 (2H, d, *J* = 8.8 Hz, Ar-H), δ 6.69–6.67 (1H, d, *J* = 7.6 Hz, Ar-H), δ 2.96 (3H, s,CH_3_); FT-IR (ύ max, cm^−1^): 3302, 3141 (2NH), 1643 (C=O); **MS** (Mwt.: 370.40): *m/z* 370.00 [M**^+^**, 2.28%), 277.95 (19.33%), 251.00 (100.00%); Anal. Calcd for C_22_H_18_N_4_O_2_: C, 71.34; H, 4.90; N, 15.13; Found: C, 71.49; H, 4.97; N, 15.29.

##### 6.1.4.5. 1–(4-Chlorophenyl)-3–(4-(4-methylphthalazin-1-yloxy)phenyl)urea (7e)

Yield (0.17 g; 42%), mp 169–171 °C; **^1^HNMR (400 MHz, DMSO-d_6_)**: δ 10.06 (1H, *s*, –NH D_2_O exchangeable), δ 10.05 (1H, *s*, –NH D_2_O exchangeable), δ 8.38–8.36 (1H, d, *J* = 7.6 Hz, phthalazine), δ 8.20–8.18 (1H, d, *J* = 7.6 Hz, phthalazine), δ 8.07–8.04 (2H, d, *J* = 7.6 Hz, phthalazine), δ 7.58–7.56 (2H, d, *J* = 8.4 Hz, Ar-H), δ 7.29–7.22 (2H, d, *J* = 8.4 Hz, Ar-H), δ 7.20–7.18 (2H, d, *J* = 9.2 Hz, Ar-H), δ 6.66–6.63 (2H, d, *J* = 9.2 Hz, Ar-H), δ 2.81 (3H, *s*, CH_3_); FT-IR (ύ max, cm^−1^): 3300, 3153 (2NH), 1643 (C=O); **MS** (Mwt.: 404.85): *m/z* 404.90(M+, 2.04%), 385.85 (1.29%), 267.90 (4.75), 250.95 (28.16), 127.00 (100.00%); Anal. Calcd for C_22_H_17_ClN_4_O_2_: C, 65.27; H, 4.23; N, 13.84; Found: C, 65.52; H, 4.28; N, 13.97.

##### 6.1.4.6. 1–(4-Chloro-3-(trifluoromethyl)phenyl)-3–(4-(4-metyhlphthalazin-1-yloxy)phenyl)urea (7f)

Yield (0.20 g; 42%), mp 150–152 °C; **^1^H NMR (400 MHz, DMSO-d_6_)**: δ 10.31 (1H, s, –NH D_2_O exchangeable), δ 10.30 (1H, s, –NH D_2_O exchangeable), δ 8.37 (1H, *s*, Ar-H), δ 8.19–8.16 (2H, d, *J* = 8 Hz, phthalazine), δ 8.07–8.05 (1H, d, *J* = 7.6 Hz, Ar-H), δ 7.78–8.75 (1H,d, *J* = 8 Hz, phthalazine), δ 7.61–7.59 (2H, d, *J* = 7.6 Hz, Ar-H), δ 7.56–7.54 (1H, d, *J* = 8.8 Hz, Ar-H), δ 7.24–7.21(2H, d, *J* = 8.8 Hz, Ar-H), δ 2.80 (3H, *s*, CH_3_); FT-IR (ύ max, cm^−1^): 3300, 3153 (2NH), 1679 (C=O); **MS** (Mwt.: 472.85): *m/z* 472.80 (M+, 2.07%), 391.95 (10.86), 335.85 (25.53), 250.95 (86.76%), 194.90 (100%); Anal. Calcd for C_23_H_16_ClF_3_N_4_O_2_: C, 58.42; H, 3.41; N, 11.85; Found: C, 58.49; H, 3.39; N, 12.01

#### 6.1.5. Synthesis of ethyl 4–(4-substitutedphthalazine-1-yl)piperazine-1-carboxylate and 1-Substituted-4-(arylpiperazine-1-yl)phthalazine (8a–j)

General procedure:

To a stirred mixture of the respective 1-chlorophthalazine **(3a,b)** (10.0 mmol, 1equiv), potassium carbonate (0.27 g, 20.0 mmol, 2equiv), potassium iodide (0.01 g, 0.01 mmol, 0.1equiv) in absolute ethanol (20 ml), the respective piperazine (viz.; ethyl piperazinecarboxylate, phenylpiperazine, 2-flourophenylpiperazine, 2-pyridylpiperazine, 2-furoylpiperazine) (10.0 mmol, 1equiv) was added and the mixture was heated under reflux for 2 h, after which TLC (CHCl_3_/CH_3_OH 9:1) showed no starting material. The mixture was then concentrated *in vacuo*, the residue was washed with water, extracted with EtOAc, dried over anhydrous Na_2_SO_4_. The crude product was recrystallised from EtOAc.

##### 6.1.5.1. Ethyl 4-(phthalazin-1-yl)piperazine-1-carboxylate (8a)

Yield (0.19 g; 68%), mp 271–273 °C; **^1^HNMR (300 MHz, DMSO)**: δ 8.10 (1H, *s*, phthalazine), δ 7.98–7.96 (2H, d, *J* = 7 Hz, phthalazine), δ 7.93–7.91 (2H, d, *J* = 7 Hz, phthalazine), δ 4.07 (2H, q,CH_2_-CH_3_), δ 3.63–3.62 (2H, *m*, piperazine ring), δ 2.86–2.85 (2H, *m*, piperazine ring), δ 2.55–2.54 (2H, *m*, piperazine ring), δ 2.27–2.26 (2H, *m*, piperazine ring), δ 1.14 (3H, *t*, CH_2_-CH_3_); **^13^CNMR (100 MHz, DMSO)** *:* δ 179.9, 154.9, 149.2, 134.1, 132.3, 129.6, 127.2, 123.1, 122.7, 61.6, 43.1, 41.2, 30.1, 30.0, 14.6; FT-IR (ύ max, cm^−1^): 1672 (C=O); **MS** (Mwt.: 286.33): *m/z* 286.05 (M^+^, 0.95%), 270.00 (3.77%), 56.00 (100.00%); Anal. Calcd for C_15_H_18_N_4_O_2_: C, 62.92; H, 6.34; N, 19.57; Found: C, 63.23; H, 6.38; N, 19.71

##### 6.1.5.2. 1–(4-Phenylpiperazin-1-yl)phthalazine (8b)

Yield (0.20 g; 69%), mp 242–244 °C; **^1^HNMR (300 MHz, DMSO)**: δ 9.72–7.96 (1H, *s*, phthalazine), δ 8.17–8.15 (1H, d, *J* = 8 Hz, phthalazine), δ 8.12–8.10 (1H, d, *J* = 8 Hz, phthalazine), δ 8.00–7.95 (2H, d, *J* = 8 Hz, phthalazine), δ 7.28–7.24 (2H, d, *J* = 7.6 Hz, Ar-H), δ 7.05–7.03 (2H, d, *J* = 7.6 Hz, Ar-H), δ 6.84–6.81 (1H, d, *J* = 7.6 Hz, Ar-H), δ 3.58–3.56 (4H, *m*, piperazine ring), δ 3.44–3.42 (4H, *m*, piperazine ring); **^13^CNMR (100 MHz, DMSO)** *:* δ151.5, 148.2, 129.5, 128.6, 127.3, 124.3, 119.7, 116.0, 51.2, 48.7; FT-IR (ύ max, cm^−1^): 3428 (CH aromatic), 2991, 2919 (CH aliphatic); **MS** (Mwt.: 290.36): *m/z* 290.10 (M^+^, 0.41%), 162.10 (8.10%), 120.10 (100.00%), Anal. Calcd for C_18_H_18_N_4_: C, 74.46; H, 6.25; N, 19.30; Found: C, 74.62; H, 6.37; N, 19.54.

##### 6.1.5.3. 1–(4-(2-Fluorophenyl)piperazin-1-yl)phthalazine (8c)

Yield (0.21 g; 70%), mp 88–90 °C; **^1^HNMR (300 MHz, DMSO)**: δ 8.61 (1H, *s*, phthalazine), δ 8.27–8.24 (2H, d, *J* = 9 Hz, phthalazine), δ 8.14–8.11 (2H, d, *J* = 9 Hz, phthalazine), δ 7.19–7.17 (1H, d, *J* = 7.6 Hz, Ar-H), δ 7.15–7.13 (1H, d, *J* = 7.6 Hz, Ar-H), δ 7.08–7.06 (1H, d, *J* = 7.6 Hz, Ar-H), δ 6.99–6.97 (1H, d, *J* = 7.6 Hz, Ar-H), δ 3.70–3.69 (2H, m, piperazine ring), δ 3.31–3.30 (2H, m, piperazine ring), δ 3.23–3.21 (4H, m, piperazine ring); **^13^CNMR (100 MHz, DMSO)** *:* δ 175.9, 159.2,145.3, 138.9, 138.3, 134.8, 134.0, 130.0, 127.6, 125.6, 123.3, 122.8, 122.7, 116.0, 50.8, 49.9, 47.0, 46.9; FT-IR (ύ max, cm^−1^): 3039 (CH aromatic), 2924 (CH aliphatic); **MS** (Mwt.: 308.35): *m/* *z* 308.05 (M^+^, 1.81%), 180.05 (19.88%), 138.10 (100.00%); Anal. Calcd for C_18_H_17_FN_4_: C, 70.11; H, 5.56; N, 18.17; Found: C, 70.29; H, 5.60; N, 18.28.

##### 6.1.5.4. 1–(4-(Pyridin-2-yl)piperazin-1-yl)phthalazine (8d)

Yield (0.20 g; 69%), mp 286–288 °C; **^1^HNMR (300 MHz, DMSO)**: δ 8.14–8.12 (1H, d, *J* = 7 Hz, pyridine ring), δ 7.93–7.91 (3H, m, phthalazine), δ 7.59–7.56 (2H, d, *J* = 9 Hz, phthalazine), δ 7.56–7.54 (1H, d, *J* = 7 Hz, pyridine ring), δ 6.69–6.67 (2H, d, *J* = 7 Hz, pyridine ring), δ 3.67–3.65 (4H, m, piperazine ring), δ 3.06–3.04 (4H, m, piperazine ring); FT-IR (ύ max, cm^−1^): 3030 (CH aromatic), 2926 (CH aliphatic); **MS** (Mwt.: 291.35): *m/z* 291.10 (M+, 0.19%), 279.05 (0.28%), 160.10 (27.24%), 95.05 (100.00); Anal. Calcd for C_17_H_17_N_5_: C, 70.08; H, 5.88; N, 24.04; Found: C, 70.32; H, 5.64; N, 24.31

##### 6.1.5.5. Furan-2-yl-(4-(phthalazin-1-yl)piperazin-1-yl)methanone (8e)

Yield (0.23 g; 77%), mp 90–92 °C; **^1^HNMR (300 MHz, DMSO)**: δ 8.36 (1H, *s*, phthalazine), δ 8.12–8.10 (1H, d, *J* = 7 Hz, furoyl ring), δ 8.03–8.01 (2H, d, *J* = 7.8 Hz, phthalazine), δ 7.98–7.96 (2H, d, *J* = 7.8 Hz, phthalazine), δ 6.99–6.98 (2H, d, *J* = 7 Hz, furoylring), δ 3.69–3.67 (4H, m,piperazine ring), δ 2.91–2.90 (4H, *m*, piperazine ring); **^13^CNMR (100 MHz, DMSO)** *:* δ 175.9, 159.8, 154.6, 154.2, 142.3, 135.4, 133.9, 128.5, 127.8, 125.7, 123.2, 122.2, 117.2, 111.7, 50.2, 42.8, 40.2, FT-IR (ύ max, cm^−1^): 1679 (C=O); **MS** (Mwt.: 308.33): *m/z* 308.05 (M+, 4.64%), 274.00 (6.66%), 158.05 (57.70%), 95.00 (100.00%) Anal. Calcd for C_17_H_16_N_4_O_2_: C, 66.22; H, 5.23; N, 18.17; Found: C, 66.41; H, 5.32; N, 18.40

##### 6.1.5.6. Ethyl 4–(4-methylphthalazin-1-yl)piperazine-1-carboxylate (8f)

Yield (0.22 g; 73%), mp 95–97 °C; **^1^HNMR (300 MHz, CDCl_3_)**: δ 8.43–8.41 (2H, d, *J* = 8.1 Hz, phthalazine), δ 8.36–8.34 (2H, d, *J* = 8.1 Hz, phthalazine), δ 4.19 (2H, q, CH_2_-CH_3_), δ 3.75–3.74 (4H, m, piperazine ring), δ 3.65–3.64 (4H, *m*, piperazine ring), δ 3.37 (3H, *s*, CH_3_), δ 1.33 (3H, t,CH_3_-CH_2_); FT-IR (ύ max, cm^−1^): 1693 (C=O); **MS** (Mwt.: 300.36): *m*/*z* 300.05(M+, 5.71%), 244.05 (3.15%), 184.55 (13.85%), 172.30 (48.47%), 55.65 (100%); Anal. Calcd for C_16_H_20_N_4_O_2_: C, 63.98; H, 6.71; N, 18.65; Found: C, 64.17; H, 6.83; N, 19.01

##### 6.1.5.7. 1-Methyl-4–(4-phenylpiperazin-1-yl)phthalazine (8 g)

Yield (0.15 g; 56%), mp 229–231 °C; **^1^HNMR (300 MHz, DMSO)**: δ 7.28–7.26(2H, d, *J* = 7.2 Hz, phthalazine), δ 7.25–7.23 (2H, d, *J* = 7.2 Hz, phthalazine), δ 6.99–6.97 (2H, d, *J* = 7.8 Hz, Ar-H), δ 6.88–6.86 (1H, m, Ar-H), δ 6.85–6.83 (2H, d, *J* = 7.8 Hz, Ar-H), δ 3.34–3.32 (4H, m, piperazine ring), δ 3.22–3.20 (4H, m, piperazine ring), δ 2.48 (3H, *s*, CH_3_); FT-IR (ύ max, cm^−1^): 3070 (CH aromatic), 2954 (CH aliphatic); **MS** (Mwt.: 304.39): *m/z* 304.00(M+, 1.73%), 284.60 (2.19%), 192.35 (5.24%), 105.20 (34.33%), 55.65 (100.00%); Anal. Calcd for C_19_H_20_N_4_: C, 74.97; H, 6.62; N, 18.41; Found: C, 80.32; H, 6.70; N, 18.48.

##### 6.1.5.8. 1–(4-(2-Fluorophenyl)piperazin-1-yl)-4-methylphthalazine (8 h)

Yield (0.12 g; 71%), mp 149–151 °C; **^1^HNMR (300 MHz, CDCl_3_)**: δ 8.15–8.13 (1H, d, *J* = 9.6 Hz, phthalazine), δ 8.05–8.01 (1H, d, *J* = 9.6 Hz, phthalazine), δ 7.87–7.83 (2H, d, *J* = 9.6 Hz, phthalazine), δ 7.07–7.04 (2H, d, *J* = 8.7 Hz, Ar-H), δ 7.01–7.00 (2H, d, *J* = 8.7 Hz, Ar-H), δ 3.69–3.67 (4H, m, piperazine ring), δ 3.39–3.36 (4H, *m*, piperazine ring), δ 2.92 (3H, s, CH_3_); **^13^CNMR (100 MHz, DMSO)** *:* δ 173.5, 158. 9, 153.1, 137.4, 131.8, 131.5, 127.2, 125.2, 123.2, 123.1, 122.3, 120.4, 119.3, 116.1, 115.8, 50.9, 50.0, 47.0, 19.0, FT-IR (ύ max, cm^−1^): 3035 (CH aromatic), 2951 (CH aliphatic); **MS** (Mwt.: 322.38): *m/z* 323.50 [M + 1H]^+^ (9.74%), 172.15 (48.77%,), 122.05 (100.00%); Anal. Calcd for C_19_H_19_FN_4_: C, 70.79; H, 5.94; N, 17.38; Found: C, 71.03; H, 5.99; N, 18.48.

##### 6.1.5.9. 1-Methyl-4–(4-(pyridin-2-yl)piperazin-1-yl)phthalazine (8i)

Yield (0.10 g; 59%), mp 226–228 °C; **^1^HNMR (300 MHz, DMSO)**: δ 8.16–8.13(1H, d, *J* = 8.7 Hz, pyridyl ring), δ 7.62–7.60 (2H, d, *J* = 8.4 Hz, phthalazine), δ 7.58–7.56 (2H, d, *J* = 8.4 Hz, phthalazine), δ 6.91–6.89 (1H, d, *J* = 8.7 Hz, pyridyl ring), δ 6.74–6.72 (2H, d, *J* = 8.7 Hz, pyridyl ring), δ 3.75–3.73 (4H, m, piperazine ring), δ 3.12–3.10 (4H, m, piperazine ring), δ 2.08 (3H, *s*, CH_3_); FT-IR (ύ max, cm^−1^) 3132 (CH aromatic), 2954 (CH aliphatic); **MS** (Mwt.: 305.38): *m/z* 305.15 (M+, 1.16%), 185.65 (16.50%), 172.35 (41.23%,), 55.55 (100.00%); Anal. Calcd for C_18_H_19_N_5_: C, 70.80; H, 6.27; N, 22.93; Found: C, 71.04; H, 6.38; N, 23.18.

##### 6.1.5.10. Furan-2-yl-(4–(4-methylphthalazin-1-yl)piperazin-1-yl)methanone (8j)

Yield (0.11 g; 60%), mp 90–92 °C; **^1^HNMR (300 MHz, DMSO)**: δ 8.32–8.30 (2H, d, *J* = 7 Hz, phthalazine), δ 8.29–8.27 (2H, d, *J* = 7 Hz, phthalazine), δ 7.82–7.81 (1H, d, *J* = 7.2 Hz, furoyl ring), δ 6.97–6.96(1H, d, *J* = 7.2 Hz, furoyl ring), δ 6.62–6.60 (1H, d, *J* = 7.2 Hz, furoyl ring), δ 3.64–3.62 (4H, m, piperazine ring), δ 3.41–3.39 (4H, *m*, piperazine ring), δ 2.92 (3H, *s*, CH_3_); FT-IR (ύ max, cm-^1^): 1610 (C=O); **MS** (Mwt.: 322.36): m/z 322.00 (M+, 6.60%), 184.00 (31.70%), 172.00 (100.00%); Anal. Calcd for C_18_H_18_N_4_O_2_: C, 67.07; H, 5.63; N, 17.38; Found: C, 67.21; H, 5.69; N, 17.52.

#### 6.1.6. Synthesis of N^1^-(4-chlorophthalazin-1-yl)benzene-1,4-diamine (11)

General procedure:p-Phenylenediamine (0.33 g, 3.12 mmol, 3equiv) and the dichlorophthalazine (2.08 mmol, 2equiv) were treated with 2-BuOH (7.5 ml) in a tube and heated to 110 °C. The reaction quickly became a solid, yellow mass. After four hours, the reaction was cooled and diluted with water. The resultant slurry was then partitioned between equal volumes of DCM and 1 N NaOH (15 ml). The aqueous layer was extracted into DCM (2 × 15 ml). The combined organic layers were dried over anhydrous sodium sulfate, filtered, and concentrated *in vacuo*. The resulting orange solid was crystallised from (EtOAc/pet ether 1:1) to afford the title compound **(11)** as an orange crystals.

Yield 0.22 g, (88%); mp 170–172 °C; **^1^HNMR (300 MHz, DMSO-d_6_)**: δ 13.21 (*s*, 1H, –NH D_2_O exchangeable), 9.27 (*s*, 2H, 2H, –NH_2_ D_2_O exchangeable), δ 8.30–8.28 (d, *J* = 7 Hz, 2H, phthalazine), δ 8.07–8.04 (d, *J* = 7 Hz, 2H, phthalazine), δ 7.46–7.43 (d, *J* = 9 Hz, 2H, ArH), δ 6.83–6.76 (d, *J* = 9 Hz, 2H, ArH), δ 2.48 (*s*, 3H, CH_3_); FT-IR (υ max, cm^−1^): 3385 (NH_2_), 3321(NH); **MS** (Mwt.: 270.72): *m/z* 250.00 (M+, 50.00%), 249.00 (100.00%), 108.00 (60.92%), 65.00 (77.59%); Anal. Calcd for C_14_H_11_ClN_4_: C, 62.11; H, 4.10; N, 20.70; Found: C, 62.37; H, 4.18; N, 20.94.

#### 6.1.7. Synthesis of 1-aryl-3–(4-(4-substituted phthalazin-1-ylamino)phenyl)urea (12a-c)

General procedure:

To a stirred solution of the N1-(4-chlorophthalazin-1-yl)benzene-1,4-diamine **(11)** (10.0 mmol, 1equiv) in DMF (20 ml), the respective phenyl isocyanate (viz.; phenyl isocyanate, 4-chloro phenyl isocyanate, 3-trifluromethyl-4-chlorophenyl isocyanate) (10.0 mmol, 1equiv) was added and the mixture was heated under reflux for 8–10 h, after which TLC (CH_2_Cl_2_/CH_3_OH 9:1) showed no starting material. The reaction mixture was poured over ice-water; the formed precipitate was allowed to settle, then filtered off and dried to afford the crude product **(12a-c)** which was further crystallised from EtOAc.

##### 6.1.7.1. 1–(4-(4Chlorophthalazin-1-ylamino)phenyl)-3-phenylurea (12a)

Yield 0.12 g; (68%), mp 225–227 °C; **^1^HNMR (400 MHz, DMSO-d_6_)**: δ 12.85 (*s*, 3H, –NH D_2_O exchangeable), δ 8.63 (d, *J* = 10 Hz 1H, phthalazine), δ 8.30–8.27 (d, *J* = 10 Hz, 2H, phthalazine), δ 8.08–8.05 (d, *J* = 10 Hz, 1H, phthalazine), δ 8.03–7.02 (d, *J* = 8 Hz, 2H, Ar-H), δ 7.99–7.97 (d, *J* = 8 Hz, 2H, Ar-H), δ 7.95–7.94 (d, *J* = 8 Hz, 1H, Ar-H), δ 7.46–7.43 (d, *J* = 9.2 Hz, 2H, Ar-H), δ 7.29–7.24 (d, *J* = 9.2 Hz, 1H, Ar-H), δ 6.98–6.94 (d, *J* = 9.2 Hz, 1H, Ar-H); **^13^CNMR (100 MHz, DMSO)** *:* δ 159.0, 152.5, 146.1, 139.6, 137.0, 134.4, 132.9, 128.7, 128.3, 126.4, 125,4, 121.7, 120.5, 119.5, 118.1; FT-IR (υ max, cm^−1^): 3296, 3286, 3155 (3NH), 1668 (C=O); **MS** (Mwt.: 389.84): *m/z* 389.28 (M^+^, 2.84%), 367.27 (6.23%), 322.10 (11.84%), 128.10(100.00%); Anal. Calcd for C_21_H_16_ClN_5_O: C, 64.70; H, 4.14; N, 14.96; Found: C, 64.71; H, 3.89; N, 14.52.

##### 6.1.7.2. 1–(4-Chlorophenyl)-3–(4-(4-chlorophthalazin-1-ylamino)phenyl)urea (12b)

Yield 0.20 g; (53%), mp 236–238 °C; **^1^HNMR (400 MHz, DMSO-d_6_)**: δ 12.84 (*s*, 2H, –NH D_2_O exchangeable), δ 8.81 (*s*, 1H, –NH D_2_O exchangeable), δ 8.29–8.27 (d, *J* = 8.8 Hz, 1H, phthalazine), δ 8.07–8.05 (d, *J* = 8.8 Hz, 1H, phthalazine), δ 8.02–8.00 (d, *J* = 8.8 Hz, 1H, phthalazine), δ 7.98–7.96 (d, *J* = 8.8 Hz, 1H, phthalazine), δ 7.95–7.94 (d, *J* = 6.4 Hz, 2H, Ar-H), δ 7.57–7.56 (d, *J* = 6.4 Hz, 2H, Ar-H), δ 7.48–7.47 (d, *J* = 6.4 Hz, 2H, Ar-H), δ 7.31–7.30 (*m*, 2H, Ar-H); FT-IR (υ max, cm^−1^): 3446, 3300, 3157 (3NH), 1670 (C=O); **MS** (Mwt.: 423.07): *m/z* 424.88 [M + 1H]^+^ (3.74%), 423.50 (M^+^, 9.84%), 328.27 (52.58%), 252.97 (20.15%), 119.04(100.00%); Anal. Calcd for C_21_H_15_Cl_2_N_5_O: C, 59.45; H, 3.56; N, 16.51; Found: C, 59.49; H, 3.37; N, 16.29.

##### 6.1.7.3. 1–(4-Chloro-3-(trifluoromethyl)phenyl)-3–(4-(4-chlorophthalazin-1-ylamino)phenyl)-urea (12c)

Yield 0.22 g; (49%), mp 187–189 °C; **^1^HNMR (400 MHz, DMSO-d_6_)**: δ 12.84 (*s*, 2H, –NH D_2_O exchangeable), δ 9.36 (*s*, 1H, –NH D_2_O exchangeable), δ 8.29–8.27 (d, *J* = 8.8 Hz, 2H, phthalazine), δ 8.27–8.25 (d, *J* = 8.8 Hz, 2H, phthalazine), δ 8.09 (*s*, 1H, Ar-H), 8.05–8.03 (d, *J* = 9.6 Hz, 1H, Ar-H), δ 8.01–7.97 (d, *J* = 9.6 Hz, 1H, Ar-H), δ 7.59–7.50 (d, *J* = 8.4 Hz, 2H, Ar-H), δ 7.39–7.37 (d, *J* = 8.4 Hz, 2H, Ar-H); FT-IR (υ max, cm^−1^): 3387, 3344, 3290 (3NH), 1664(C=O); **MS** (Mwt.: 492.28): *m/z* 493.06 [M + 1H]^+^ (2.63%), 395.08 (32.95%), 372.90 (35.24%), 137.07 (100.00%); Anal. Calcd for C_22_H_14_Cl_2_F_3_N_5_O: C, 53.68; H, 2.87; N, 14.23; Found: C, 53.72; H, 2.68; N, 14.44.

#### 6.1.8. Synthesis of 1-aryl-3–(4-(4-chlorophthalazin-1-yloxy)phenyl)urea (13a-c)

General procedure:

To a stirred solution of 1,4 dichlorophthalazine derivative (**10**) (10.0 mmol, 1equiv), and cesium carbonate (6.5 g, 20.0 mmol, 2equiv) in acetonitrile (20 ml), the appropriate 1-aryl-3–(4-hydroxyphenyl)urea (**1a-c**) (10.0 mmol, 1equiv) was added and the mixture was heated under reflux for 6 h, after which TLC (CHCl_3_/CH_3_OH 9:1) showed no starting material. The solvent was evaporated under vacuum and the resultant solid was stirred with cold 1 M NaOH solution (20 ml), filtered off, dried and recrytallised from acetonitrile to afford compounds (**13a-c**).

##### 6.1.8.1. 1–(4-(4-Chlorophthalazin-1-yloxy)phenyl)-3-phenylurea (13a)

Yield 0.12 g; (46%), mp 191–193 °C; **^1^HNMR (300 MHz, DMSO-d_6_)**: δ 10.48 (*s*, 1H, –NH D_2_O exchangeable), δ 10.31 (*s,* 1H, –NH D_2_O exchangeable), δ 8.46–8.44 (d, *J* = 8 Hz, 2H, phthalazine), δ 8.29–8.27 (d, *J* = 8 Hz, 2H, phthalazine), δ 7.90–7.88 (d, *J* = 8.6 Hz, 2H, Ar-H), δ 7.69–7.66 (d, *J* = 8.6 Hz, 2H, Ar-H), δ 7.40–7.37 (d, *J* = 8 Hz, 1H, Ar-H), δ 7.27–7.25 (d, *J* = 8 Hz, 2H, Ar-H), δ 6.94–6.92 (d, *J* = 8 Hz, 2H, Ar-H); **^13^CNMR (100 MHz, DMSO)** *:* δ 164.7, 158.8, 153. 8, 152.4, 141.3, 136.6, 135.3, 132.3, 128.4, 128.0, 127.3, 125. 7, 123.8, 121.6, 120.6, 120.2, 118.1, 115.1; FT-IR (υ max, cm^−1^): 3325, 3295 (2NH), 1662 (C=O); **MS** (Mwt.: 390.82): *m/z* 390.97.00 (M^+^, 2.24%), 346.01 (5.06%), 268.11 (18.11%), 179.11 (100.00%); Anal. Calcd for C_21_H_15_ClN_4_O_2_: C, 64.54; H, 3.87; N, 14.43; Found: C, 64.97; H, 4.21; N, 14.12.

##### 6.1.8.2. 1–(4-Chlorophenyl)-3–(4-(4-chlorophthalazin-1-yloxy)phenyl)urea (13b)

Yield 0.19 g; (50%), mp 172–174 °C; **^1^HNMR (400 MHz, DMSO-d_6_)**: δ 9.06 (*s*, 1H, –NH D_2_O exchangeable), δ 8.54 (*s*, 1H,–NH D_2_O exchangeable), δ 8.34–8.32 (d, *J* = 8.4 Hz, 2H, phthalazine), δ 8.18–8.16 (d, *J* = 8.4 Hz, 2H, phthalazine), δ 7.44–7.42 (d, *J* = 8.8 Hz, 2H, Ar-H), δ 7.35–7.33 (d, *J* = 8.8 Hz, 2H, Ar-H), δ 7.27–7.25 (d, *J* = 7.2 Hz, 2H, Ar-H), δ 6.78–6.76 (d, *J* = 7.2 Hz, 2H, Ar-H); FT-IR (υ max, cm^−1^): 3278, 3155 (2NH), 1666 (C=O); **MS** (Mwt.: 425.27): *m/z* 425.28 (M^+^, 5.29%), 305.13 (7.74%), 201.08 (11.73%), 63.03 (100.00%); Anal. Calcd for C_21_H_14_Cl_2_N_4_O_2_: C, 59.31; H, 3.32; N, 13.17; Found: C, 59.62; H, 3.60; N, 13.65.

##### 6.1.8.3. 1–(4-Chloro-3-(trifluoromethyl)phenyl)-3–(4-(phthalazin-1-yloxy)phenyl)urea (13c)

Yield 0.22 g; (49%), mp 210–212 °C; **^1^HNMR (400 MHz, DMSO-d_6_**): δ 10.20 (*s*, 1H, –NH D_2_O exchangeable), δ 10.13 (*s*, 1H, –NH D_2_O exchangeable), δ 8.28–8.26 (d, *J* = 9 Hz, 2H, phthalazine), δ 8.25–8.23 (d, *J* = 9 Hz, 2H, phthalazine), δ 8.09 (*s*, 1H, Ar-H), δ 7.93–7.92 (d, *J* = 7.6 Hz, 1H, Ar-H), δ 7.76–7.74 (d, *J* = 7.6 Hz, 1H, Ar-H), δ 7.54–7.51 (d, *J* = 8.4 Hz, 2H, Ar-H), δ 6.69–6.66 (d, *J* = 8.4 Hz, 2H, Ar-H); FT-IR (υ max, cm^−1^): 3294, 3155(2NH), 1662 (C=O); **MS** (Mwt.: 493.27): 493.46 (M^+^, 1.24%), 281.00 (2.26%), 194.97(100.00%); Anal. Calcd for C_22_H_13_Cl_2_F_3_N_4_O_2_: C, 53.57; H, 2.66; N, 11.36; Found: C, 53.81; H, 2.90; N, 11.3.

#### 6.1.9. Synthesis of N-(4-chlorophenyl)-4-methylphthalazin-1-amine (14)

General procedure:

To a stirred mixture of the 1-chloro-4-methyl-phthalazine **(3b)** (1.78, 10.0 mmol, 1 equiv) in butanol (50 ml), triethylamine (1 ml, 10.0 mmol, 1 equiv) the respective amine (viz.; aniline, p-Cl aniline) (10.0 mmol, 1equiv) was added and the mixture was heated under reflux for 2 h, after which TLC (CHCl_3_/CH_3_OH 9:1) showed no starting material. The mixture was then concentrated *in vacuo*, the crude product was recrystallised from methanol.

Yield 1.56 g; (88%), mp 213–215 °C; ^1^HNMR (400 MHz, DMSO-d_6_): δ 10.47 (*s*, 1H, –NH D_2_O exchangeable), δ 9.10–9.07 (d, *J* = 9 Hz, 1H, phthalazine), δ 8.45 –8.44 (d, *J* = 9 Hz, 1H, phthalazine), δ 8.31–8.29 (d, *J* = 9 Hz, 1H, phthalazine), δ 8.23–8.20 (d, *J* = 9 Hz, 1H, phthalazine), δ 7.85–7.82 (d, *J* = 8 Hz, 1H, Ar-H) , δ 7.51–7.49 (d, *J* = 8 Hz, 1H, Ar-H), δ 7.10–7.07 (d, *J* = 8 Hz, 1H, Ar-H), δ 6.70–6.68 (d, *J* = 8 Hz, 1H, Ar-H) , δ 2.97 (*s*, 3H, CH_3_); FT-IR (υ max, cm^−1^) 3360 (NH), 3050 (CH aromatic); **MS** (Mwt.: 269.73): *m/z* 269.28 (M^+^, 8.26%), 239.18 (15.43%), 125.22 (24.70%), 90.97(100.00%); Anal. Calcd for C_15_H_12_ClN_3_: C, 66.79; H, 4.48; N, 15.58; Found: C, 67.03; H, 4.57; N, 15.75.

#### 6.1.10. Synthesis of 4-(bromomethyl)-N-(4-chlorophenyl)phthalazin-1-amine (15)

General procedure:

A mixture of the N-aryl-4-methylphthalazin-1-amine (**14b**) (0.26 mmol, 1 equiv), N-bromo-succinimide (4.6 g, 0.26 mmol, 1 equiv) and dibenzoyl peroxide (0.44 g, 0.018 mmol, 0.07 equiv) in carbon tetrachloride (150 ml) was refluxed for 24 h. The reaction mixture was cooled to 40 °C, and then filtered. The filtrate was concentrated under vacuum to afford the crude product, which was triturated with ether to give a yellowish solid product.

Yield 0.10 g; (42%), mp 172.-174 °C; **^1^HNMR (400 MHz, DMSO-d_6_)**: δ 11.06 (*s*, 1H, –NH D_2_O exchangeable), δ 8.07–8.05 (d, *J* = 7.2 Hz, 2H, phthalazine), δ 7.99 –7.97 (d, *J* = 7.2 Hz, 2H, phthalazine), δ 7.62–7.60 (d, *J* = 7.6 Hz, 2H, Ar-H), δ 7.45–7.44 (d, *J* = 7.6 Hz, 2H, Ar-H), δ 4.02 (s, 2H, CH_2_); FT-IR (υ max, cm^−1^) 3450 (NH), 3053 (CH aromatic); **MS** (Mwt.: 348.62): *m/z* 350.00 (M^+2^, 2.86%), 348.00 (M^+^, 9.89%), 268.04 (65.94), 102.03 (100.00%); Anal. Calcd for C_15_H_11_BrClN_3_: C, 51.68; H, 3.18; N, 12.05; Found: C, 51.90; H, 3.16; N, 12.18.

#### 6.1.11. Synthesis of N-(4-chlorophenyl)-4–(3-substitutedphenylamino)methyl)phthalazin-1-amines (16a-d)

General procedure:

To a stirred solution of 4-(bromomethyl)-N-(4-chlorophenyl)phthalazin-1-amine (**15**) (10.0 mmol, 1 equiv), potassium carbonate (0.27 g, 20.0 mmol, 2equiv), potassium iodide (0.01 g, 0.01 mmol, 0.1equiv) in acetone (20 ml), the respective amine (viz.; aniline, m-Chloroaniline, m-touilidine, m-anisidine) (10.0 mmol, 1equiv) was added and the mixture was heated under reflux for 4–6 h, after which TLC (CH_2_Cl2/CH_3_OH 99:1) showed no starting material. The mixture was then filtered and the filtrate concentrated *in vacuo*, the residue was crystallised from acetone.

##### 6.1.11.1. N-(4-Chlorophenyl)-4-((phenylamino)methyl)phthalazin-1-amine (16a)

Yield 0.10 g; (42%), mp 120–122 °C; **^1^HNMR (400 MHz, DMSO-d_6_)**: δ 11.03 (*s*, 1H, –NH D_2_O exchangeable), δ 9.65 (*s*, 1H, –NH D_2_O exchangeable), δ 8.75–8.72 (d, *J* = 9.2 Hz, 2H, phthalazine), δ 8.28 –8.25 (d, *J* = 9.2 Hz, 2H, phthalazine), δ 7.90–7.88 (d, *J* = 8.6 Hz, 2H, Ar-H), δ 7.85–7.87 (d, *J* = 8.6 Hz, 2H, Ar-H), δ 7.46–7.43 (d, *J* = 7.6 Hz, 1H, Ar-H), δ 7.05–7.03 (d, *J* = 7.6 Hz, 2H, Ar-H), δ 6.83–6.81 (d, *J* = 7.6 Hz, 2H, Ar-H), δ 3.89 (*s*, 2H, CH_2_); FT-IR (υ max, cm^−1^) 3446, 3383 (2NH), 3068 (CH aromatic), 2976 (CH aliphatic); **MS** (Mwt.: 360.84): *m/z* 360.02 (M^+,^ 2.87%), 345.88 (13.64%), 268.02 (49.93%), 75.01 (100%); Anal. Calcd for C_21_H_17_ ClN_4_: C, 69.90; H, 4.75; N, 15.53; Found: C, 70.12; H, 4.82; N, 15.69.

##### 6.1.11.2. N-(4-Chlorophenyl)-4-((3-chlorophenylamino)methyl)phthalazin-1-amine (16b)

Yield 0.10 g; (42%), mp 164–166 °C; **^1^HNMR (400 MHz, DMSO-d_6_)**: δ 9.90 (s, 2H, –NH D_2_O exchangeable), δ 8.86–8.84 (d, *J* = 9.2 Hz, 2H, phthalazine), δ 8.34 –8.32 (d, *J* = 9.2 Hz, 2H, phthalazine), δ 8.20–8.18 (d, *J* = 8.6 Hz, 2H, Ar-H), δ 8.12–8.09 (d, *J* = 8.6 Hz, 2H, Ar-H), δ 7.88–7.86 (d, *J* = 7.6 Hz, 1H, Ar-H), δ 7.53–7.51 (d, *J* = 7.6 Hz, 1H, Ar-H), δ 7.44–7.42 (d, *J* = 7.6 Hz, 2H, Ar-H), δ 3.79 (s, 2H, CH_2_); FT-IR (υ max, cm^−1^) 3379 (2NH), 3080 (CH aromatic), 2954 (CH aliphatic); **MS** (Mwt.: 395.28) *m/z* 395.99 (M^+,^ 2.17%), 271.01 (16.80%), 268.01 (100%); Anal. Calcd for C_21_H_16_Cl_2_N_4_: C, 63.81; H, 4.08; N, 14.17; Found: C, 63.94; H, 4.12; N, 14.28.

##### 6.1.11.3. N-(4-Chlorophenyl)-4-((m-tolylamino)methyl)phthalazin-1-amine (16c)

Yield 0.10 g; (42%), mp 130–132 °C; ^1^HNMR (400 MHz, DMSO-d_6_): δ 9.70 (*s*, 2H, –NH D_2_O exchangeable), δ 8.86–8.84 (d, *J* = 9.2 Hz, 2H, phthalazine), δ 8.34 –8.32 (d, *J* = 9.2 Hz, 2H, phthalazine), δ 8.20–8.18 (d, *J* = 8.6 Hz, 2H, Ar-H), δ 8.12–8.09 (d, *J* = 8.6 Hz, 2H, Ar-H), δ 7.88–7.86 (d, *J* = 7.6 Hz, 1H, Ar-H), δ 7.53–7.51 (d, *J* = 7.6 Hz, 1H, Ar-H), δ 7.44–7.42 (d, *J* = 7.6 Hz, 2H, Ar-H), δ 3.79 (*s*, 2H, CH_2_), δ 2.69 (s, 3H, CH_3_);): **^13^CNMR (100 MHz, DMSO)**: δ 162.2, 152.9, 151.1, 138.9, 135.0, 133.8, 131.7, 127.3, 127.1, 126.8, 126.4, 126.3, 124.7, 124.3, 124.1, 120.5, 120.2, 117.4, 113.2, 110.5, 45.5, 16.7, FT-IR (υ max, cm^−1^) 3446, 32992 (2NH), 3050 (CH aromatic), 2922 (CH aliphatic); **MS** (Mwt.: 374.13): *m/z* 374.75 (M^+,^ 2.65%), 268.06 (21.45%), 189.95 (18.36%), 77.06 (100%); Anal. Calcd for C_22_H_19_ClN_4_: C,70.49; H, 5.11; N, 14.95; Found: C, 70.73; H, 5.19; N, 15.08.

##### 6.1.11.4. N-(4-Chlorophenyl)-4-((3-methoxyphenylamino)methyl)phthalazin-1-amine (16d)

Yield 0.10 g; (42%), mp 175–177 °C; **^1^HNMR (400 MHz, DMSO-d_6_)**: δ 11.34 (*s*, 1H, –NH D_2_O exchangeable), δ 9.78 (*s*, 1H, –NH D_2_O exchangeable), δ 8.73–8.70 (d, *J* = 9.6 Hz, 2H, phthalazine), δ 8.48 –8.46 (d, *J* = 9.6 Hz, 2H, phthalazine), δ 8.06–8.04 (d, *J* = 8.6 Hz, 2H, Ar-H), δ 7.87–84 (d, *J* = 8.6 Hz, 2H, Ar-H), δ 7.68–7.66 (d, *J* = 7.5 Hz, 1H, Ar-H), δ 7.46–7.44 (d, *J* = 7.5 Hz, 2H, Ar-H), δ 7.14–7.12 (d, *J* = 7.5 Hz, 1H, Ar-H), δ 4.29 (*s*, 2H, CH_2_), δ 3.63 (*s*, 3H, OCH_3_); **^13^CNMR (100 MHz, DMSO)** *:* δ 164.2, 163.9, 148.7, 148.8, 139.2, 132.3, 131.3, 129.6, 128.5, 127.9, 123.19, 122.1, 120.4, 120.4, 115.1, 110.3, 109.2, 106.0, 105.7, 105.0, 51.2, 45.7;FT-IR (υ max, cm^−1^) 3412, 3385 (2NH), 3041(CH aromatic), 2976 (CH aliphatic); **MS** (Mwt.: 390.87): *m/z* 391.95 (M+, 7.73%), 350.00 (29.42%), 348.01 (100.00%), 313.04 (10.74%); Anal. Calcd for C_22_H_19_ ClN_4_O: C, 67.60; H, 4.90; N, 14.33; Found: C, 67.91; H, 4.95; N, 14.50.

#### 6.1.12. Synthesis of N-(4-chlorophenyl)-4-(aryloxymethyl)phthalazin-1-amine (17a,b)

General procedure:

To a stirred solution of 4-(bromomethyl)-N-(4-chlorophenyl)phthalazin-1-amine (**15)** (10.0 mmol, 1 equiv), sodium hydride (0.27 g, 20.0 mmol, 2equiv) in acetone (20 ml), the respective phenol (viz.; phenol, m-cresol) (10.0 mmol, 1equiv) was added and the mixture was heated under reflux for 6–8 h, after which TLC (CH_2_Cl_2_/CH_3_OH 99:1) showed no starting material. The mixture was then filtered and the filtrate concentrated *in vacuo*, the residue was crystallised from acetone.

##### 6.1.12.1. N-(4-Chlorophenyl)-4-(phenoxymethyl)phthalazin-1-amine (17a)

Yield 0.10 g; (42%), mp 242–244 °C; **^1^HNMR (400 MHz, DMSO-d_6_)**: δ 9.33 (*s*, 1H,–NH D_2_O exchangeable), δ 8.74–8.75 (d, *J* = 9.2 Hz, 2H, phthalazine), δ 8.30 –8.28 (d, *J* = 9.2 Hz, 2H, phthalazine), δ 8.11–8.09 (d, *J* = 8.6 Hz, 2H, Ar-H), δ 7.89–7.87 (d, *J* = 8.6 Hz, 2H, Ar-H), δ 7.49–7.47 (d, *J* = 7.6 Hz, 2H, Ar-H), δ 7.17–7.15 (d, *J* = 7.6 Hz, 1H, Ar-H), δ 6.76–6.74 (d, *J* = 7.6 Hz, 2H, Ar-H), δ 3.89 (s, 2H, CH_2_); FT-IR (υ max, cm^−1^) 3379 (NH), 3066 (CH aromatic), 2978 (CH aliphatic); **MS** (Mwt.: 361.82): *m/z* 361.00 (M^+^, 5.29%), 349.01 (17.15%), 310.24 (12.19%), 268.11 (64.63%), 133.44 (100.00%); Anal. Calcd for C_21_H_16_ClN_3_O: C, 69.71; H, 4.46; N, 11.61; Found: C, 69.88; H, 4.39; N, 11.69.

##### 6.1.12.2. N-(4-Chlorophenyl)-4-(m-tolyloxymethyl)phthalazin-1-amine (17b)

Yield 0.10 g; (42%), mp 193–195 °C; **^1^HNMR (400 MHz, DMSO-d_6_):** δ 9.33 (s, 1H,–NH D_2_O exchangeable), δ 8.74–8.72 (d, *J* = 8.8 Hz, 2H, phthalazine), δ 8.31–8.28 (d, *J* = 8.8 Hz, 2H, phthalazine), δ 8.14–8.11 (d, *J* = 8.6 Hz, 2H, Ar-H), δ 8.09–8.06 (d, *J* = 8.6 Hz, 2H, Ar-H), δ 7.49–7.47 (d, *J* = 7.6 Hz, 1H, Ar-H), δ 7.47–7.44 (d, *J* = 8.6 Hz, 2H, Ar-H), δ 6.55–6.53 (d, *J* = 8.6 Hz, 1H, Ar-H), δ 3.89 (*s*, 2H, CH_2_), δ 2.19 (*s*, 3H, CH_3_); **^13^CNMR (100 MHz, DMSO)** *:* δ 173.4, 162.4, 151.0, 139.4, 138.4, 133.1, 132.1, 128.9, 128.6, 128.4, 126.9, 126.9, 125.9, 123.3, 123.15, 121.9, 119.4, 115.7, 112.2, 75.4, 20.9, FT-IR (υ max, cm^−1^) 3414 (NH), 3034 (CH aromatic), 2976 (CH aliphatic); **MS** (Mwt.: 375.85): *m/z* 375.94 (M^+^, 1.09%), 349.94 (4.65%), 267.15 (100.00%); Anal. Calcd for C_22_H_18_ClN_3_O: C, 70.30; H, 4.83; N, 11.18; Found: C, 70.43; H, 4.88; N, 11.32.

## Supplementary Material

Supplemental Material
