# Supplementary material for: Design and synthesis of phthalazine-based compounds as potent anticancer agents with potential antiangiogenic activity via VEGFR-2 inhibition
Source: J Enzyme Inhib Med Chem. 2019 Jul 19;34(1):1347–67. doi: 10.1080/14756366.2019.1642883 (PMC6691788; doi:10.1080/14756366.2019.1642883)
Supplement: Supplemental Material [file IENZ_A_1642883_SM7309.docx]

**Table S1: *In vitro* NCI 60 cell line anticancer screening results of compounds (6b, 6e, 7b) at single dose of 10 μM presented as percent cell growth promotion.**

| Panel/Cell Line | 6b | 6e | 7b |  | Panel/Cell Line | 6b | 6e | 7b |
| --- | --- | --- | --- | --- | --- | --- | --- | --- |
| **Leukemia**  CCRF-CEM  HL-60(TB)  K-562  MOLT-4  RPMI-8226  SR | 13.13  18.13  **9.49**  **-1.61**  **8.62**  17.49 | 11.02  **-34.81**  **-16.67**  **9.76**  **-11.88**  **-3.51** | **8.70**  **3.65**  **5.58**  **3.99**  **-0.08**  **2.64** |  | **Melanoma**  LOX IMVI  MALME-3M  M14  MDA-MB-435  SK-MEL-28  SK-MEL-5  UACC-257  UACC-62 | 33.64  38.46  19.31  13.74  44.13  **7.12**  15.33  13.41 | **-27.27**  **-9.90**  37.26  **-39.98**  **-11.92**  52.34  73.78  **-34.42** | **-77.27**  **0.78**  **7.30**  **-33.34**  **-31.58**  **-43.28**  34.44  **-14.30** |
| **Non-Small Cell Lung Cancer**  A549/ATCC  HOP-62  HOP-92  NCI-H226  NCI-H23  NCI-H322M  NCI-H460  NCI-H522 | 20.05  20.57  38.66  33.32  12.46  64.33  9.89  11.92 | 26.37  44.24  4.62  71.92  48.79  47.83  26.11  30.26 | 40.00  26.74  12.92  -54.51  0.50  60.93  21.50  -46.35 |  | **Ovarian Cancer**  IGROV1  OVCAR-3  OVCAR-4  OVCAR-5  OVCAR-8  NCI/ADR-RES  SK-OV-3 | 39.69  17.58  11.14  85.59  18.24  36.17  38.21 | 61.98  44.40  42.61  72.85  31.43  45.08  66.42 | **8.16**  **-62.82**  **-20.81**  **-16.00**  19.42  37.64  56.69 |
| **Colon Cancer**  COLO 205  HCC-2998  HCT-116  HCT-15  HT29  KM12  SW-620 | 18.58  30.69  15.20  18.27  32.43  18.93  26.99 | 28.17  40.46  **7.67**  **6.39**  **2.24**  14.10  20.31 | 11.92  **-42.18**  **-56.86**  **2.76**  **0.52**  **-32.31**  **-30.09** |  | **Renal Cancer**  786-0  A498  ACHN  CAKI-1  RXF 393  SN12C  TK-10  UO-31 | 50.39  24.54  22.43  20.58  65.65  26.41  31.96  18.97 | **-27.85**  35.41  58.77  33.85  **-39.72**  60.11  51.66  51.21 | N/A  24.52  **-15.09**  18.67  **-91.21**  **-3.83**  27.53  **-51.60** |
| **CNS Cancer**  SF-268  SF-295  SNB-19  SNB-75  U251 | 42.42  17.82  32.96  46.25  18.31 | 48.73  **4.80**  16.20  68.13  **5.40** | 12.64  40.69  **-76.47**  **-3.13**  **-31.11** |  | **Breast Cancer**  MCF7  MDA-MB 231/ATCC  HS 578T  BT-549  T-47D  MDA-MBA-468 | **5.46**  36.57  58.32  15.70  **-6.32**  **0.17** | 24.87  23.06  **3.43**  11.41  8.43  **6.44** | 14.27  **-25.22**  37.47  **-10.33**  **10.20**  **-76.16** |
| **Prostate Cancer**  PC-3  DU-145 | **1.46**  33.71 | 22.81  57.80 | **-29.02**  11.15 |  | **Mean** | 25.03 | 23.23 | **-6.99** |

* Results are presented as percent cell growth promotion.

* Values less than 10% growth promotion are presented in bold.

**Table S2: *In vitro* NCI 60 cell line anticancer screening results of compounds (13a, 13c, 16a, 16d & 17a) at single dose of10 μM presented as percent cell growth promotion.**

| **Panel/Cell Line** | **13a** | **13c** | **16a** | **16d** | **17a** | **Panel/Cell Line** | **13a** | **13c** | **16a** | **16d** | | **17a** |
| --- | --- | --- | --- | --- | --- | --- | --- | --- | --- | --- | --- | --- |
| **Leukemia**  **CCRF-CEM**  **HL-60(TB)**  **K-562**  **MOLT-4**  **RPMI-8226**  **SR** | 47.58  12.07  51.64  N/A  35.10  17.99 | **-5.44**  **-16.72**  **-8.66**  N/A  **-15.93**  **-23.81** | 12.39  **-13.35**  **5.39**  **-9.21**  **3.35**  **-9.19** | **-4.42**  **-27.06**  **2.20**  **-27.22**  **-28.06**  **-24.51** | **1.06**  **-15.73**  **2.77**  **-32.59**  **-25.26**  **-20.62** | **Melanoma**  LOX IMVI  MALME-3M  M14  MDA-MB-435  SK-MEL-2  SK-MEL-28  SK-MEL-5  UACC-257  UACC-62 | 32.10  **-29.55**  46.25  48.62  **9.53**  44.20  32.94  22.02  41.32 | **-93.23**  **-49.92**  **-62.78**  **-78.37**  **-72.06**  **-62.49**  **-98.76**  **-68.44**  **-82.50** | 12.14  **-13.85**  **5.80**  **4.17**  **8.63**  14.86  **-69.12**  **-6.49**  **8.47** | **-58.72**  **-21.98**  **-15.71**  **-10.28**  **-34.44**  **-16.09**  **-98.52**  **-39.68**  **-29.24** | **-61.98**  **-13.52**  **-9.99**  **-3.33**  **-26.47**  **4.73**  **-96.11**  **-24.86**  **-27.73** | |
| **Non-Small Cell Lung Cancer**  **A549/ATCC**  **EKVX**  **HOP-62**  **HOP-92**  **NCI-H226**  **NCI-H23**  **NCI-H322M**  **NCI-H460**  **NCI-H522** | 11.21  29.96  -**43.37**  **-7.51**  **8.66**  32.74  47.30  25.25  **2.00** | -**21.87**  **-6.74**  **-81.58**  **-38.36**  **-28.21**  **-52.39**  **-5.16**  **-77.36**  **-76.49** | 20.31  16.86  27.38  **-19.35**  25.02  **9.95**  30.42  17.65  **-5.92** | **2.56**  **-0.28**  **-17.14**  **-32.75**  **-22.86**  **2.05**  **3.32**  **0.10**  **-43.81** | **4.64**  **6.53**  **13.06**  **-27.96**  **1.78**  **4.18**  21.15  **3.10**  **-17.40** | **Ovarian Cancer**  IGROV1  OVCAR-3  OVCAR-4  OVCAR-5  OVCAR-8  NCI/ADR-RES  SK-OV-3 | 37.24  25.37  **0.22**  86.32  15.51  16.87  **-0.50** | **-16.79**  **-41.93**  **2.00**  **-7.38**  **-16.81**  **-28.17**  **-44.14** | 19.10  **-1.64**  19.30  41.45  18.25  18.81  22.04 | **1.51**  **-53.64**  17.22  **8.44**  **3.02**  **-5.42**  **-11.93** | | 16.13  **-35.08**  18.08  23.15  **5.02**  **-1.13**  **9.17** |
| **Colon Cancer**  **COLO 205**  **HCC-2998**  **HCT-116**  **HCT-15**  **HT29**  **KM12**  **SW-620** | 35.15  52.85  10.61  36.39  23.84  43.58  73.42 | **-69.30**  **-86.46**  **-71.56**  **-63.99**  **-75.94**  **-60.12**  **-62.85** | 11.88  18.29  **9.94**  13.36  **0.55**  **8.93**  30.81 | **-22.24**  **2.79**  **-44.16**  **-18.13**  **-21.02**  **-24.91**  11.91 | **-5.75**  **8.55**  **1.21**  **4.45**  **1.32**  **0.18**  15.36 | **Renal Cancer**  786-0  A498  ACHN  CAKI-1  RXF 393  SN12C  TK-10  UO-31 | 38.18  49.16  33.92  84.42  **-3.63**  53.70  25.28  34.87 | **-42.83**  **-82.71**  **-8.47**  11.70  **-62.34**  **-71.88**  **-51.47**  **-28.39** | 21.42  24.69  **7.07**  28.24  25.68  28.89  **-0.69**  **1.91** | **-5.76**  **-26.84**  **-5.35**  **9.01**  **-11.56**  14.48  **-40.85**  **-42.55** | | **5.25**  **-17.26**  **3.37**  15.44  **-5.93**  16.98  **-17.51**  **-12.31** |
| **CNS Cancer**  **SF-268**  **SF-295**  **SF-539**  **SNB-19**  **SNB-75**  **U251** | 25.94  31.18  -22.02  **9.64**  -41.95  **4.84** | **-32.25**  **-56.34**  **-73.39**  **-5.90**  **-59.30**  **-75.38** | 20.88  14.34  33.99  25.90  19.58  **9.66** | **2.52**  **-22.59**  **-10.19**  17.75  **-6.06**  **-56.23** | 13.18  **-1.11**  **8.42**  21.95  13.10  **-12.69** | **Breast Cancer**  MCF7  MDA-MB 231/ATCC  HS 578T  BT-549  T-47D  MDA-MBA-468 | **-4.15**  32.10  **1.19**  47.66  **-18.04**  **0.47** | **-35.57**  **-57.28**  **-17.22**  **-37.15**  **-24.62**  **-44.84** | **9.20**  19.91  26.58  **4.94**  **2.82**  **-16.37** | **-1.96**  **-27.10**  **0.54**  **-12.18**  **-8.49**  **-21.88** | | **1.31**  11.38  14.37  **-9.97**  **-5.86**  **-16.85** |
| **Prostate Cancer**  **PC-3**  **DU-145** | 25.95  39.01 | **-38.07**  **-5.14** | **7.15**  18.99 | **-13.64**  **8.30** | **-0.20**  10.38 | **Mean** | 24.11 | **-45.09** | 10.70 | **-16.00** | | **-4.07** |

* Results are presented as percent cell growth promotion.

* Values less than 10% growth promotion are presented in bold.

**Table S3: *In vitro* NCI 60 cell line anticancer screening results of compounds (5b, 5d, 7e, 8a, 8d, 8f, 8g, 8h, 8i & 13b) at single dose of 10 μM presented as percent cell growth promotion.**

| Panel/Cell Line | 5b | 5d | 7e | 8a | 8d | 8f | 8g | 8h | 8i | 13b |
| --- | --- | --- | --- | --- | --- | --- | --- | --- | --- | --- |
| Leukemia  CCRF-CEM  HL-60(TB)  K-562  MOLT-4  RPMI-8226  SR | 103.12  102.89  102.10  97.31  104.44  94.95 | 90.95  102.77  100.79  83.48  89.35  86.34 | 40.21  80.06  61.10  36.08  55.63  38.47 | 96.87  107.55  110.30  98.63  100.96  102.08 | 102.23  96.63  106.14  106.18  107.04  102.81 | 102.72  113.97  100.18  108.68  96.92  100.59 | 103.32  103.11  115.93  110.50  104.39  107.29 | 99.65  110.23  110.26  102.08  100.71  95.10 | 102.09  108.28  101.01  96.07  103.82  92.08 | 66.01  67.32  93.16  N/A  87.74  82.82 |
| Non-Small Cell Lung Cancer  A549/ATCC  EKVX  HOP-62  HOP-92  NCI-H226  NCI-H23  NCI-H322M  NCI-H460  NCI-H522 | 100.66  N/A  91.79  97.54  77.91  98.46  109.04  101.43  96.77 | 97.94  N/A  88.80  55.36  76.26  95.03  111.61  96.41  94.73 | 61.53  N/A  71.07  93.48  79.11  84.87  82.41  78.22  63.79 | 99.63  N/A  107.44  103.83  100.15  104.58  113.25  104.65  99.80 | 100.43  N/A  110.27  102.24  98.08  104.78  113.62  107.58  99.42 | 94.07  N/A  100.46  112.38  102.75  104.13  100.59  104.04  93.81 | 99.29  N/A  97.06  95.91  95.24  102.53  112.45  105.33  94.50 | 88.97  N/A  100.11  112.12  90.14  100.60  120.92  101.10  88.96 | 92.29  N/A  99.80  119.07  98.72  105.95  107.42  103.32  94.54 | 78.23  62.48  93.28  104.23  81.42  89.01  99.06  98.07  52.50 |
| Colon Cancer  COLO 205  HCC-2998  HCT-116  HCT-15  HT29  KM12  SW-620 | 104.12  113.44  100.08  108.95  104.37  102.76  102.08 | 90.33  125.00  92.15  101.25  94.32  101.06  92.70 | 87.36  109.78  72.47  69.54  76.99  57.68  95.03 | 109.27  116.92  99.11  110.84  105.50  104.00  102.08 | 106.06  117.02  102.65  107.84  98.23  104.10  103.69 | 108.94  99.78  103.09  98.61  95.98  107.39  103.84 | 97.72  108.63  112.65  98.47  98.30  108.60  106.80 | 120.10  100.99  95.64  91.84  90.03  113.05  106.38 | 113.40  101.69  98.11  90.29  91.22  110.46  112.75 | 105.82  114.99  97.77  94.13  95.75  84.49  105.60 |
| CNS Cancer  SF-268  SF-295  SF-539  SNB-19  SNB-75  U251 | 98.05  101.82  N/A  104.47  98.06  99.49 | 90.01  104.68  N/A  92.02  81.20  81.02 | 70.55  91.83  N/A  84.08  47.27  60.02 | 104.16  102.13  N/A  102.85  104.44  99.27 | 102.79  106.90  N/A  113.07  104.58  96.94 | 101.06  94.17  N/A  107.18  101.30  96.04 | 107.58  96.02  N/A  104.97  98.14  96.73 | 110.27  92.70  N/A  99.86  108.60  96.20 | 105.88  87.83  N/A  100.77  113.97  98.57 | 85.84  91.64  84.86  103.09  74.03  86.66 |
| Melanoma  LOX IMVI  MALME-3M  M14  MDA-MB-435  SK-MEL-28  SK-MEL-5  UACC-257  UACC-62 | 99.86  99.61  99.68  102.80  114.13  99.78  92.99  89.82 | 91.92  98.95  97.11  101.78  106.32  78.70  97.43  95.86 | 78.14  92.60  79.40  79.17  88.26  70.24  83.89  45.80 | 98.66  109.49  110.29  104.97  115.55  102.99  95.84  102.94 | 97.47  109.37  102.33  104.25  108.44  97.51  99.00  99.57 | 106.33  102.02  104.47  97.40  113.30  97.52  92.41  109.16 | 102.67  114.03  94.84  99.52  108.85  98.10  92.96  109.52 | 105.88  103.06  95.05  93.54  104.25  92.88  95.34  99.19 | 102.07  107.11  98.56  99.61  101.01  97.04  98.51  101.37 | 86.21  92.33  103.08  93.89  93.58  105.52  95.55  89.89  75.73 |
| Ovarian Cancer  IGROV1  OVCAR-3  OVCAR-4  OVCAR-5  OVCAR-8  NCI/ADR-RES  SK-OV-3 | 101.65  100.69  105.82  112..34  96.15  102.88  87.34 | 101.02  93.28  105.30  98.51  93.11  96.02  83.46 | 90.91  69.82  70.77  119.12  76.04  72.79  69.51 | 119.57  104.89  106.79  128.13  97.65  102.05  104.75 | 116.24  99.94  109.27  105.20  97.79  108.96  103.94 | 108.99  112.08  97.80  95.04  102.44  108.60  101.30 | 112.09  108.49  103.54  98.21  97.13  107.36  96.47 | 108.84  109.78  91.81  87.87  95.29  96.87  104.74 | 112.06  114.42  100.46  98.16  98.86  104.78  108.00 | 79.17  85.44  72.10  113.07  86.04  83.63  88.61 |
| Renal Cancer  786-0  A498  ACHN  CAKI-1  RXF 393  SN12C  TK-10  UO-31 | 106.81  98.67  111.96  94.57  115.04  100.36  116.14  78.63 | 109.40  88.94  111.40  86.32  98.74  90.98  109.44  57.84 | 83.90  51.51  80.72  79.66  81.98  79.10  84.79  51.36 | 106.39  105.00  114.90  96.10  101.64  109.76  109.81  91.35 | 103.55  97.78  115.42  108.56  108.23  102.35  104.97  96.00 | 93.29  76.51  106.81  90.31  107.83  100.92  90.61  92.23 | 100.35  71.73  100.38  95.26  104.83  100.48  97.25  107.89 | 90.25  65.35  98.42  86.85  99.82  100.40  88.69  95.92 | 92.37  N/A  99.63  94.80  98.58  104.22  83.19  102.32 | 114.48  102.55  80.73  99.07  99.70  91.44  109.32  53.94 |
| Prostate Cancer  PC-3  DU-145 | 91.56  113.58 | 72.64  101.68 | 38.97  81.39 | 92.56  104.50 | 101.38  99.03 | 100.95  100.32 | 99.43  111.19 | 98.99  106.90 | 100.32  110.75 | 67.76  86.83 |
| Breast Cancer  MCF7  MDA-MB 231/ATCC  HS 578T  BT-549  T-47D  MDA-MBA-468 | 92.95  93.31  104.82  106.63  97.57  101.43 | 84.53  73.03  89.72  92.23  79.82  103.76 | 72.69  52.88  76.42  71.99  57.90  68.60 | 102.79  107.72  112.75  111.77  99.99  97.68 | 105.33  107.61  108.80  103.78  101.10  93.33 | 92.06  113.82  106.23  66.68  87.22  103.56 | 96.67  127.47  105.12  75.55  90.54  98.82 | 86.47  120.61  110.06  N/A  82.33  84.75 | 89.07  117.24  108.69  N/A  95.97  87.37 | 86.20  67.52  97.93  96.85  70.54  84.94 |
| Mean | 100.80 | 93.03 | 72.80 | 104.90 | 104.15 | 100.54 | 101.90 | 99.05 | 101.38 | 88.71 |

**Table S 4**: ***In vitro* NCI 5 log dose results for compounds (6b, 6e, 7b & 13a) in μM against 60 cell panel.**

| **Panel** | **Cell Line** | **Compound 6b** | | | **Compound 6e** | | | **Compound 7b** | | | **Compound 13a** | | |
| --- | --- | --- | --- | --- | --- | --- | --- | --- | --- | --- | --- | --- | --- |
|  |  | **GI_50_** | **TGI** | **LC_50_** | **GI_50_** | **TGI** | **LC_50_** | **GI_50_** | **TGI** | **LC_50_** | **GI_50_** | **TGI** | **LC_50_** |
| **Leukemia** | **CCRF-CEM**  **HL-60(TB)**  **K-562**  **MOLT-4**  **RPMI-8226**  **SR** | 2.57  2.42  1.68  2.09  2.50  3.89 | 17.0  7.00  26.4  6.36  8.75  23.3 | >100  >100  >100  >100  >100  >100 | 2.38  2.03  2.20  2.36  2.60  2.56 | 5.99  4.18  4.67  6.00  6.48  9.55 | >100  8.64  9.92  >100  >100  >100 | **0.32**  1.08  **0.37**  **0.37**  **0.48**  **0.40** | ND  13.4  >100  >100  4.58  >100 | >100  >100  >100  >100  >100  >100 | 4.44  2.70  6.21  2.60  4.45  1.93 | >25  >25  >25  11.4  24.3  8.26 | >25  >25  >25  >25  >25  >25 |
| **Non-Small Cell Lung Cancer** | **A549/ATCC**  **EKVX**  **HOP-62**  **HOP-92**  **NCI-H226**  **NCI-H23**  **NCI-H322M**  **NCI-H460**  **NCI-H522** | 3.03  2.95  3.00  2.55  2.46  3.07  5.00  2.91  1.32 | 19.3  25.3  14.9  7.26  12.5  14.4  15.0  11.9  8.81 | >100  >100  74.9  >100  43.2  71.8  >100  56.8  43.2 | 3.24  3.47  1.81  2.56  3.61  2.38  3.31  3.46  1.84 | 8.59  17.9  3.70  6.60  15.9  6.31  1.25  11.7  3.88 | 37.9  87.8  7.56  42.0  64.6  40.9  69.5  39.1  8.21 | 2.81  1.61  1.09  1.64  **0.62**  **0.75**  2.60  2.79  **0.19** | 11.5  4.50  4.32  4.37  5.42  2.87  9.51  8.32  0.41 | >100  >100  47.7  17.1  >100  9.61  34.9  45.5  0.91 | 3.47  2.31  2.52  3.09  1.95  3.72  2.19  2.67  2.80 | 18.4  12.1  65.4  11.3  9.25  9.44  >25  8.65  9.68 | >25  >25  17.0  >25  >25  24.0  >25  >25  >25 |
| **Colon Cancer** | **COLO 205**  **HCC-2998**  **HCT-116**  **HCT-15**  **HT29**  **KM12**  **SW-620** | 2.77  3.26  2.46  1.59  2.65  1.47  3.16 | 9.58  19.8  12.0  11.8  14.1  11.4  18.6 | 34.6  >100  37.4  71.5  60.0  45.0  81.8 | 2.22  2.84  1.86  1.91  1.78  1.99  2.15 | 5.29  8.09  3.59  4.53  3.67  4.53  4.76 | 19.2  27.8  6.93  13.9  7.56  11.1  11.9 | 1.18  1.84  **0.32**  **0.96**  **0.46**  **0.88**  **0.34** | 2.92  3.48  1.20  3.08  2.39  2.65  1.06 | 7.21  6.57  4.65  9.67  >100  7.38  30.3 | 5.24  4.20  3.06  3.54  5.26  5.51  8.41 | >25  12.2  9.77  12.9  >25  >25  >25 | >25  >25  >25  >25  >25  >25  >25 |
| **CNS Cancer** | **SF-268**  **SF-295**  **SF-539**  **SNB-19**  **SNB-75**  **U251** | 3.19  2.84  2.88  3.67  1.88  2.86 | 28.1  11.0  11.5  19.0  8.67  10.3 | >100  45.8  36.6  >100  31.8  37.2 | 2.57  2.04  1.61  2.77  1.91  1.98 | 7.17  4.21  3.32  8.72  5.49  3.75 | 47.0  8.68  6.83  56.6  21.3  7.07 | 1.35  2.54  **0.28**  1.74  **0.45**  1.01 | 7.21  6.27  1.13  4.79  1.95  2.38 | 70.2  36.9  3.81  58.3  5.20  5.61 | 2.89  2.07  2.81  1.67  1.08  2.14 | 7.57  5.63  6.40  8.46  5.90  5.20 | 19.8  13.5  14.6  >25  21.3  11.4 |
| **Melanoma** | **LOX IMVI**  **MALME-3M**  **M14**  **MDA-MB-435**  **SK-MEL-2**  **SK-MEL-28**  **SK-MEL-5**  **UACC-257**  **UACC-62** | **0.82**  3.94  1.52  2.14  2.70  3.04  2.31  3.41  **0.95** | 14.6  16.5  11.5  9.42  7.70  12.8  6.82  10.8  7.97 | 53.2  50.0  40.7  30.9  44.8  51.5  24.7  33.5  38.1 | 1.75  2.17  1.76  1.73  2.22  1.63  1.85  2.39  1.75 | 3.27  4.79  3.57  3.43  4.59  3.20  3.50  5.30  3.59 | 6.11  20.0  7.27  6.81  9.50  6.29  6.65  1.48  7.35 | **0.16**  **0.44**  1.12  **0.29**  1.57  **0.42**  1.02  **0.15**  1.08 | 0.45  2.31  3.16  1.07  3.65  1.65  2.81  4.04  3.46 | 1.63  9.69  8.93  5.65  8.50  5.33  6.56  12.9  14.0 | 3.46  1.62  6.15  7.54  3.90  3.57  4.56  3.96  6.84 | 6.08  5.93  >25  >25  13.212.7  18.6  >25  >25 | 13.4  16.0  >25  >25  >25  >25  >25  >25  >25 |
| **Ovarian Cancer** | **IGROV1**  **OVCAR-3**  **OVCAR-4**  **OVCAR-5**  **OVCAR-8**  **NCI/ADR-RES**  **SK-OV-3** | 2.52  2.57  1.97  4.15  3.12  2.79  3.05 | 30.7  10.3  8.17  34.3  12.4  26.3  17.7 | >100  70.0  >100  >100  >100  >100  >100 | 3.98  1.87  2.79  2.10  3.35  3.63  2.61 | 19.5  3.99  8.36  4.99  9.32  14.9  6.59 | >100  8.47  58.2  18.7  47.4  >100  30.7 | 1.16  **0.35**  **0.75**  1.89  1.10  2.63  1.74 | 5.00  1.15  3.49  4.47  13.6  >100  5.48 | >100  3.95  17.0  12.6  >100  >100  27.2 | 3.32  2.64  1.73  5.61  2.60  2.63  1.46 | 7.92  5.97  12.2  15.3  6.53  6.39  5.51 | 18.9  13.5  >25  >25  16.4  15.5  16.6 |
| **Renal Cancer** | **786-0**  **A498**  **ACHN**  **CAKI-1**  **RXF 393**  **SN12C**  **TK-10**  **UO-31** | 3.09  3.79  1.12  1.97  4.28  4.58  5.03  1.90 | 25.2  18.8  7.12  12.5  21.4  37.2  24.4  15.8 | >100  >100  >100  88.0  >100  >100  >100  >100 | 1.71  3.21  1.84  3.49  2.17  2.86  3.19  2.14 | 3.34  10.5  3.69  12.8  4.13  9.41  9.42  6.01 | 6.55  38.5  7.41  43.6  7.84  60.1  33.7  31.8 | **0.87**  1.50  1.05  2.72  **0.31**  1.03  2.12  1.29 | 2.35  3.06  2.33  8.70  1.05  3.58  4.28  2.67 | ND  6.26  5.18  43.9  3.74  >100  8.64  ND | 3.60  3.45  3.47  3.34  2.69  6.72  6.08  3.98 | 8.18  13.1  18.7  14.3  6.24  >25  14.9  19.6 | 18.6  >25  >25  >25  14.5  >25  >25  >25 |
| **Prostate Cancer** | **PC-3**  **DU-145** | 2.32  3.17 | 11.0  26.9 | 55.4  >100 | 3.49  4.77 | 11.9  19.5 | 62.7  66.9 | 1.64  **0.73** | 6.08  2.08 | 35.6  4.88 | 3.26  3.01 | 10.6  10.5 | >25  >25 |
| **Breast Cancer** | **MCF7**  **MDA-MDA 231/ATCC**  **HS 578T**  **BT-549**  **T-47D**  **MDA-MB-468** | 1.43  3.40  3.83  2.15  1.03  2.57 | 8.32  15.9  27.7  9.28  5.35  9.47 | 64.1  64.9  >100  >100  79.3  >100 | 2.21  2.38  2.52  2.21  2.06  2.84 | 10.9  5.94  6.40  5.55  5.08  7.43 | 68.7  33.9  >100  66.0  56.4  36.2 | **0.32**  **0.39**  **0.69**  2.08  **0.24**  **0.75** | 1.89  3.68  47.0  7.43  7.68  2.52 | ND  42.4  >100  58.1  >100  7.18 | 1.32  4.76  2.12  3.06  **0.63**  **7.58** | 6.32  12.3  10.4  10.8  10.8  7.56 | 20.6  >25  >25  >25  >25  >25 |

**Table S 5**: ***In vitro* NCI 5 log dose results for compounds (13c, 16a, 16d & 17a) in μM against 60 cell panel.**

| **Panel** | **Cell Line** | **Compound 13c** | | | **Compound 16a** | | | **Compound 16d** | | | **Compound 17a** | | |
| --- | --- | --- | --- | --- | --- | --- | --- | --- | --- | --- | --- | --- | --- |
|  |  | **GI_50_** | **TGI** | **LC_50_** | **GI_50_** | **TGI** | **LC_50_** | **GI_50_** | **TGI** | **LC_50_** | **GI_50_** | **TGI** | **LC_50_** |
| **Leukemia** | **CCRF-CEM**  **HL-60(TB)**  **K-562**  **MOLT-4**  **RPMI-8226**  **SR** | **0.20**  **0.37**  **0.63**  **0.49**  **0.33**  **0.32** | >17.5  6.06  4.27  4.18  1.51  1.42 | >17.5  >17.5  >17.5  >17.5  >17.5  >17.5 | 3.53  3.71  1.71  1.71  2.10  1.75 | 17.6  13.0  14.5  8.75  7.71  12.3 | >25  >25  >25  >25  >25  >25 | 2.73  3.78  1.49  1.10  1.48  **0.97** | 13.4  13.1  13.1  7.57  7.51  8.85 | >25  >25  >25  >25  >25  >25 | 2.80  3.56  1.85  1.06  1.37  1.23 | 14.2  11.9  11.9  6.70  6.96  11.2 | >25  >25  >25  >25  >25  >25 |
| **Non-Small Cell Lung Cancer** | **A549/ATCC**  **EKVX**  **HOP-62**  **HOP-92**  **NCI-H226**  **NCI-H23**  **NCI-H322M**  **NCI-H460**  **NCI-H522** | 1.10  **0.64**  1.98  **0.35**  2.14  **0.90**  1.91  **0.89**  **0.49** | 8.30  6.49  5.75  1.96  7.20  4.36  5.62  3.44  3.46 | >17.5  >17.5  16.7  7.80  >17.5  15.9  16.5  9.89  >17.5 | 3.17  1.61  2.86  **0.77**  2.52  2.50  3.54  3.21  **0.68** | 20.0  15.0  8.84  6.29  7.98  16.4  18.3  9.98  4.62 | >25  >25  >25  >25  >25  >25  >25  >25  18.5 | 1.93  **0.58**  2.06  **0.32**  **0.95**  1.22  1.40  2.69  **0.32** | 9.42  6.40  8.48  2.91  6.82  6.74  7.85  7.15  3.31 | >25  >25  >25  >25  >25  24.6  >25  19.0  13.1 | 2.87  **0.67**  2.51  **0.33**  1.56  1.56  1.56  2.70  **0.35** | 12.3  7.32  7.74  3.09  6.58  7.87  8.20  7.12  4.04 | >25  >25  23.9  >25  21.7  >25  >25  18.8  16.7 |
| **Colon Cancer** | **COLO 205**  **HCC-2998**  **HCT-116**  **HCT-15**  **HT29**  **KM12**  **SW-620** | **0.97**  **0.87**  1.05  **0.76**  **0.77**  **0.70**  1.92 | 3.90  3.48  3.51  3.53  3.14  2.97  4.90 | 12.1  10.4  8.55  11.1  11.5  9.84  12.5 | **0.91**  4.85  1.20  2.99  1.46  3.05  3.84 | 5.39  1.59  5.02  9.39  6.36  9.91  11.1 | 20.5  >25  13.3  >25  21.2  >25  >25 | **0.49**  3.44  **0.88**  1.78  **0.34**  **0.13**  2.98 | 4.01  8.79  4.20  7.56  3.62  6.52  8.13 | 15.4  22.5  11.2  >25  12.0  18.6  22.2 | **0.45**  3.81  1.07  2.05  **0.42**  2.65  2.86 | 4.38  8.36  4.83  6.75  4.25  7.72  7.79 | 16.1  18.3  13.1  19.7  17.4  22.4  21.2 |
| **CNS Cancer** | **SF-268**  **SF-295**  **SF-539**  **SNB-19**  **SNB-75**  **U251** | **0.79**  **0.65**  1.38  1.26  1.61  1.31 | 5.50  3.70  4.01  4.90  5.63  3.93 | >17.5  15.6  9.94  1.61  >17.5  9.87 | 3.28  2.98  3.67  4.71  0.87  3.28 | 14.5  8.77  8.10  22.7  7.05  7.33 | >25  >25  17.8  >25  >25  16.4 | 2.05  1.94  3.07  3.50  **0.29**  2.78 | 10.5  8.24  6.56  13.0  5.27  6.54 | >25  >25  14.0  >25  23.1  >25 | 2.23  1.65  3.12  3.20  **0.49**  1.24 | 12.9  6.70  7.58  12.0  6.65  7.47 | >25  20.5  18.4  >25  >25  19.1 |
| **Melanoma** | **LOX IMVI**  **MALME-3M**  **M14**  **MDA-MB-435**  **SK-MEL-2**  **SK-MEL-28**  **SK-MEL-5**  **UACC-257**  **UACC-62** | **0.68**  **0.96**  **0.70**  **0.79**  **0.69**  **0.93**  **0.98**  **0.95**  N/A | 2.82  4.00  3.16  3.23  3.24  3.54  3.25  3.37  N/A | 8.24  11.5  9.63  9.17  10.4  8.93  7.70  8.80  N/A | 1.57  2.66  1.74  1.47  2.16  3.67  2.08  2.74  1.25 | 4.97  9.92  6.65  7.88  5.83  10.5  5.27  6.87  5.70 | 11.4  >25  20.6  >25  14.5  >25  11.8  17.2  19.0 | 1.11  1.22  **0.85**  **0.73**  **0.70**  2.62  **0.81**  2.04  1.00 | 4.83  6.23  4.69  4.38  3.98  6.17  4.18  5.28  5.76 | 12.8  17.3  12.9  12.0  11.0  14.5  10.2  11.6  20.1 | 1.29  1.38  **0.91**  1.01  **0.97**  2.84  1.08  2.54  1.24 | 4.85  7.30  5.03  5.31  5.13  7.23  4.56  5.82  5.69 | 11.9  25.0  15.2  16.2  15.1  18.4  10.8  13.4  17.5 |
| **Ovarian Cancer** | **IGROV1**  **OVCAR-3**  **OVCAR-4**  **OVCAR-5**  **OVCAR-8**  **NCI/ADR-RES**  **SK-OV-3** | 1.04  **0.73**  **0.59**  1.24  1.30  **0.92**  2.66 | 5.85  4.46  6.01  4.26  9.50  9.95  6.70 | >17.5  >17.5  >17.5  12.2  >17.5  >17.5  16.8 | 1.93  3.51  3.00  4.92  4.63  4.12  3.17 | 10.7  8.69  >25  >25  16.8  17.0  11.4 | >25  21.5  >25  >25  >25  >25  >25 | 0.90  1.64  1.33  3.38  3.61  2.55  1.13 | 6.30  10.0  17.6  11.8  14.7  >25  8.19 | >25  >25  >25  >25  >25  >25  >25 | 1.14  2.87  2.09  3.88  3.49  2.62  2.38 | 6.37  9.80  >25  15.4  15.0  16.6  9.96 | >25  >25  >25  >25  >25  >25  >25 |
| **Renal Cancer** | **786-0**  **A498**  **ACHN**  **CAKI-1**  **RXF 393**  **SN12C**  **TK-10**  **UO-31** | **0.76**  **0.27**  **0.71**  **0.83**  1.17  **0.94**  2.32  **0.17** | 3.01  1.70  3.80  6.50  3.87  3.85  6.55  4.19 | 7.53  6.53  16.2  >17.5  9.81  10.8  >17.5  >17.5 | 4.01  1.45  3.10  N/A  2.60  3.95  N/A  1.36 | 12.5  6.60  >25  N/A  8.12  18.9  N/A  6.66 | >25  17.6  >25  N/A  >25  >25  N/A  23.8 | 2.22  1.27  1.56  N/A  2.16  2.52  N/A  0.60 | 7.63  4.42  24.4  N/A  6.11  11.0  N/A  5.28 | 24.4  11.5  >25  N/A  15.9  >25  N/A  >25 | 2.60  **0.68**  2.35  N/A  1.01  2.12  N/A  0.72 | 8.11  3.64  >25  N/A  4.97  8.07  N/A  5.20 | >25  10.6  >25  N/A  14.4  >25  N/A  23.9 |
| **Prostate Cancer** | **PC-3**  **DU-145** | **0.54**  1.27 | 2.51  4.08 | 7.71  11.0 | 14.4  >25 | 14.4  >25 | >25  >25 | 1.19  2.40 | 9.33  6.52 | >25  17.2 | 1.30  2.65 | 10.1  10.5 | >25  >25 |
| **Breast Cancer** | **MCF7**  **MDA-MDA 231/ATCC**  **HS 578T**  **BT-549**  **T-47D**  **MDA-MB-468** | **0.57**  **0.35**  1.72  **0.67**  **0.38**  **0.68** | 3.03  2.44  8.18  3.07  2.24  3.43 | 17.3  10.0  >17.5  7.80  10.5  12.5 | 26.8  1.95  2.95  2.93  1.17  1.90 | 11.1  8.73  12.9  8.20  8.28  11.5 | >25  >25  >25  22.9  >25  >25 | **0.93**  1.32  2.90  1.90  **0.64**  1.05 | 6.40  6.32  11.5  7.42  6.56  4.73 | 24.9  19.7  >25  24.8  >25  19.5 | 1.18  1.28  2.58  2.05  **0.88**  1.49 | 6.07  6.90  10.5  6.65  8.44  7.62 | 19.8  24.8  >25  19.0  >25  >25 |

*The GI_50_ value (growth inhibitory activity) corresponds to the concentration of the compound causing 50% decrease in net cell growth.

*The TGI value (cytostatic activity) is the concentration of the compound resulting in total growth inhibition.

*LC_50_ value (cytotoxic activity) is the concentration of the compound causing net 50% loss of initial cells at the end of the incubation period of 48 h.

*Submicromolar GI_50_ values are presented in bold.

**Table S 6**: **Cytotoxic activity of the new compounds against MCF-7, HCT-116 and HepG-2cancer cell lines.**

| **Cpd ID** | **R** | **R_1_** | **R_2_** | **X** | **IC_50_(μM)** | | | |
| --- | --- | --- | --- | --- | --- | --- | --- | --- |
|  |  |  |  |  | **MCF-7** | **HCT-116** | **HepG-2** |  |
| **5a** | **H** | **H** | **-** | **-** | **>1000** | **977.0** | **N/A** |  |
| **5c** | **CH_3_** | **H** | **-** | **-** | **22.9** | **15.0** | **N/A** |  |
| **6a** | **H** | **H** | **H** | **-** | **67.6** | **91.2** | **N/A** |  |
| **6c** | **H** | **4-Cl** | **3-CF_3_** | **-** | **6.2** | **6.0** | **N/A** |  |
| **6d** | **CH_3_** | **H** | **H** | **-** | **67.6** | **57.5** | **N/A** |  |
| **6f** | **CH_3_** | **4-Cl** | **3-CF_3_** | **-** | **3.2** | **3.1** | **N/A** |  |
| **7c** | **H** | **4-Cl** | **3-CF_3_** | **-** | **2.5** | **2.6** | **N/A** |  |
| **7d** | **CH_3_** | **H** | **H** | **-** | **7.8** | **7.2** | **N/A** |  |
| **7f** | **CH_3_** | **4-Cl** | **3-CF_3_** | **-** | **31.0** | **49.0** | **N/A** |  |
| **8b** | **H** | **phenyl** | **-** | **-** | **363.0** | **549.5** | **N/A** |  |
| **8c** | **H** | **2-fluorophenyl** | **-** | **-** | **32.4** | **42.7** | **N/A** |  |
| **8e** | **H** | **2-furoyl** | **-** | **-** | **190.5** | **513** | **N/A** |  |
| **8j** | **CH_3_** | **2-furoyl** | **-** | **-** | **478.6** | **478.6** | **N/A** |  |
| **12a** | **Cl** | **H** | **H** | **-** | **81.3** | **478.6** | **>1000** |  |
| **12b** | **Cl** | **H** | **Cl** | **-** | **155** | **173.7** | **173.8** |  |
| **12c** | **Cl** | **4-Cl** | **3-CF_3_** | **-** | **>1000** | **724.4** | **>1000** |  |
| **16b** | **-** | **-** | **-** | **Cl** | **91** | **28.8** | **77.6** |  |
| **16c** | **-** | **-** | **-** | **CH_3_** | **>1000** | **257** | **>1000** |  |
| **17b** | **-** | **-** | **-** | **CH_3_** | **13.5** | **38** | **39** |  |
| **Dox** |  |  |  |  | **0.9** | **0.62** | **0.5** |  |

**
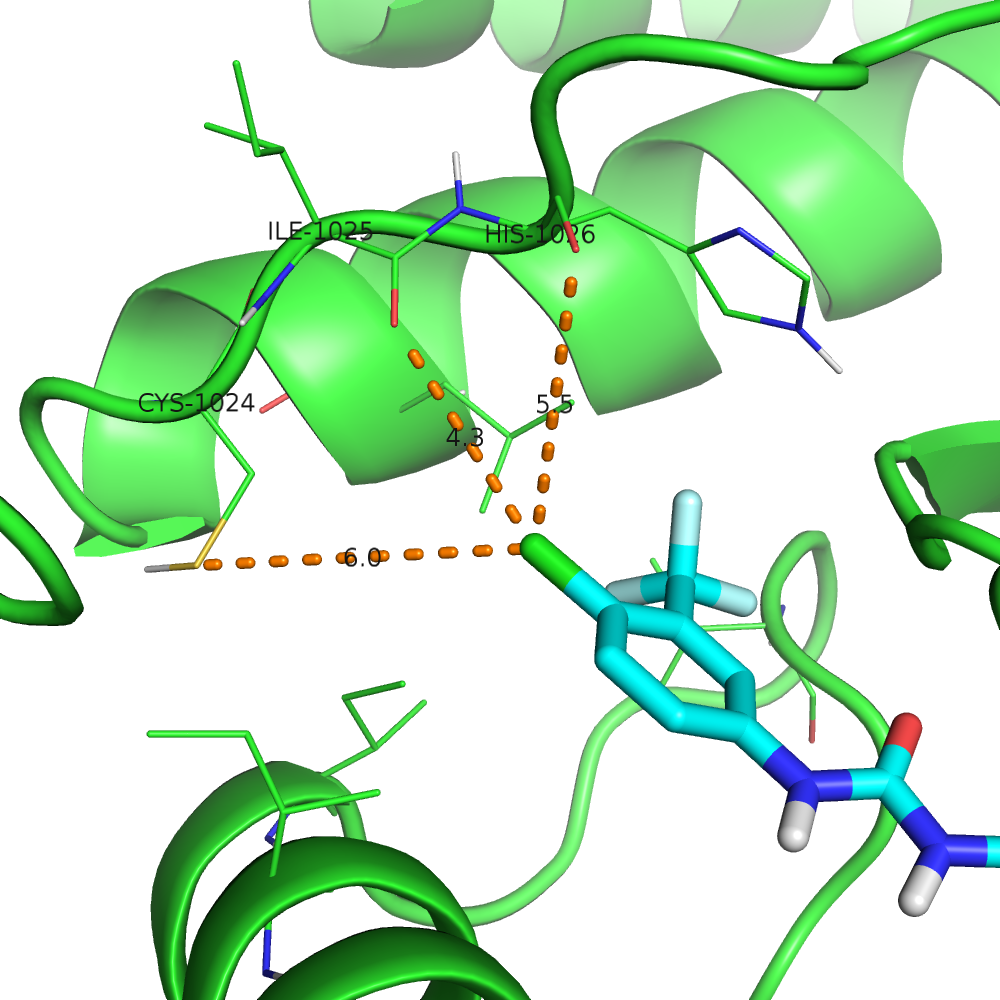
**

*Figure S7. The X-ray structure of Sorafenib (cyan sticks) in the binding site of VEGFR2 (green) (PDB: 4ASD). The chloro atom of Sorafenib is in the far proximity to the sulfur atom of CYS-1024 and oxygen atoms of the backbone of ILE-1025 and HIS-1026. This would exclude the chance of having a reliable halogen-bonding contact. This also was confirmed by calculating a very low score for XBScore*[*^3^*](#_ENREF_3) *(score of 0.077) using the webpage of (http://www.halogenbonding.com), for the chloro atom of Sorafenib and the nearest backbone oxygen (of ILE-1025). All non-polar hydrogen atoms were omitted for clarity.*

**
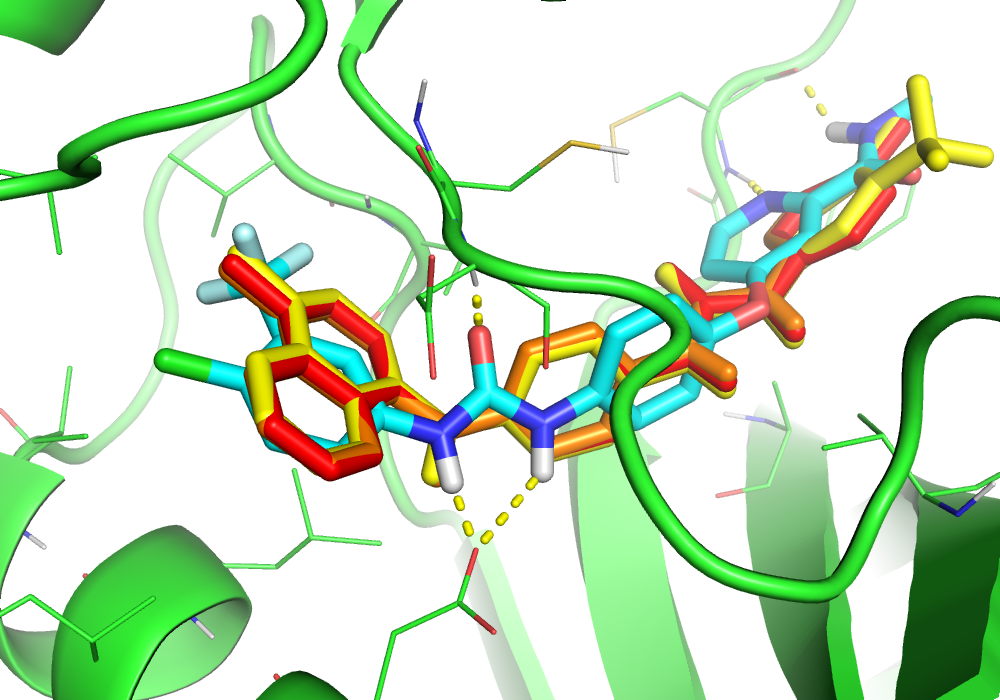
**

*Figure S8. Overlay of the docked poses of compounds 6d, 6e and 6f (gold, red and yellow sticks, respectively) on the co-crystallized ligand Sorafenib (cyan sticks) in the binding site of VEGFR2 (PDB:4ASD). The yellow dashed-lines represent the polar contacts (H-bonding interactions) of Sorafenib. All presented docked poses are the best-scored.*

**
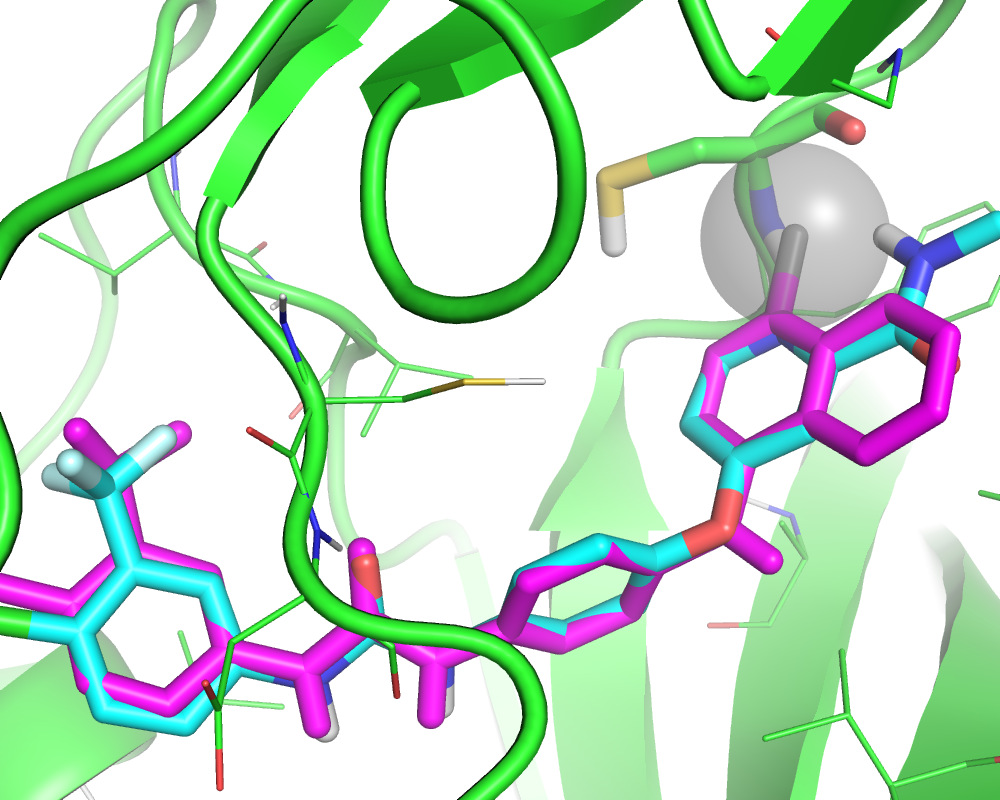
**

*
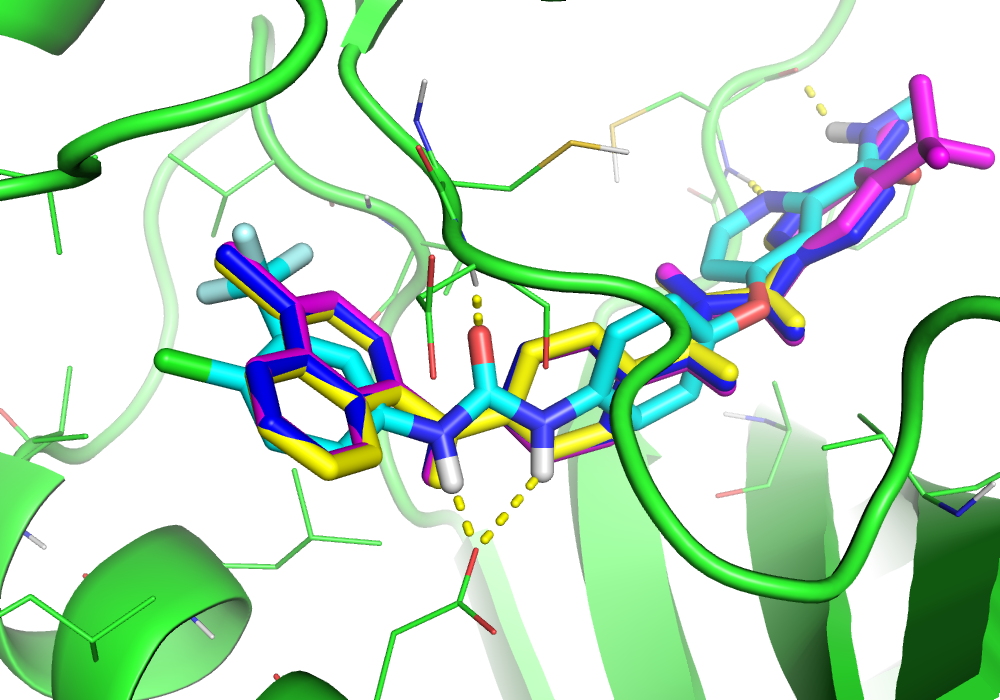
Figure S 9. Overlay of the docking pose of 6c (magenta sticks) on the co-crystallized ligand Sorafenib (cyan sticks) in the binding site of VEGFR2 (PDB code: 4ASD). An imaginary chloro group (grey atom/sphere) was introduced to the docking pose of 6c which shows an obvious clash with the backbone of CYS-919. This explains the 180 degree flip of the best-scored docking poses of 12b, 12c and 13c. The grey sphere is depicted to represent vdW radius of the chloro group.*

*Figure S 10. Overlay of the docked poses of compounds 12a, 12b and 12c (yellow, blue and magenta sticks, respectively) on the co-crystallized ligand Sorafenib (cyan sticks) in the binding site of VEGFR2 (PDB:4ASD). The yellow dashed-lines represent the polar contacts (H-bonding interactions) of Sorafenib. All presented docked poses are the best-scored.*

**6. Experimental**

***6.2. Biological Evaluation***

***6.2.1. In vitro VEGFR tyrosine kinase activity at single dose of 10* μM *concentration.***

The VEGFR tyrosine kinase activity at single dose concentration of 10 μM was carried out by BPS Bioscience (www.bpsbioscience.com). VEGFR (BPS#40301) served as the enzyme source and Poly (Glu, Tyr) sodium salt, (4:1, Glu:Tyr) (Sigma#P7244) served as the standardized substrate & Kinase-Glo Plus Luminescence kinase assay kit (Promega#V3772). The IC_50_ determination was carried out where quality control testing is routinely performed on each of the targets to insure compliance to acceptable standards. ^33^P-ATP was purchased from Perkin Elmer and ADP-Glo^TM^ was purchased from Promega. All other materials were of standard laboratory grade.

***6.2.1.1. Assay protocols*.**

The VEGFR tyrosine kinase activity was performed using Kinase-Glo Plus luminescence kinase assay kit (Promega). It measures kinase activity by quantitating the amount of ATP remaining in solution following a kinase reaction. The luminescent signal from the assay is correlated with the amount of ATP present and is inversely correlated with the amount of kinase activity. The compounds were diluted to 100 μM in 10% DMSO and 5 ml of the dilution was added to a 50 ml reaction so that the final concentration of DMSO is 1% in all of reactions. All of the enzymatic reactions were conducted at 30 °C for 40 min. The 50 μl reaction mixture contains 40 μM Tris, pH 7.4, 10 μM MgCl_2_, 0.1 mg/ml BSA, 0.2 mg/ml Poly (Glu, Tyr) substrate, 10 μM ATP and VEGFR. After the enzymatic reaction, 50 ml of Kinase-GloPlus Luminescence kinase assay solution (Promega) was added to each reaction and incubate the plate for 5 min at room temperature. Luminescence signal was measured using a BioTek Synergy 2 microplate reader. The protein kinase assays used to determine IC_50_ value were performed using ADP-GloTM assay kit from Promega which measures the generation of ADP by the protein kinase. Generation of ADP by the protein kinase reaction leads to an increase in luminescence signal in the presence of ADP-GloTM assay kit. The assay was started by incubating the reaction mixture in a 96-well plate at 30 °C for 30 min. After the 30 min incubation period, the assay was terminated by the addition of 25 ml of ADP-GloTM Reagent (Promega). The 96 well plate was shaken and then incubated for 40 min at ambient temperature. 50 ml of Kinase detection reagent was added, the 96-well reaction plate was then read using the ADP-Glo Luminescences Protocol on a GloMax plate reader (Promega: Catalog #E7031). Blank control was set up that included all the assay components except the addition of appropriate substrate (replace with equal volume of kinase assay buffer). The corrected activity for each protein kinase target was determined by removing the blank control value.

***6.2.1.2. Data analysis*.**

VEGFR activity assays were performed in duplicate at each concentration. The luminescence data were analyzed using the computer software, Graphpad Prism. The difference between luminescence intensities in the absence of VEGFR (Lu_t_) and in the presence of VEGFR (Lu_c_) was defined as 100% activity (Lu_t_ - Lu_c_). Using luminescence signal (Lu) in the presence of the compound, % activity was calculated as: % Activity = {(Lu_t_ - Lu)/(Lu_t_ - Lu_c_) X100%, where Lu = the luminescence intensity in the presence of the compound (all percent activities below zero were set to 0%). % Inhibition was calculated as: % inhibition =100 (%) - % activity. IC_50_ determination for inhibitor against VEGFR was estimated by generating a graph of log inhibitor vs normalized response with variable using the Prism software.

### *In vitro HUVEC Anti-proliferative assay*

The *in vitro* HUVEC proliferative assay for the synthesized compounds was also carried out in BPS Bioscience Corporation, San Diego, CA, USA ([www.bpsbioscience.com](http://www.bpsbioscience.com)).

The assay was performed at single dose concentration of 10 µM, where HUVEC umbilical vein endothelial cells, human (Life Technologies # C-003-5C) served as the cells’ source, in Medium 200 (Life Technologies # M-200-500), with large vessel endothelial supplement (LVES) (Life Technologies # A14608-01) and Pen-step (Hyclone # SV30010). AlamarBlue (Life Technologies # DAL1025) was used as the fluorescent reagent.

#### *Assay protocol*

HUVEC cells were cultured in Medium 200 with 2% LVES and 1% Pen-strep. To perform the proliferation assay, HUVEC cells were seeded at 5000 cells/ 50μl /well in a 96-well black clear-bottom tissue culture plate. Cells were incubated at 37°C and 5% CO2 overnight to allow them to recover and reattach.

Next day cells were treated with test compounds for 72 hours. After treatment, cell proliferation was measured by Fluorescent quantitation of alamarBlue reagent. The alamarBlue assay incorporates a fluorometric/colorimetric growth indicator based on detection of metabolic activity. Specifically, resazurin, the active ingredient in the alamarBlue reagent, is blue in color and virtually non-fluorescent. Upon entering cells, resazurin is reduced to resorufin, a compound that is red in color and highly fluorescent. Continued cell growth maintains a reduced environment, therefore increasing the overall fluorescence and color of the media surrounding cells. The fluorescence intensity of alamarBlue reagent was shown to be directly proportional to cell number. To perform the alamarBlue assay, 10 μl of alamarBlue reagent was added to each well and the plate was incubated at 37°C for an additional 2 hours. Fluorescence intensity was measured at an excitation of 530 nm and an emission of 590 nm using a BioTek Synergy^TM^ 2 microplate reader.

#### *Data analysis*

Cell proliferation assays were performed in triplicate at each concentration. The fluorescent intensity data were analyzed using the computer software, Graphpad Prism. In the absence of the compound, the fluorescent intensity (F_t_) in each data set was defined as 100%. In the absence of cells, the fluorescent intensity (F_b_) in each data set was defined as 0%. The percent cell in the presence of each compound was calculated according to the following equation: %cell = (F-F_b_)/(F_t_-F_b_), where F= the fluorescent intensity in the presence of the compound, F_b_= the fluorescent intensity in the absence of cells, and F_t_ = the fluorescent intensity in the absence of the compound.

The values of % cell versus a series of compound concentrations were then plotted using non-linear regression analysis of Sigmoidal dose-response curve generated with the equation Y=B+(T-B)/1+10^((LogEC50-X)×Hill Slope)^, where Y=percent cell, B=minimum percent cell, T=maximum percent cell, X= logarithm of compound and Hill Slope=slope factor or Hill coefficient.

- - 1. ***Evaluation of cytotoxic activity against NCI 60 human cancer cell lines panel.***

***6.2.3.1 Assay protocol***

The human tumor cell lines of the cancer-screening panel were grown in RPMI 1640 medium containing 5% fetal bovine serum and 2 μM L-glutamine. For a typical screening experiment, cells are inoculated into 96 well microtiter plates in 100 ml at plating densities ranging from 5000 to 40,000 cells/well depending on the doubling time of individual cell lines. After cell inoculation, the microtiter plates are incubated at 37 °C, 5% CO_2_, 95% air and 100%

relative humidity for 24 h prior to addition of experimental drugs. After 24 h, two plates of each cell line are fixed in situ with TCA, to represent a measurement of the cell population for each cell line at the time of drug addition (Tz). Experimental drugs are dissolved in dimethyl sulfoxide at 400-fold the desired final maximum test concentration and stored frozen prior to use. At the time of drug addition, an aliquot of frozen concentrate is thawed and diluted to twice the desired final maximum test concentration with complete medium containing 50 mg/ml Gentamicin. Additional four, 10-fold or ½log serial dilutions are made to provide a total of five drug concentrations plus control. Aliquots of 100 ml of these different drug dilutions are added to the appropriate microtiter wells already containing 100 ml of medium, resulting in the required final drug concentrations. Following drug addition, the plates are incubated for an additional 48 h at 37 °C, 5% CO_2_, 95% air, and 100% relative humidity. For adherent cells, the assay is terminated by the addition of cold TCA. Cells are fixed in situ by the gentle addition of 50 ml of cold 50% (w/v) TCA (final concentration,10% TCA) and incubated for 60 min at 4 °C. The supernatant is discarded, and the plates are washed five times with tap water and air dried. Sulforhodamine B (SRB) solution (100 ml) at 0.4% (w/v) in 1% acetic acid is added to each well, and plates are incubated for 10 min at room temperature. After staining, unbound dye is removed by washing five times with 1% acetic acid and the plates are air dried. Bound stain is subsequently dissolved with 10 μM trizma base, and the absorbance is read on an automated plate reader at a wavelength of 515 nm. For suspension cells, the methodology is the same except that the assay is terminated by fixing settled cells at the bottom of the wells by gently adding 50 ml of 80% TCA (final concentration, 16% TCA).

***6.2.3.2. Data analysis***

Using the seven absorbance measurements [time zero, (Tz), control growth, (C), and test growth in the presence of drug at the five concentration levels (Ti)], the percentage growth is calculated at each of the drug concentrations levels. Percentage growth inhibition is calculated as: [(Ti -Tz)/(C - Tz)] x 100 for concentrations for which Ti>/ ¼ Tz and [(Ti -Tz) / Tz] x100 for concentrations for which Ti <Tz.

Three dose response parameters are calculated for each experimental agent. Growth inhibition of 50% (GI_50_) is calculated from [(Ti -Tz)/(C - Tz)] x 100 =50, which is the drug concentration resulting in a 50% reduction in the net protein increase (as measured by SRB staining) in control cells during the drug incubation. The drug concentration resulting in total growth inhibition (TGI) is calculated from Ti ¼ Tz. The LC_50_ (concentration of drug resulting in a 50% reduction in the measured protein at the end of the drug treatment as compared to that at the beginning) indicating a net loss of cells following treatment is calculated from [(Ti - Tz) / Tz] x100 = -50. Values are calculated for each of these three parameters if the level of activity is reached; however, if the effect is not reached or is exceeded, the value for that parameter is expressed as greater or less than the maximum or minimum concentration tested [25, 26].

### *In vitro Anti-proliferative activity against MCF-7, HCT-116 and HepG-2 cancer cell lines*

- - - 1. ***Assay Protocol***

Cells were used when 90% confluence was reached in T25 flasks. 5,000 cells in fresh medium were seeded in 96-well microlitre plates and left to attach for 24h. Cells were incubated with the tested compounds at concentration range from 0,1,2.5,5,10 µg/ml and incubation was continued for48h. After 48h treatment,the cells were fixed with 50µl cold 50% trichloroacetic acid (TCA )for 1h at 4ºC. Wells were then washed 5 times with water and stained for 30 min at room temperature with 50µl of 0.4%SRB dissolved in 1% acetic acid. The wells were then washed 4 times with 1% acetic acid. The plates were air dried and the dye was solubilized with 100µl/well of 10mM tris base (pH 10.5) for 5min on a shaker (Orbital Shaker OS 20,Boeco,Germany) at 1600 rpm. The optical density (O.D.)of each well was measured spectrophotometrically at 564 nm with an enzyme linked immunosorbent assay( ELIZA) micraplate reader (Tecan Sunrise,Austria).The mean back ground absorbance was subtracted automatically and mean values for each drug concentration was calculated.

- - - 1. ***Assay Analysis***

The percentage of cell survival was calculated as follows:

Survival fraction =O.D.(treated cells)/O.D.(control cells)

The IC_50_ value (the concentration required to produce 50% inhibition of cell growth) was calculated using sigmodial dose response curve-fitting models (GraphPad,Prizm software incorporated). Each concentration was repeated 3 times.

- - 1. ***Cell cycle analysis***

MCF-7 and HCT-116 cells at a density of 4 x10^6^ cell/ T 75 flask were exposed to compounds **7b, 13c** and **16a** respectively at its GI_50_ concentration for 24 and 48 h. The cells then were collected by trypsinization, washed with phosphate buffered saline (PBS), and fixed in ice-cold absolute alcohol. Thereafter, cells were stained, using Cycletest^TM^ Plus DNA Reagent Kit (BD Biosciences, San Jose, CA), according to the manufacturer’s instructions. Cell cycle distribution was determined using a FACS Calibur flow cytometer (BD Biosciences, San Jose, CA).

### *Measurement of apoptosis using annexin-V-FITC apoptosis detection kit*

Apoptosis was determined by staining cells with Annexin V–fluorescein isothiocyanate (FITC) and counterstaining with propidium iodide (PI) using the Annexin V–FITC/PI apoptosis detection kit (BD PharMingen, San Diego, CA, USA) according to the manufacturer's instructions. Briefly, 4 x10^6^ cell/ T 75 flask were exposed to compounds **7b, 13c &16a**  at its GI_50_ concentration for 24 and 48 h. The cells then were collected by trypsinization and 0.5 × 10^6^ cells were washed twice with phosphate-buffered saline (PBS) and stained with 5 μl Annexin V–FITC and 5 μl PI in 1× binding buffer (BD PharMingen) for 15 minutes at room temperature in the dark. Analyses were performed using FACS Calibur flow cytometer (BD Biosciences, San Jose, CA).

- - 1. ***Immunostaining and morphological studies***

MCF7 cells were cultured on sterile cover slips (Harvard Apparatus, 22 mm2) in sterile six well plates at a density of 2×10^5^ cells/well. Twenty-four hours after seeding, cells were exposed to GI_50_ concentration of compound **7b** in fresh serum-free medium. At the end of the exposure, cells attached to cover slips were imaged with a light inverted microscope or washed with PBS and fixed with 3.7% paraformaldhyde for 10 min, permeabilized with 0.1% Triton X-100 in TBST containing 0.01% Tween 20 for 10 min, and blocked for 1 h with 5% goat serum in TBST. The fixed and permeabilized cells were incubated with cleaved caspase-3 rabbit mAb (Cell signaling technology, MA), at a dilution of 1:500, in blocking solution overnight at 4ºC, followed by secondary Cy3-goat anti-Rabbit antibody (Jackson Immuno Research, West Grove, PA) 1:1000 dilution, in the blocking solution for 1 hour, at room temperature. 4',6'-diamidino-2-phenylindole, dihydrochloride (DAPI) (Sigma– Aldrich, St. Louis, MO) was used as counter stain to stain the DNA. The cover slips with cells were then mounted on a glass slide with anti-fade mounting medium, and viewed with an epifluorescence microscope, Leica, DM 5500 B (Leica, Buffalo Grove, IL), at a magnification of 60×.

***6.3. Docking studies.***

***6.3.1. Preparation of VEGFR2 crystal structure.***

Coordinates for VEFGR2 crystal structure was retrieved from the Protein Data Bank (PDB code: 4ASD) and handled consequently with Molecular Operating Environment program (MOE)[35]. Non-essential ions, water molecules and ligand were discarded. Bond orders, formal charges and explicit hydrogen atoms were added to the complex structure. Subsequently, the most appropriate protonation states and optimization of the H-bond network were performed with the MOE ‘Protonate 3D’ function at standard settings (T = 300 K, pH = 7.0, ionic strength I = 0.1 mol/l). Prepared protein structure was saved as PDB file which were further converted to PDBQT file by employing a *python* script (*prepare_receptor4.py*) provided by the MGLTools package (version 1.5.4).[36, 37] The native geometry of the binding site was preserved without in-place ligand-protein minimization.

***6.3.2. Preparation of synthesized compounds for docking***

The molecules were built and prepared by MOE. ‘Molecule wash’ function was used to generate meaningful protonation states by deprotonating strong acids and protonating strong bases (if required). Energy minimization of all molecules was then performed using the MMFF94x force field at a gradient of 0.01 RMSD (i.e. if the gradient falls below RMSD, the minimization terminates). Existing chirality was preserved (if required) and partial charges were calculated according to the standard parameters of the force field. The compounds were saved as SD file which were further converted and split into PDBQT files by open-Babel (version 2.3.1)[38].We enabled the rotation of ligand amide groups by assigning the appropriate flag in the PDBQT files.

***6.3.3. Docking experiments.***

The docking experiments were performed using AutoDock Vina (version 1.1.2)[28].We employed the default docking parameters; however with increased exhaustiveness of the docking algorithm “exhaustiveness = 30”. This approach was accommodated to compensate the increased degrees of freedom of the compounds that resulted by enabling the amide bonds to be rotatable. The size of the docking grid was generally 21 Å × 16.5 Å × 19.5 Å, with a grid spacing of 1 Å. In this case ligand binding site was completely included in the grid box. By default, the docking was terminated when the maximum energy difference between the best-scored pose and the worst one was 3 kcal/ mol. The docking experiments were conducted three times to extract simple descriptive statistics (e.g., mean and standard deviation). This docking approach was validated by successful pose-retrieval of the co-crystallized ligand (sorafenib) when docked into its corresponding binding site of the crystal structure.
